# A systematic review of the biological mediators of fat taste and smell

**DOI:** 10.1152/physrev.00061.2021

**Published:** 2022-09-15

**Authors:** Rosario B. Jaime-Lara, Brianna E. Brooks, Carlotta Vizioli, Mari Chiles, Nafisa Nawal, Rodrigo S. E. Ortiz-Figueroa, Alicia A. Livinski, Khushbu Agarwal, Claudia Colina-Prisco, Natalia Iannarino, Aliya Hilmi, Hugo A. Tejeda, Paule V. Joseph

**Affiliations:** ^1^Section of Sensory Science and Metabolism Unit, Division of Intramural Research, National Institutes of Health, National Institute of Alcohol Abuse and Alcoholism, U.S. Department of Health and Human Services, Bethesda, Maryland; ^2^Section of Sensory Science and Metabolism, Division of Intramural Research, National Institute of Nursing Research, National Institutes of Health, U.S. Department of Health and Human Services, Bethesda, Maryland; ^3^NIH Library, Office of Research Services, Office of the Director, National Institutes of Health, U.S. Department of Health and Human Services, Bethesda, Maryland; ^4^Section of Neuromodulation and Synaptic Integration, Division of Intramural Research, National Institute of Mental Health, National Institutes of Health, U.S. Department of Health and Human Services, Bethesda, Maryland

**Keywords:** chemosensation, fat taste, obesity, oleogustus, olfaction

## Abstract

Taste and smell play a key role in our ability to perceive foods. Overconsumption of highly palatable energy-dense foods can lead to increased caloric intake and obesity. Thus there is growing interest in the study of the biological mediators of fat taste and associated olfaction as potential targets for pharmacologic and nutritional interventions in the context of obesity and health. The number of studies examining mechanisms underlying fat taste and smell has grown rapidly in the last 5 years. Therefore, the purpose of this systematic review is to summarize emerging evidence examining the biological mechanisms of fat taste and smell. A literature search was conducted of studies published in English between 2014 and 2021 in adult humans and animal models. Database searches were conducted using PubMed, EMBASE, Scopus, and Web of Science for key terms including fat/lipid, taste, and olfaction. Initially, 4,062 articles were identified through database searches, and a total of 84 relevant articles met inclusion and exclusion criteria and are included in this review. Existing literature suggests that there are several proteins integral to fat chemosensation, including cluster of differentiation 36 (CD36) and G protein-coupled receptor 120 (GPR120). This systematic review will discuss these proteins and the signal transduction pathways involved in fat detection. We also review neural circuits, key brain regions, ingestive cues, postingestive signals, and genetic polymorphism that play a role in fat perception and consumption. Finally, we discuss the role of fat taste and smell in the context of eating behavior and obesity.

## Graphical abstract

**Figure:**
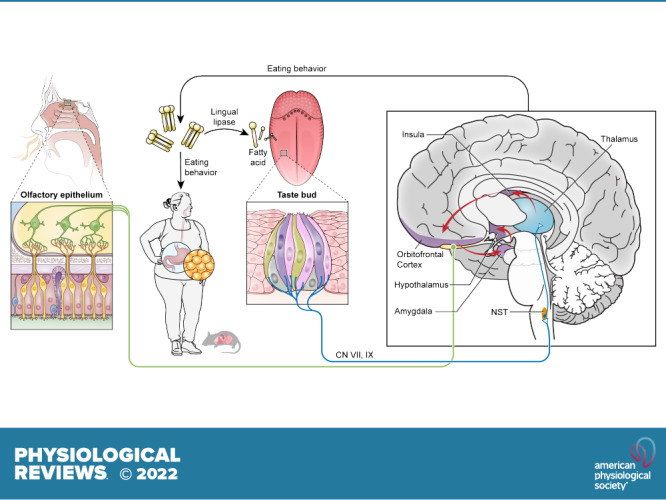

CLINICAL HIGHLIGHTS Taste and smell play an integral role in maintaining health, including mental health and nutritional status.

- Both taste and smell contribute to flavor perception.
- Taste and smell screening is important for early diagnosis of disease processes and management of the adverse effects of chemosensory disturbances.
- Fats are sources of essential fatty acids and are important mediators of energy balance and cellular homeostasis.
- Both animals and humans exhibit a preference for high-fat foods.
- In addition to texture, taste and smell are integral to fat perception.
- Current candidates for fat taste and smell receptors are CD36 and GPR120.
- Fat taste transduction involves Ca^2+^ signaling as well as secondary messenger cascades, such as MAP kinases.
- The most frequently studied genetic polymorphisms associated with fat chemosensation are CD36 polymorphisms. The rs1761667, rs1527483, rs2312018, and rs3840546 SNPs of CD36 are reportedly associated with fat perception.
- Individuals with obesity reportedly display fat chemosensory dysfunction.

## 1. INTRODUCTION

### 1.1. Health and Clinical Implications of Taste and Smell (Olfaction): COVID-19 and Beyond

Taste and smell (i.e., the chemical senses) play an integral role in maintaining health, including mood, social behaviors, nutritional intake, detecting hazards (e.g., smell of smoke or contaminated foods), and other essential survival/physiological mechanisms. Taste and smell dysfunction are known to have a negative impact on emotional well-being and mental health (1, 2) and are positively correlated with increased anxiety, depression, and reductions in health-related quality of life (1). Despite the well-documented negative impact of taste and smell dysfunction, the chemical senses have often been overlooked in the context of health and disease. The COVID-19 pandemic highlighted the importance of taste and smell impairments, with millions of people world-wide reporting COVID-19-related taste and smell dysfunction (1, 3). For systematic reviews on COVID-19-related taste and smell dysfunction, see Refs. 4–6. COVID-19-related taste and smell dysfunction was soon accompanied by patients reporting depression, anxiety, and even detachment from reality. For example, a 2020 qualitative study captured a patient’s experience “I feel sad and depressed and distanced to the world as it used to be. Not being able to smell the most mundane things, like the rain or my boyfriend’s perfume. Not being able to participate socially like I used to” (7). While taste and smell symptoms were reported, acutely, persistent smell impairment was associated with more COVID-19 symptoms, and some have postulated that it may be a key marker for long-COVID illness (8). In COVID-19-related smell loss, SARS-CoV-2 may target sustentacular cells (rather than olfactory sensory neurons or the olfactory bulb) and others have shown that there is also a downregulation of olfactory receptors and signaling molecules (9–12). It has been recently suggested that COVID-19-related taste loss may arise from a direct infection of tastebud cells by SARS-CoV-2, which impairs taste receptor stem-cell activity (13). Importantly, taste and smell dysfunction are prevalent in other disease processes beyond COVID-19 (e.g., Parkinson’s and Alzheimer’s). In fact, the identification of chemosensory dysfunction in other illnesses can aid in early diagnosis. Thus screening for chemosensory dysfunction is important for health diagnosis and management.

Taste and smell also play an integral role in our ability to identify, find, and preferentially consume nutrients necessary to maintain health, including fats. Taste and smell are the first sensory signals along the gut-brain axis to detect appetitive food cues, including the perception of fat (14, 15). This is exemplified by our predilection for energy-dense foods, including fats. However, while fats and other energy-dense nutrients are essential for survival, chronic overconsumption of these foods can lead to obesity. Thus there is a growing interest in the role of fat taste (also known as oleogustus) and smell in the pathogenesis of obesity, including the study of the biological mediators of fat taste and smell as potential targets in pharmacologic and nutritional interventions. Fats are the most energy-dense macronutrient (16). They are sources of essential fatty acids and important mediators of energy balance and cellular homeostasis (17). Palatable cues associated with fat perception activate reward circuitry in the brain [such as the nucleus accumbens (NAc) and prefrontal cortex (PFC), among others], motivating eating behavior (18). Deciphering the biological mechanisms of fat taste and smell constitutes an important step in addressing obesity as a public health challenge.

### 1.2. Measurement and Assessment of Taste and Smell Function

There are several methods used to measure taste and smell. Chemosensory screening can be used to assess smell or taste disorders. For example, smell/olfactory and taste screening tools include the NIH Toolbox (which assesses sensory function), NHANES Pocket Smell Test (PST), the SCENTinel rapid smell test, Sniffin’ Sticks 12-item Screening test, The University of Pennsylvania Smell Identification Test (UPSIT), and the Global Consortium for Chemosensory Research (GCCR) Smell and Taste Challenge. Other tests measure odor/tastant identification, discrimination, and detection threshold. For a comprehensive summary of tools and measures, see Refs. 19, 20. In the context of eating behavior, studies examining the role of fat taste and smell often use a combination of psychophysical methods to measure taste and smell (e.g., sensitivity, preference/liking, and intensity). Taste and olfactory sensitivity are measured by determining threshold: the concentration at which a smell or taste is detected or recognized (19–22). Preference is a measure of an individuals’ most preferred concentration of a tastant (taste-invoking chemical molecule) or olfactory cue (23). In addition to preferred concentrations, a taste or odor intensity may also contribute to what an individual perceives as pleasant foods. Sensitivity, intensity, and preference for tastants and odors can impact food choices in normal weight and individuals with obesity (24, 25).

### 1.3. Anatomy and Physiology of Taste and Smell

**FIGURE 1.**
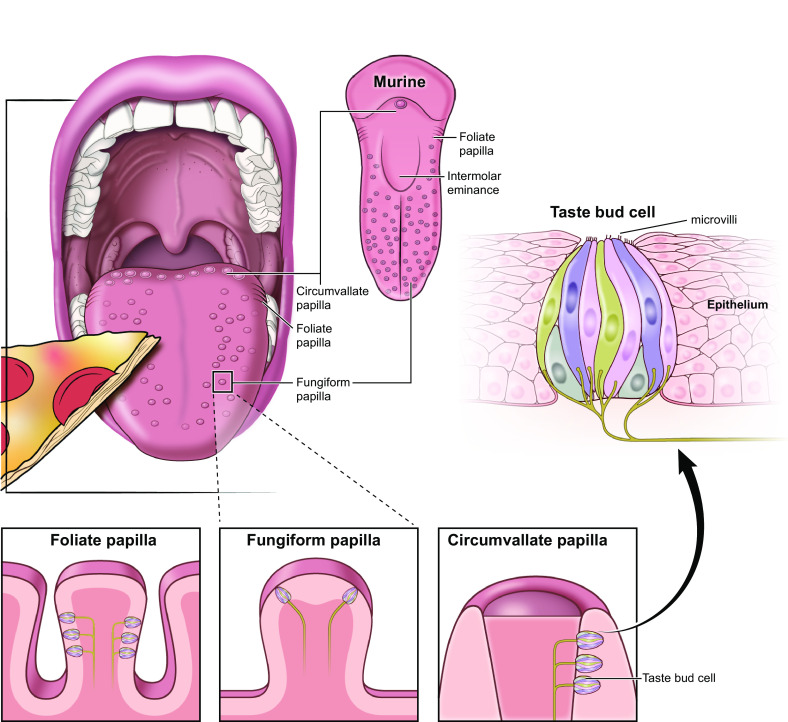
Taste bud cell. Taste buds are onion-shaped clusters of taste receptor cells. They are found in the tongue, the soft palate, the pharynx, and the esophagus. There are three different kinds of papillae that contain taste bud cells: foliate, fungiform, and circumvallate papillae. Food particles that dissolve in liquids and/or saliva (tastants) bind to microvilli to stimulate taste receptors and initiate transduction cascades that give rise to tate.

**FIGURE 2.**
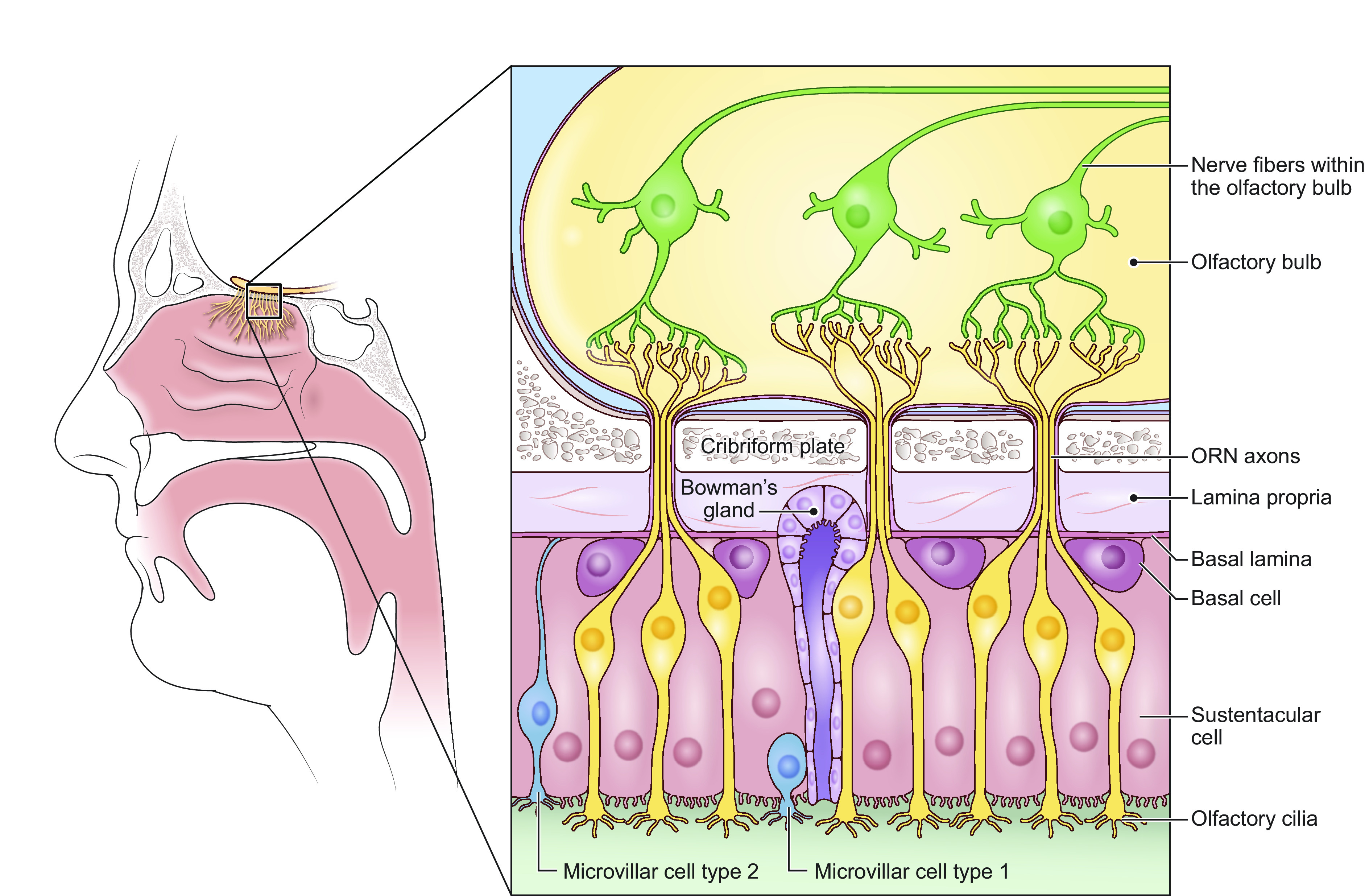
Olfactory epithelium and olfactory bulb. The olfactory epithelium is located within the nasal cavity and is comprised of olfactory sensory neurons (ORNs), basal cells, and supporting cells (i.e., sustentacular and microvillar cells). ORN dendrites project to the mucus layer that lines the olfactory epithelium. Smell/odors stimulate the olfactory cilia initiating olfactory signal transduction.

Importantly, what humans perceive as flavor encompasses more than taste; olfaction also plays an essential role in our perception of foods and beverages. Stimulation of chemoreceptor cells in both the mouth [e.g., taste bud cells (TBCs)] and nose (e.g., olfactory receptor cells) are processed by our central nervous system to contribute to flavor perception (26). Taste receptor cells are found in onion-shaped structures called taste buds, which are found on the surface of the tongue and form taste papillae (FIGURE 1). The olfactory epithelium lines the nasal cavity and is made up of olfactory receptor cells, basal, and supporting cells (FIGURE 2). Both taste and smell contribute to flavor perception, making it difficult for most people to discern what is exclusively taste and/or smell. For example, smell impairments induced by inflammation of olfactory tissue following a respiratory infection are often reported as a loss of taste (27, 28). In turn, flavor (rather than taste or smell alone) contributes to pleasurable qualities associated with food.

In addition to taste and olfaction, other sensory information contributes to our experience of flavor, such as food texture, irritation, and temperature (e.g., heat or spiciness). Somatosensory receptors such as mechanoreceptors, nociceptors, and thermoreceptors are responsible for our ability to discern or perceive these additional qualities, respectively (29–32). Fatty foods often have unique textural properties (e.g., creaminess) and can dissolve capsaicin and other spicy ingredients that do not easily dissolve in water. Thus it is crucial to study and understand how taste, smell, and associated chemical properties interact and contribute to fat perception and eating behavior.

### 1.4. Fat Taste as an Emerging Taste Modality

**FIGURE 3.**
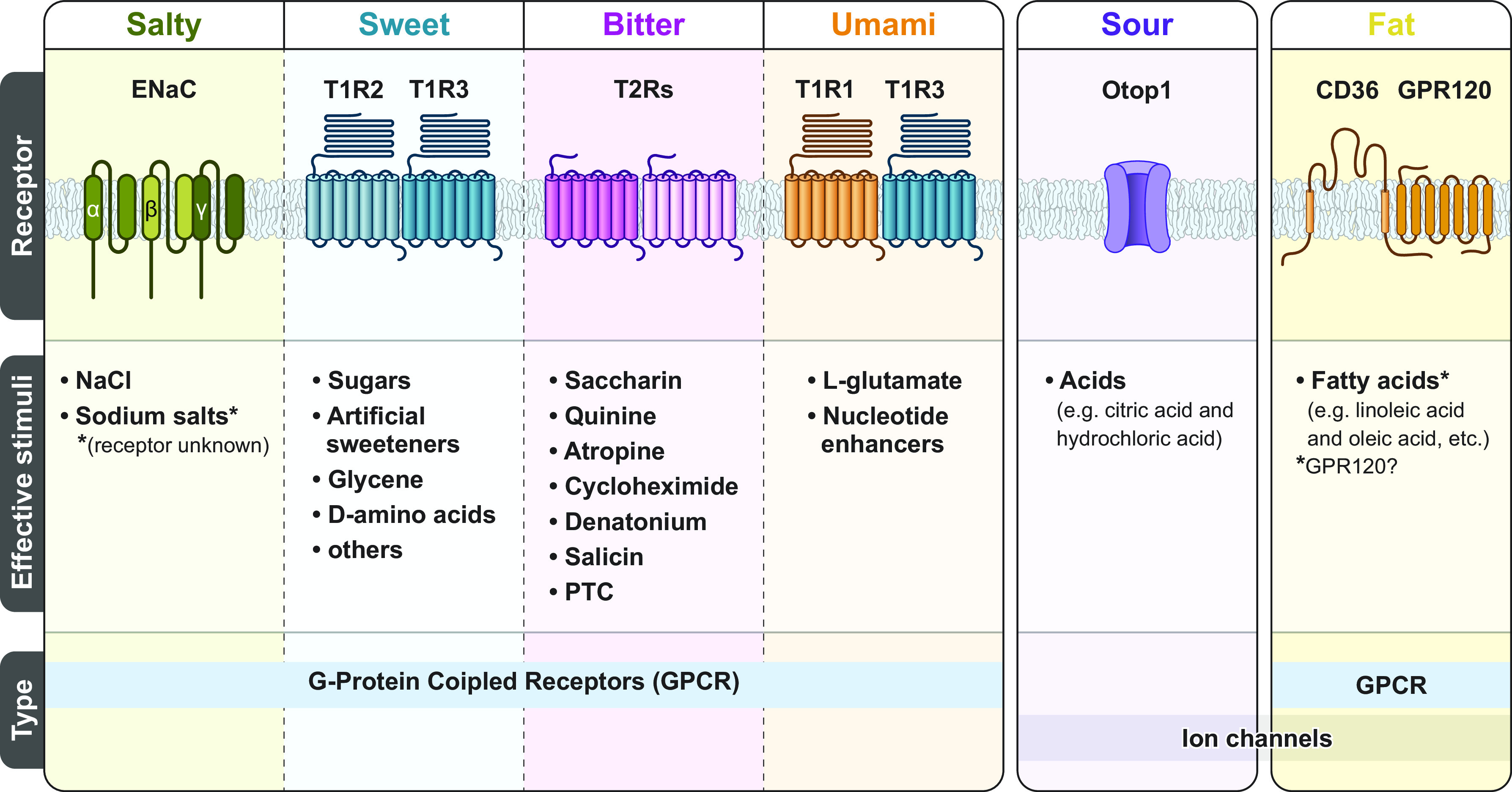
Taste receptors by taste modality. There are five commonly recognized primary taste modalities: salty, sweet, bitter, sour, and umami. However, there is growing evidence suggesting fat may be an additional taste modality. Each primary taste modality is characterized by multiple elements including having dedicated receptors [i.e., G protein-coupled receptors (GPCRs) and ion channels] and a defined class of effective stimuli. CD36, cluster of differentiation 36; ENaC, epithelial sodium channel; PTC, phenylthiocarbamide. Figure adapted from Ref. 26, with permission from Springer Nature.

**FIGURE 4.**
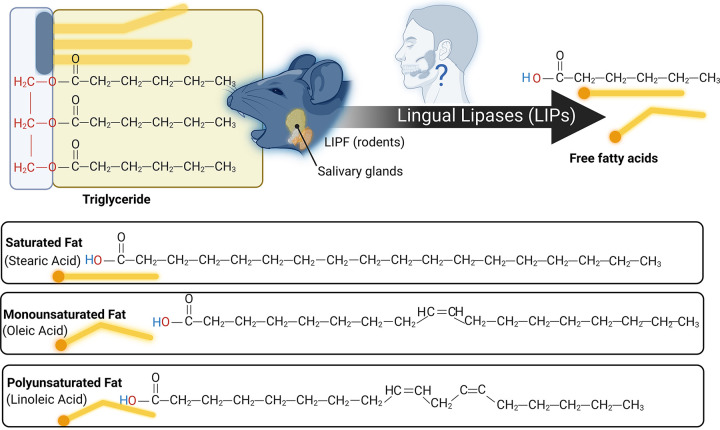
Triglyceride breakdown and fatty acid types. Triglycerides are a common type of dietary fat, composed of three fatty acids joined to glycerol. Lingual lipase can breakdown dietary triglycerides into fatty acids and glycerol. Rodents synthesize salivary lipase (LIPF). Humans also have salivary lipase forms (e.g., LIPK, LIPM, LIPN). Studies selected in this review often studied human and animal mode responses to common fatty acids, including oleic acid and linoleic acid. Image created with BioRender.com, with permission.

Literature exploring taste has primarily focused on five taste modalities: sweet, bitter, salty, sour, and umami (e.g., fish sauce, meat extract, and other savory foods). Each taste modality is characterized by specific criteria, including having an effective stimulus, a unique combination of taste-bud cell receptors specific to that taste modality (FIGURE 3), and taste perception that is independent of other taste modalities. There is emerging literature examining fat taste as an additional taste modality (for reviews, see Refs. 18, 33–35). Preclinical studies strongly suggest that free fatty acids (FFAs) (36) are the effective stimuli responsible for fat taste (18, 34). There are three types of FFAs (differing in degree of saturation): monosaturated FFAs (e.g., oleic acid), polyunsaturated FFAs (e.g., linoleic acid), and saturated FFAs (e.g., stearic acid) (FIGURE 4). Rodents specifically detect FFAs independently of other taste modalities, textural, and postingestive cues (37, 38). In contrast, the role of FFAs in human fat taste perception is not well established, as naïve subjects cannot easily distinguish FFAs (39). FFAs are also poorly soluble in saliva (compared to other tastants), and there are individual variations in fatty acid perception (34, 39, 40). Nonetheless, humans can be trained to detect FFAs (39, 40). Humans also possess salivary lipase isoforms (LIPK, LIPM, and LIPN), which break down triglycerides into FFAs (41) and can detect long-, medium-, and short-chain fatty acids (39, 40). Thus FFA detection may play a role in fat perception. However, the mechanisms of FFA-mediated activation of taste-related cells are not fully understood, as it may involve other transduction mechanisms, genetic, and environmental factors.

The growing interest in fat chemosensation and the underlying mechanisms in the context of obesity has led to a significant and rapid rise in studies examining the biological mediators of fat taste and smell. For example, since 2016, 88 articles discussing “fat taste” (in the title and abstract) have been indexed in PubMed alone. (For reviews, see Refs. 18, 35, 42–45.) While previous reviews have examined the biological mediators of fat taste, few reviews have examined both fat taste and smell. However, as mentioned above, taste and smell collectively contribute to flavor perception. Furthermore, there are few systematic reviews examining fat perception.

Systematic reviews provide a comprehensive and reproducible summary of available literature that examines a specific topic (46). Systematic reviews are conducted using a specific methodology that includes a succinct and clearly defined research question; predefined eligibility criteria and reproducible methods to select relevant articles; a comprehensive and systematic search of the published scholarly literature (and sometimes gray literature) to identify potentially relevant studies; study selection (i.e., screening) process conducted independently in pairs using the predefined eligibility criteria to minimize bias; an assessment of the internal and external validity (e.g., risk for bias) of the included articles; and a narrative synthesis of the findings from the included studies (46). If the specified methods are followed to ensure rigor and minimize bias, systematic reviews can serve as a valuable tool for researchers and clinicians to inform future research and shape clinical practice and guidelines. Therefore, as the number of studies examining fat chemosensation grows, conducting a systematic review of this field is increasingly important to provide a comprehensive overview of how these studies are contributing and expanding what we know about fat taste and smell.

Thus this systematic review will discuss studies that examine potential associations between taste and smell measures, biological mediators of fat taste and smell, and eating behavior in normal weight and individuals with obesity. The aim of this systematic review is to identify, analyze, and integrate the findings of emerging literature (2014–2021) to build on our understanding of the biological mechanisms of fat taste and olfaction. This knowledge can ultimately contribute to identifying potential therapeutic targets in nutritional and pharmacological interventions of fat-chemosensory dysfunction, as observed in obesity.

## 2. METHODS

The Preferred Reporting Items for Systematic Reviews and Meta-Analyses (PRISMA) guideline was used for reporting this review (47).

### 2.1. Eligibility Criteria

Our inclusion criteria were as follows: an original research article published in a peer-reviewed journal in English within the past 8 years (2014–2021) on biological mechanisms associated with fat taste and olfaction in human adults (individuals 18 years of age and older) and animals. Although taste and olfactory receptors exist outside the mouth and nose, this review focused on oral taste and nasal and retronasal olfaction. Studies had to discuss biological mechanisms associated with fat taste and olfaction, such as taste and olfactory threshold and/or preference, in relation to potential biological mediator(s) (e.g., fat taste preference in relation to brain activity, protein expression, or genetic polymorphisms). Exclusion criteria were articles published before 2014, studies including pediatric populations (17 years of age and younger) or nonadult animals (e.g., mice: postnatal day >35 days), descriptive studies, conference abstracts, case studies, studies focused only on a disease condition, nonprimary data articles (methods papers, opinions, proceedings), and any secondary data articles or reviews of any kind.

### 2.2. Information Sources and Search Strategy

Four databases: Embase (Elsevier), PubMed (US National Library of Medicine), Scopus (Elsevier), and Web of Science: Core Collection (Clarivate Analytics) were searched by a biomedical librarian (AAL). Search terms used were a combination of keywords and controlled vocabulary terms (e.g., MeSH in PubMed and EMTREE in Embase) for each concept of interest (i.e., fat, taste, smell, obesity), and search strategies were developed by the biomedical librarian in consultation with the review team. Searches were limited by publication year (2014–2021) and language (English). See final search strategies captured in Supplemental File S1 (all Supplemental material is available at https://doi.org/10.6084/m9.figshare.20415975.v1). The bibliographies of included articles were manually searched and screened by authors R. B. Jaime-Lara, B. E. Brooks, R. S. E. Ortiz-Figueroa, C. Vizioli, M. Chiles, and N. Nawal.

### 2.3. Study Selection

Before beginning, a pilot of 10 articles was conducted to test the eligibility criteria and screening process at both title and abstract and full text levels with all reviewers. After completing the pilot at both levels, all reviewers met to compare results, discuss the process, address questions and problems, and make final revisions to the criteria.

The titles and abstracts were first reviewed followed by the full text for those included after title and abstract screening. Two rounds of screening were conducted at each level (i.e., two rounds at title abstract level and two rounds at full text level. For both rounds, two reviewers (R.B.J.-L., B.E.B., R.S.E.O.-F., M.C., and N.N.) independently screened each article using the eligibility criteria and a third reviewer compared the results. Disagreements during screening were resolved by discussion between the two reviewers that screened and the third reviewer that checked initial screens to reach a consensus on whether to include or exclude the article. EndNote 20 (Clarivate Analytics) was used for the study selection process.

### 2.4. Data Collection and Data Items

A pilot of the data collection step was conducted on a sample set of three articles wherein the reviewers (R.B.J.-L., C.V., B.E.B., R.S.E.O-F., M.C., N.N., and N.I.) practiced extracting the specified data points and discussed questions or problems. For all articles included after full text screening, data collection was conducted using Microsoft Word and organized using Microsoft Excel. For each included study, sample size, participant/animal subject characteristics (e.g., age and sex), methods (intervention and/or test administered, biological samples collected, materials/fat sources used, etc.), and key findings about the biological mediators of fat taste or smell were collected. The extracted data is summarized in TABLES 1 and 2 and throughout the discussion. Two reviewers independently extracted the specified data points, and a third reviewer compared the data to identify discrepancies or errors, which were discussed by the third reviewer with the two reviewers who collected the data until a consensus was reached regarding the correct data to collect.

**Table 1.** Clinical study characteristics and findings

Author (Reference), Year, Country	Sample Size	Participant Characteristics	Methods	Key Findings
Andersen et al. (48), 2020, Denmark	*N* = 24 adults	Age = 26 ± 3.5 years	Analysis of neural taste responses to fat using EEG	Neural responses to the three fatty tastants (skim milk, whole milk, and cream) were significant and highly uniform. Neural activation occurred shortly (0.0 s) after the fatty stimulus was applied. They identified a neural correlate to fat taste.
		Sex = female (*n* = 15) and male (*n* = 9)		
		BMI = mean 23.5 ± 2.7 kg/m^2^		
Appelqvist et al. (49), 2016, Australia	*N* = 10 adults	Age = mean age of 44.5 ± 7.2 years	Oil-in-water emulsions (50%, 10%, and 2% fat) were used as model food systems.	There was an enhancement of fatty after feel intensity for 50% fat emulsions containing the more lipophilic aroma ethyl hexanoate compared to ethyl butanoate, indicating a cross-modal interaction.
		Sex = male (*n* = 2) and female (*n* = 8)	Particle size distribution was measured by laser light scattering.	
		Groups = solutions consisted of 2, 10, and 50% fat, with either HEX or BUT		
Bajit et al. (50), 2020, Morocco	*N* = 100 Moroccan adults	Age = mean 32.37 years ± 9.52 years	Anthropometric measurements	Analysis of the CD36 rs1761667 SNP in obese and normal weight subjects found a higher frequency of the AA genotype in obese subjects compared to the GG genotype. Obese subjects also had a higher OA detection threshold. The results for the OA detection threshold in the obese group, however, were very dispersed and did not correlate to a specific level of concentration, making the results inconclusive.
		Sex = female (*n* = 72) and male (*n* = 28)	Oleic acid sensitivity test	
		Groups = obese (mean BMI 34.97 ± 4.02 kg/m^2^) and normal weight (mean BMI 22.16 ± 1.81 kg/m^2^)	SNP analysis	
Besnard et al. (51), 2018, France	*N* = 38 Caucasian adults	Age = age-matched adults	Oral lipid detection threshold tests	Salivary flow and CA-IV were suspected to decrease oral sensitivity of fat in the obese nontaster group. Ultimately, specific microbial and salivary environments surrounding circumvallate papillae are involved in fat taste sensitivity.
			Oral microbiota analysis	
		Sex = all male	16S-targeted metagenomics	
			Bioinformatics pipeline	
		Groups = normal weight BMI <25.0 kg/m^2^ (*n* = 21) and obese BMI ≥30 kg/m^2^ (*n* = 17)	Saliva collection and analysis (flow, protein concentration, enzymatic activities, total antioxidant capacity, CA-VI levels, and LPS levels)	
Bricio-Barrios et al. (52), 2019, Mexico	*N* = 27 Mestizo adults	Age = 18–25 years old	Oral fatty acid detection threshold test	BMI positively correlates with the oral sensitivity threshold of fatty acids and negatively correlates with serum sCD36 levels. Obesity can lower fat taste sensitivity and impact serum CD36 levels.
		Sex = female (*n* = 51) and male (*n* = 21)	Anthropometric measures	
		BMI = normal weight (BMI 18.5 to 24.9 kg/m^2^) (*n* = 52) and overweight (BMI 25 to 29.9 kg/m^2^) (*n* = 20)	Serum CD36 quantification	
Burgess et al. (53), 2018, USA	*N* = 68 Caucasian (*n* = 36) and East Asian adults (*n* = 32)	Age = mean age 25.3 ± 0.8 years for Caucasians and 25.0 ± 0.9 for East Asians	Buccal swab-DNA extraction	Oral perception of fat was found to vary by CD36 genotype between Caucasians and East Asians. There was no main effect of CD36 genotype on oral detection of fatty acids and no interaction between CD36 rs1761667 genotype and ethnicity on threshold detection. East Asians who had the GG genotype gave higher fattiness and creaminess ratings to the samples compared to those who had the AA genotype. No effect of this SNP was observed on the perception of fat-associated attributes among Caucasian participants. Heightened ratings of fat perception among East Asians who were GG carriers may be due to increased CD36 protein expression although it is presently unclear why the same relationship was not observed in GG Caucasians in this study.
		Sex = 25 females, 11 males for Caucasians and 24 females, 8 males for East Asians	PCR amplification for CD36 target	
		BMI = BMIs ranged from 24.8 to 26.2 kg/m^2^ (Caucasians) and from 21.8 to 22.8 kg/m^2^ (East Asians)	Taste threshold: 3-Alternative Forced Choice (AFC)	
			Labeled Magnitude Scale (LMS)	
Chamoun et al. (54), 2021, Canada	*N* = 49 adults	Age = 25 ± 3 years	SNP genotyping	CD36 SNPs rs1527483 and rs3211908 were found to have significant associations with fat taste sensitivity.
		Sex = 35 females, 14 males	Fat taste sensitivity test with OA	
		BMI = 22.3 ± 2.6 kg/m^2^		
Chmurzynska et al. (55), 2020, Poland	*N* = 421 adults	Age = 20–40 years	Oral fat discrimination analysis	Participants with the GG CD36 genotype were more likely to be fat discriminators than were carriers of the A allele (*P* < 0.05). Polymorphisms of FFAR1, FFAR3, or CA6 were not related to fat discrimination. Fat discrimination was not associated with fat intake and polymorphisms of CD36, FFAR1, FFAR4, or CA6 were not associated with frequency of HF food consumption.
		Sex = 207 females, 214 males	Genotyping of SNPs	
		BMI = controls <25 kg/m^2^ (*n* = 208), experimental group >25 kg/m^2^ (*n* = 213)	Assessment of food intake and high-fat-food consumption	
Costanzo et al. (56), 2018, Australia	*N* = 88 (44 twin pairs) Australian adults	Age = mean age 43.7 ± 15.5 years	Three 2-h laboratory sessions 4 weeks apart.	The 8-wk consumption of an LF diet increases sensitivity to FT and the same period with an HF diet attenuates sensitivity, regardless of body weight. There is little indication of genetic contribution to FT.
			Measured: *1*) taste detection threshold to oleic acid (FATT), *2*) ability to rank the amount of fat in food, 3) liking ratings for high-fat and reduced-fat foods, and *4*) intensity ratings to 5 prototypical tastants	
			Dietary intervention: participants given and taught about the HF or LF diet and nutrition labels to identify which foods for their diet.	
		Sex = 33 female pairs, 10 male pairs, and 1 gender discordant pair.	Detection threshold of OA: 3-AFC test	
			FT (fat taste) rank	
		BMI = BMIs at baseline ranged from 21.2 to 31.4 kg/m^2^ (LF diet) and from 21 to 32.6 kg/m^2^ (HF diet)	TG (triglyceride) ranking task	
			Fatty food liking: LMS	
Costanzo et al. (56), 2019, Australia	*N* = 18 (9 pairs) Australian adults	Age = mean age 41·6 ± 16.5 years	Participants attended two tasting sessions	No significant time-diet interactions were observed for CD36, GPR84, FFAR2, and KCNA2 expression.
			Two biopsy sessions	A significant negative association was observed between Δ FATT and Δ FFAR4. There was a positive association between Δ FATT and Δ GPR84. Significant negative associations were observed between Δ FFAR4 and Δ fat intake, Δ saturated fat intake, Δ monosaturated fat intake, and Δ polyunsaturated fat intake. There was a significant positive association between Δ FFAR2 and Δ dietary fiber intake.
		Sex = 16 females, 2 males	A diet booklet for each diet was created with the aid of an accredited practicing dietitian	No significant associations were observed for CD36 or GPR84.
			Food recall and records were analyzed for carbohydrate, protein, fat, and fiber intake (g and % of energy) using computer software FoodWorks (version 8; Xyris)	There was a statistical trend for a negative association between KCNA2 and polyunsaturated fat intake.
		BMI = BMIs ranged from 20.6 to 33.2 kg/m^2^	Fungiform papillae biopsy	
			The gene expression of FAT receptors: RT-PCR	
Eldeghaidy et al. (57), 2016, United Kingdom	*N* = 16 Adults	Age = mean age 25 ± 2 years	gLMS	Compared to the WL, consuming HFM led to decreased anterior insula taste activation in response to fat-related satiety. HFM caused reduced amygdala activation in response to the FS compared to the CS. Baseline cerebral blood flow significantly reduced in taste, homeostatic, and reward areas after the HFM. An individual’s plasma CCK concentration correlated negatively with brain activation in taste and oral somatosensory and reward areas.
		Sex = 6 females, 11 males	Two emulsion stimuli were delivered during fMRI	
		BMI = BMIs ranged from 21.6 to 23.2 kg/m^2^	A randomized two-way crossover design to assess the effect of the prior consumption of fat either a high-caloric high-fat-diet meal (HFM) or noncaloric water load (WL) on the BOLD response to the oral control (OC) and fat stimuli (FS)	
			Visual analog scale	
			CCK measurement	
			MRI scanning	
			Shapiro-Wilk normality test	
Frank-Podlech et al. (58), 2019, Germany	*N* = 15 healthy adult males	Age = 24.6 years ± 2.4 SD	Visual analog scale (VAS)	Oral fat sensitivity was positively correlated with functional connectivity between homeostatic regions and limbic areas in the high-fat condition but negatively correlated with functional connectivity between the dorsal striatum and somatosensory regions in the low-fat condition.
		Sex = 15 adult males	Functional MRI (fMRI)	
		BMI = 23.1 kg/m^2^ ± 2.0 SD	Seedbased functional connectivity (FC) maps	
Grabenhorst et al. (59), 2014, United Kingdom	*N* = 14 Adults	Age = mean age of 24 years	Fat flavor stimuli consisted of vanilla and strawberry-flavored dairy drinks delivered through Teflon tubes and held between the lips during fMRI	The activity in somatosensory cortex (SSC) was more strongly correlated with the orbitofrontal cortex (OFC) during the consumption of a high-fat food with a pleasant (vanilla) flavor compared to a low-fat food with the same flavor.
		Sex = 5 females, 9 males	fMRI data acquisition and analysis	
		BMI = not specified	Psychophysiological interaction (PPI) for differential FC	
Graham et al. (60), 2021, United Kingdom	*N* = 48 adults	Age = 32.7 ± 11.4 years	Fat taste sensitivity assessment	The BDNF rs6265 SNP and the TNNI3K rs1514175 SNP were analyzed to find differences in fat taste sensitivity in an ethnically similar cohort. The rs6265 SNP was found to have lower fat taste threshold for the CT/TT genotype compared to the CC genotype. The rs1514175 SNP AA/AG genotype was found to have a moderately higher fat taste threshold.
		Sex = all females	Anthropomorphic measurements	
			SNP genotyping	
Han et al. (61), 2020, Germany	*N* = 38 adults	Age = mean 25.9 years	Sweet taste sensitivity test	High sensitivity was associated with: higher preference for carbs, higher liking of sweet foods, lower liking for protein-dominated foods, higher level of frontal inferior operculum activity in response to sweet vs. savory food odors, and stronger insular activations to high-fat vs. low-fat food odors. Additionally, individual sweetness sensitivity was positively correlated with insula activation in response to a high-fat odorant.
		Sex = 21 females, 17 males	Macronutrient and taste preference task	
		BMI = 18.5–29.4	fMRI study with odor stimuli	
			Macronutrient and energy density questionnaire	
Kadouh et al. (62), 2019, USA	*N* = 40 liraglutide (*n* = 19) and Placebo (*n* = 21) obese American adults	Age = mean age of 42 years for liraglutide and 37 years for placebo	Standardized nutrient drink test	Compared to placebo group, liraglutide group had significant reductions in MTV; prospective food consumption score; desire to eat something sweet, salty, savory, or fatty; and an increase in perceived fullness. Postprandial plasma levels of GLP-1 decreased and PYY levels increased with liraglutide relative to baseline. Significant reductions in total body, trunk, and upper and lower body fat without reduction in lean body mass were observed.
		Sex = all females	VAS	
		BMI = BMIs ranged from a baseline BMI of 37.2 kg/m^2^ for liraglutide and 34.6 kg/m^2^ for the placebo group.	Plasma gastrointestinal hormone measurement	
Karmous et al. (24), 2018, Tunisia	*N* = 104 NW (*n* = 52) and OB (*n* = 52) Tunisian adults	Age = normal weight (NW) mean age 35.3 ± 4.10 years and OB mean age 35.0 ± 5.43 years	Taste preference test for linoleic acid (LA)	There was a positive correlation between BMI and PROP oral detection thresholds in obese participants. There was no association between rs10246939 and obesity.
			3-AFC method	
		Sex = NW 29 females, 23 males and OB 42 females, 10 males.	Venous blood collection	
			Column chromatography	
		BMI = BMIs ranged from 21.78 to 24.66 kg/m^2^ (NW) and from 28.98 to 39.6 kg/m^2^ (OB)	ELISA	
			Genomic DNA extraction RFLP method and gel electrophoresis: analyze rs10246939 (Val296Ile) and rs1726866 (Ala262Val)	
Karthi et al. (63), 2021, India	*N* = 444 adults	Age = not specified	LA oral detection threshold test	The AA genotype at rs1761667 (of CD36 gene) had a higher LA detection threshold (lower sensitivity to fat taste).
		Sex = 234 males, 210 females	Genotyping for genetic polymorphisms	
		BMI = group 1 (NW) ranged 20–24.9 kg/m^2^, group 2 (OW) ranged 25–29.9 kg/m^2^, and group 3 (obese) ranged 30–35 kg/m^2^	Measurement of PYY hormone	
Kulkarni and Mattes (64), 2014, USA	*N* = 15 healthy adults	Age = range: 18–50 years	Salivary nonsteroidal fatty acids (NEFA) measures	Lingual lipase was active during oral processing of almond and coconut. No activity of lingual lipase was detected during processing of almond butter. There was only weak evidence lingual lipase is a determinant of oral fat detection. Lingual lipase may only contribute to NEFA generation and oral fat detection.
		Sex = 11 females, 4 males	Sensory ratings for almond butter with and without lipase inhibitor oralist	
		BMI = mean: 18.5–25 kg/m^2^	NEFA present in 5 HF foods varying in physical states and fatty acid composition (almond, almond butter, olive oil, walnut, and coconut)	
Liu et al. (65), 2018, Australia	*N* = 36	Cohort 1 and 2	Fungiform papillae collection	qRT-PCR and Western blotting indicated that mRNA and protein of CD36, FFAR4, FFAR2, GPR84, and delayed rectifying K+ channels are expressed in human fungiform taste buds. The expression level of CD36 was associated with the liking difference score between high-fat and low-fat food.
		Age = between 20 and 42 years	qPCR	
		Sex = 6 females, 4 males	Phenotyping test	
		BMI = between 20 and 33 kg/m^2^	Western blots	
		Cohort 3	Immunohistochemistry	
		Age = 8 pairs of female twins		
		Sex = age ranged between 20 and 62 years		
		BMI = between 17 and 35 kg/m^2^		
Méjean et al. (66), 2015, France	*N* = 216 French adults	Age = mean age 49.6 ± 13.5 years	Questionnaires: dietary intake, physical activity, anthropometric measures, lifestyle, and socioeconomic and health status	Salivary flow was positively associated with liking for fat. Proteolysis was positively associated with liking for saltiness and for fat.
		Sex = males and females	Sensory tests: liking for salty, sweet, and fat sensations	
		BMI = not specified	Saliva for: protein concentration, enzyme activity, lipolysis, proteolysis, amylolysis, Carbonic anhydrase 6 and cystatin SN and sodium quantification, and antioxidant capacity	
			24-h food records	
			Food products assessment for sensory liking	
Melis et al. (67), 2015, Italy	*N* = 64 Caucasian adults	Age = mean age 27.6 years	PROP taster status (super taster, medium taster, or nontaster)	Subjects homozygous for GG of the rs1761667 polymorphism showed higher sensitivity to oleic acid than AA subjects. The capability to detect oleic acid was directly associated with TAS2R38 or PROP responsiveness. PROP nontasters had a lower papilla density than tasters, and those with genotype GG of the rs1761667 polymorphism had lower oleic acid thresholds than PROP nontasters with genotype AA.
		Sex = 41 females, 23 males	LMS	
		BMI = BMIs ranged from 18.6 to 25.3 kg/m^2^	Oral perception (for fatty acid) using 3-AFC procedure	
			DNA extraction and PCR for CD36 SNPs genotyping = rs1761667 (G/A) and rs1527483 (C/T)	
			TAS2R38 genotyping for three SNPs at basepairs 145 (C/G), 785 (C/T), and 886 (G/A) were performed using PCR	
Melis et al. (68), 2017, Italy	*N* = 126 Caucasian volunteers from Italy	Age = not specified	Blood samples and saliva collection for DNA extraction	The A/G allele of the rs1761667 polymorphism of *CD36* was associated with distinct metabolic patterns in NW and obese subjects. The G allele of the CD36 gene rs1761667 was associated with increased endocannabinoid plasma levels and a trend for increased waist/hip ratio in obese subjects, even though exhibited decreased BMI with respect to those with AA genotype.
		Sex = 80 females, 46 males	HPLC analysis: aliquot of the lipid fraction, from erythrocytes	
		BMI = 64 participants with BMIs ranging from 18 to 25 kg/m^2^ and 62 obese participants with BMIs 30–50 kg/m^2^	SAFA were analyzed as fatty acid methyl esters by a gas chromatography with FID	
			Fisher method: genotype distribution and allele frequencies of CD36 SNP	
Melis et al. (69), 2018, Italy	*N* = 46 Caucasian adults	Age = mean age 26.6 ± 0.79 years	PROP-taster status classification: LMS	rs1761667 in CD36 indicate that the effectiveness of l-Arg supplementation in increasing the perception of oleic acid is directly related to the presence of allele A in rs1761667 polymorphism of CD36 gene, which has been associated with a lower expression of CD36 scavenger receptor, with respect to that of allele G.
		Sex = 38 females, 8 males	Saliva samples: DNA extraction	
		BMI = BMIs ranged from 18.6 to 25.3 kg/m^2^	rs713598, rs1726866, and rs10246939 genotyping of TAS2R38 locus, rs1761667 of CD36 and rs2590498 of OBPIIa gene polymorphism	
			PCR	
			DFT method LA oral perception with l-Arg	
Mounayar et al. (70), 2014, France	*N* = 73 French adults	Age = mean age of 43 ± 15.4 years, for sensitive+ subjects and 40 ± 13.1 years for sensitive− subjects	The screening procedure to determine fatty acid sensitivity. Low concentration of C18:1 was considered ‘‘sensitive+,’’ while subjects who detected least frequently the sample containing the high concentration of C18:1 were considered ‘‘sensitive−’’	Fatty acid sensitivity was associated with changes in saliva composition induced by C18:1 stimulation.
		Sex = 73 males	Saliva collection	
		BMI = BMIs ranged from 22.3 to 26.7 kg/m^2^ for “sensitive+” subjects,and 21.6 to 27.8 kg/m^2^ for “sensitive−” subjects	Protein content measurement and two-dimensional electrophoresis analysis	
			^1^H-NMR analysis	
Mrizak et al. (71), 2015, Tunisia	*N* = 203 Tunisian adults	Age = mean age 38.4 ± 11.4 years	Plasma and serum from fasting venous blood samples were collected	The A allele of cluster of differentiation 36 (CD36) SNP 1761667 is associated with decreased lipid taste perception in obese Tunisian women. Women with the CD36 GG genotype exhibited oral detection thresholds for oleic acid that were more than three times lower than those with the CD36 AA genotype.
			Enzymatic methods were used to determine blood parameters (serum TAG and total and free cholesterol concentrations)	
		Sex = 203 females	Taste emulsions containing food grade oleic acid (Sigma) were prepared	
			3-AFC	
		BMI = BMIs ranged from 30.4 to 38.8 kg/m^2^	Genomic DNA extraction	
			PCR amplification	
Ong et al. (72), 2017, Malaysia	*N* = 313 Chinese (n = 293) and Indian (n = 20) adults	Age = mean age 20.73 ± 1.55 (males) and 20.74 ± 1.49 (females) years	Sensory stimuli and rating test	CD36 rs1761667 and rs1527483 are not associated with obesity and adiposity. CD36 rs1527483 plays a role in OFP.
		Sex = 195 females, 118 males	Participants were presented with four increasing oil (fat) content by-weight custards and low-fat/regular versions of commercially available milk, mayonnaise, and cream crackers.	
		BMI = 88% of participants had a BMI ≥25 kg/m^2^, 12% had a BMI <25 kg/m^2^	VAS	
			DNA extraction	
			PCR-RFLP-CD36 rs1761667 and rs1527483 SNPs genotype	
Plesník et al. (73), 2018, Czech Republic	*N* = 116 young Caucasian adults	Age = mean age 21.84 ± 0.22 years	Food craving inventory (FCI)	Participants with the CC genotype of the rs1527483 polymorphism of the CD36 gene had lower BMI, waist circumference, waist:height ratio, and higher sensitivity for LA than the participants with the CT and TT genotypes. No association was found between the rs32TLCA18 polymorphism and LA detection threshold or BMI, waist circumference, and waist:height ratio.
		Sex = 73 females, 43 males	Oral LA detection thresholds	
		BMI = BMIs ranged from 22.92 to 23.76 kg/m^2^	Genomic DNA extraction	
Proserpio et al. (74), 2016, Italy	*N* = 103 adults	Age = less than 65 years of age (not specified)	Taste threshold OA was used to elicit fat sensation tastes, 7 concentrations of each compound prepared 3-AFC test	Obese subjects had higher threshold values (for fat, sweet, bitter, salty, and sour tastants) and a reduced number of fungiform papillae. Obese subjects had significantly higher liking ratings for high energy dense foods. Food neophobia was not associated with BMI or taste sensitivity.
		Sex = 28 obese females, 23 obese males; 27 normal weight females, 25 normal weight males	Fungiform papillae density	
		BMI = 27.76 ± 7.10	FNS	
			Food liking: 26-item liking questionnaire	
Ramos-Lopez et al. (75), 2019, Spain	*N* = 474 Spanish and Chilean adults	Age = mean age 47.2 ± 14.1 years	Nutriepigenomic analysis	15 CpG were correlated with BMI (FDR adjusted-linear regression). No relationships between methylation status of olfactory genes and metabolic markers and blood pressure. Pathway enrichment analysis revealed a significant contribution of genes involved in the regulation of the olfactory transduction network, such as odor detection and signal processing in the nervous system. These genes included the olfactory receptors OR4D2, OR51A7, OR2T34, and OR2Y1 and several downstream effectors, such as SLC8A1, ANO2, PDE2A, CALML3, GNG7, CALML6, PRKG1, and CAMK2D.
		Sex = 303 females, 171 males	Associations between taste receptors polymorphisms, dietary intakes, lipid disorders, and liver disease in Mexican subjects were reported	
		BMI = BMIs ranged from 24.5 to 35.7 kg/m^2^	Data normality was screened by the Kolmogorov-Smirnov test.	
Running et al. (76), 2017, USA	*N* = 78 adults for Stearic acid test	Age = range 18–55 years	Three fatty acids with different degrees of saturation were tested (stearic, oleic, and linoleic acid)	This study demonstrates that degree of unsaturation influences rejection of a chocolate with added FFA. With polyunsaturated FAs being rejected by both taste and aroma at lower concentrations than the monounsaturated (oleic) acid. No rejection observed for the flavor of saturated fatty acids.
	*N* = 69 adults for oleic acid test	Sex = stearic acid test: 57 females, 21 males; for oleic acid test: 48 females, 21 males; linoleic acid test: 57 females, 18 males	Paired preference tests were conducted for 10 concentrations (0.04% to 2.25%) of added FFAs compared with the control chocolate without added FFAs.	
	*N *= 75 linoleic acid test	BMI = not specified	Stearic acid was tested for flavor (tasting and nares open), whereas the unsaturated fatty acids were tested for both aroma (orthonasal only and no tasting) and taste (tasting with nares blocked to eliminate retro nasal odor)	
Shen et al. (77), 2017, United Kingdom	*N* = 136 UK adults	Age = 65% of participants were 18–29, 35% 30–55 years of age; range 18–55 years	Two visits 2 weeks apart. First visit: prestudy questionnaires, buccal cell swab, anthropometric measurements, and FPD measurements. Between visits 1 and 2 the FFQ and TFEQ were conducted. Second visit: fat liking rating and PROP sensitivity test.	Liking for ice cream was significantly affected by the fat content of the sample, and by demographic factors (gender, ethnicity, age) but no associations were found between CD36 rs1761667 or CA6 rs2274333 genotypes, PROP taster status, nor FPD.
			LMS-PROP taster status	
			Blue dye: FPD	
		Sex = 95 females, 41 males	DNA extraction from buccal cells	
			Trained sensory panel: for sensory profile	
			Nine-point hedonic category scale-fat liking	
		BMI = 17–43.5 kg/m^2^, mean of 22.9 ± 0.34 kg/m^2^; 74% of participants were in the normal weight range (18.5–25 kg/m^2^)	Oxford FFQ: participants diet assessment	
			TFEQ: to categorize eating behavior	
			Tanita body composition analyzer: for BMI	
Solakivi et al. (78), 2015, Finland	*N* = 736 Finnish adults (314 with hypertension and 422 controls)	Age = 50 years (50-year-old cohort of TAMARISK study)	Interview-structured questionnaire about health and health-related behavior	CD36 rs1761667 was associated with BMI in the TAMRISK study. Considering the multitude of roles of CD36 in processes related to fatty acid metabolism and sensing in the body, it is plausible that genetic variation in human fatty acid transporter CD36 can have effects on regulation of energy homeostasis.
		Sex = 129 females with hypertension; 185 males with hypertension, 160 females and 262 males in control group	Buccal swabs- DNA extraction	
		BMI = 28.8 ± 5.2 (hypertension group) and 25.5 ± 3.6 (control group)	Genotyping: CD36 SNP rs1761667	
			Physical examination	
			Serum cholesterol and glucose	
Sun et al. (79), 2016, USA	*N* = 33 right-handed American subjects	Age = mean = 26.9; range 18–40	gLMS used for internal state ratings and perceptual quality ratings	These findings demonstrate that the effect of a meal on suprathreshold odor intensity perception is associated with metabolic measures such as body weight and total ghrelin reactivity, supporting endocrine influences on olfactory perception.
		Sex = 17 females, 16 males	Plasma levels of FFAs, triglycerides, ghrelin, insulin	
		BMI = 13/33 had BMI >25.0	fMRI with MRI compatible olfactometer and portable gustometer	
Watanabe et al. (80), 2019, Japan	*N* = 53 young Japanese adults	Age = mean age 24.3 ± 1.5 years; range, 20–38 years	Genotype Trp64 (Trp64trp and Trp63Arg)	When divided into two groups based on greasy food preference, the Trp64Arg had higher preference for HF foods (vs. Trp64Trp), suggesting that Arg substitution might genetically enhance HF preference. Understanding the relationship between *ADRB3* Trp64Arg substitution and fat preference could be valuable for obesity prevention.
		Sex = 28 females, 25 males	Buccal swab: DNA extraction	
		BMI = BMIs ranged from 17.1 to 28.2 kg/m^2^		
Voigt et al. (41), 2014, Germany	*N* = 12 trained adult participants	Age = 26−40 years	Oral fatty acid sensitivity test (using oleic acid)	Lipases (LIPs), different from LIPF (as observed in rodents), are present in human salivary glands. Oral perception of triglycerides is associated with differential LIP activities on individual threshold concentrations.
		Sex = 4 males 8 females	Oral lipolytic activity	
		BMI = not specified	Hyman circumvallate papillae biopsy for RT-PCR and in situ hybridization	
			In vitro lipolysis	
Zhou et al. (81), 2021, United Kingdom	*N* = 94 health adults	Age = range 18−70 years	Fatty acid taste sensitivity test	Higher fungiform papillae density was correlated with higher fat taste sensitivity. Authors hypothesize that this is due to a larger amount of fatty acid taste receptors on the tongue.
		Sex = 64 females, 30 males	Measure of fungiform papillae density	
		BMI = mean 22.7 kg/m^2^ female, mean 24.1 kg/m^2^ male	Tactile sensitivity measurement	
			Biscuit mouthfeel perception and texture measurements	

AFC, alternative-forced choice; BMI, body mass index; BOLD, blood-oxygen-level-dependent; DFT, density functional theory; HEX, ethyl hexanoate; but, ethyl butanoate; FT, fat taste; FAT, fatty acid taste; FID, flame ionization detector; FFQ, food frequency questionnaire; FCI, food craving inventory; FNS, food neophobia scale; fMRI, functional magnetic resonance imaging; FPD, fungiform papillae density; GLP-1, glucagon-like-peptide-1; HF, high fat; HPLC, high performance liquid chromatography; LMS/gLMS, labeled magnitude/general labeled magnitude scales; LF, low fat; OA, oleic acid; ^1^H-NMR, proton nuclear magnetic resonance; MTV, maximal tolerated volume; PPI, psychophysiological interaction; PROP, 6-*n*-propylthiouracil; RFLP, restriction fragment-length polymorphism; qRT-PCR, quantitative real-time-polymerase chain reaction; SAFA, saturated fatty acid; SNP, single-nucleotide polymorphism; TFEQ, three-factor eating questionnaire; TC, total cholesterol; TG, triglycerides; CCK, cholecystokinin; VAS, visual analog scales; NEFA, nonesterified fatty acids; CD36, cluster of differentiation 36.

**Table 2.** Preclinical study characteristics and findings

Author (Reference), Year, Country	Sample Size	Animal Characteristics	Design/Methods	Key Findings
Ackroff and Sclafani (82), 2014, USA	*N* = 43 C57BL/6J (B6) *Mus musculus* (mice)	Age = 10 weeks old	Intralipid intragastric infusion	Intragastric (IG) administered self-infusions of fat produces concentration-dependent increases in the intake of and preference for a flavored solution in C57BL/6J mice. IG fat rapidly generates concentration dependent postoral signals that stimulate intake and enhance preferences for energy-dense foods.
		Sex = all male	Licking tests	
		Groups = infusions of 1.6% (*n* = 11), 3.2% (*n* = 10), 6.4% (*n* = 11), or 12.8% (*n* = 11) intralipid	Two-bottle preference tests	
Ahn et al. (83), 2017, USA	*N* = 22–124 *Drosophila melanogaster* (fruit fly)	Age = 3–7 days	Proboscis extension reflex (PER) assay	A novel role for IR25a and IR76b in fatty acid taste was established. These two subunits are not only critically important to elicit PER responses in flies when challenged with fatty acids but are also necessary for fatty acid induced Ca^2+^ increases in tarsal sweet gustatory receptor neurons (GRNs).
		Sex = all female flies	Immunofluorescence	
		Groups = control, *IR25a*, and *IR76b* mutant	Calcium imaging	
Ancel et al. (84), 2015, France	*N* = 10–12 C57Bl/6 mice	Age = young mice	Two-bottle preference tests	GPR120 disruption is not associated with fat preference or CD36 expression in circumvallate papillae. However, GPR120 agonist, grifolic acid, triggered a rise in [Ca^2+^]_i_ which was drastically decreased when GPR120 was disrupted. These data suggest that although GPR120 is expressed in lingual tissue, it is not required for oral fat detection.
		Sex = all male	Licking tests	
		Groups = wild type (WT) and GPR120^−/−^	Conditioned taste aversion tests	
			Measurement of Ca^2+^-signaling in taste bud cells (TBC)s	
Avalos et al. (85), 2020, USA	*N* = 5–8 (per group) C57BL/6Tac male mice and CB_1_R-deficient mice	Age = 8–10 weeks	Western diet preference test	Preference for the Western high-fat diet (HFD) was significantly decreased after pharmacological blockade of CB_1_R. HFD preference was also decreased for CB_1_R^−/−^ mice, showing that CB_1_ in the intestinal epithelium plays a role in fat preference.
		Sex = all male	Pharmacological blockade of cannabinoid receptor type 1 (CB_1_R)	
		Groups = WT and CB_1_R^−/−^	Utilizing CB_1_R^−/−^ mice	
Avau et al. (86), 2015, Belgium	*N* = 32 C57BL/6J mice	Age = adult mice	Comparing WT with knockout mice on HFD	α-Gustducin knockout mice did not gain as much weight as WT mice on HFD. Intragastric administration of bitter agonists caused further weight loss via α-gustducin pathway. Therefore, α-gustducin is involved in induction of obesity during HFD.
		Sex = not specified	Comparing food intake after administration of bitter tastants vs. control	
		Groups = WT and α-gustducin^−/−^	Respiratory quotient/heat production measurements	
Bensalem et al. (87), 2020, France	*N* = 24 C57B1/6 mice	Age = 12- to 14-week-old mice	Comparing WT with knockout mice on standard/HFD	The TGR5−/− obese mice exhibited high daily food/energy intake, fat mass and inflammatory status. TGR5−/− obese mice maintained an attraction for lipids. In TBCs, the fatty acid-triggered Ca^2+^ signaling was increased in TBC from TGR5−/− obese mice. TGR5 may modulate fat eating behavior and obesity.
			Analysis of lean/fat mass	
		Sex = all male mice	Two-bottle preference test	
			LPS assay	
		Groups = WT and TRG^−/−^; standard chow and high-fat-chow mice	Taste bud isolation from CV	
			Measurement Ca^2+^ signaling	
Boone et al. (88), 2021, USA	*N* = 5–22 (per group) C57BL/6J mice	Age = >8 weeks	Olfactory bulb ablation and anosmia screening	Anosmic mice (following complete removal of the olfactory bulb) and sham mice (with intact olfactory bulbs) both displayed comparable HFD intake. HFD smell (in the absence of consumption) did not alter feeding or devaluation of standard food. Thus, while the olfactory bulb may play a role in fat-olfaction it may not be necessary for the development a HFD preferential consumption.
		Sex = both: male and female	Inaccessible and short accessibility food experiments	
		Groups = standard diet (SD), SD + HFD, and SD + inaccessible HFD	Fast-refeed tests	
			Measuring SD and HFD consumption of control and anosmic mice	
Braymer et al. (89), 2017, USA	*N* = 5–9 (per group) obesity-prone (OP) and obesity-resistant (OR) S5B/P1 rats	Age = 8–9 weeks old	Linoleic acid (LA) preference testing	Lingual CD36 mRNA levels increased in OR rats, but not in OP rats. Lingual application of CD36 siRNA decreased LA preference in OR rats, but not in OP rats. OP rats did not show other effects of CD36 siRNA on HFD preference or HFD or LFD intake.
		Sex = all male	Administration of lingual CD36 siRNA	
		Experiment 1: effect of fasting- 16-h fast (OP *n* = 8; OR *n* = 7) + standard chow (OP *n* = 8; OR *n* = 8)	Effect of fasting: 16-h fast + chow	
		Experiment 2: effect of HFD on LA preference (OP *n* = 5; OR *n* = 5)	Effect of HFD: HFD vs. chow fed	
		Experiment 3: effect of fasting lingual CD36 mRNA expression + standard chow (OP *n* = 6; OR *n* = 6), fasted overnight (OP *n* = 9; OR *n* = 6), refed for 2 h (OP *n* = 7, OP *n* = 7)		
Brown et al. (90), 2021, USA	*N* = 13–41 (per trial) IR56d *Drosophila melanogaster*	Age = 7–9 days	Aversive taste memory	Flies were able to discriminate medium-chain fatty acids (MCFAs) from short-chain fatty acids (SCFAs) and long-chain fatty acids (LCFAs). They were not able to discriminate different MCFAs from each other. Similar discrimination abilities were exhibited in both males and females.
		Sex = all female		
Buttigieg et al. (91), 2014, Chile	*N* = 5–12 (per group) Swiss CD1 mice	Age = weaned mice	RD vs. HFD chow preference tests	After 18 days (short term) exposure to HFD, mice developed preference for high fat chow, indicating that high fat preference is not spontaneous in CD1 mice, but can be acquired after short term exposure to HFD. Development of preference for HFD dependent on NMDA receptor signaling.
		Sex = all male	HF preference tests after 18-day exposure to either RD or HFD	
		Groups = Exp1 group (*n* = 12); Exp2: regular diet (*n* = 9) and high-fat diet (*n* = 9); Exp3: IP injections of ketamine, ifenprodilMK-801, or PBS (control), (*n* = 5 per group)	NMDA antagonist injections during HFD with preference tests	
Calder et al. (92), 2021, USA	*N* = 66 C57BL/6J WT and Growth Hormone Secretagogue Receptor knockout (*Ghsr -/-)* mice	Age = 6 weeks old	Conditioned taste aversion assay	GHSR expression within the taste system- with GHSR being largely present in type II cells. Additionally, HFD-fed female GHSR-/- exhibited reduced responsiveness to LA (compared to WT). Ghrelin signaling may play a critical role in the recognition of fatty acids in female mice, and this may contribute to ingestive behaviors.
		Sex = both: female (*n* = 33) and male (*n* = 34)		
		Groups = WT (*n* = 37) and *Ghsr-/-* (*n* = 29)		
Camandola and Mattson (93), 2017, USA	*N* = 5–19 mice per group	Age = adult mice	Two-bottle preference test	TLR4 knockout mice show low preference for fat. TRPM5 and G-protein dependent phospholipase Cb2 significantly decreased in TLR4 knockout. Overall, TLR4 promotes fat ingestion (FA endocytosis) and preference for fat intake.
		Sex = all male	Comparing standard diet and obese diet	
		Groups =WT and TLR4 knockout mice; standard diet and high-fat, high-sugar diet	Tongue epithelium PCR	
De la Cruz et al. (94), 2015, USA	*N* = 37 Sprague-Dawley rats (260−300 g)	Weight = 260- to 300-g rats	Feed rats test solutions	Corn oil caused dopaminergic signaling, measured by c-Fos-like immunoreactivity, in the ventral tegmental area, infralimbic/prelimbic prefrontal cortex, dorsal striatum, nucleus accumbens core, and basolateral/central-cortico-medial amygdala. This suggests that these brain regions may form a distributed network to help mediate fat intake.
		Sex = all male	Brain tissue collection	Consumption of corn oil solutions, isocaloric to glucose and fructose, significantly increased FLI in all sites except for the NAc shell.
		Groups = water (*n* = 7), cherry-flavored saccharin (0.2%) (*n* = 7), corn oil in xanthan gum (3.5%) (*n* = 5), fructose (8%) (*n* = 7), glucose (8%) (*n* = 7), and saccharin, xanthan gum (0.3%) (*n* = 4)	Immunoreactive c-Fos quantification	
Devineni et al. (95), 2019, USA	*N* = 6–18 (per trial group/set) *Drosophila melanogaster*	Age = 3–6 days old	PER experiments were conducted by taste stimulation of the labellum	Fed flies show taste aversion to acetic acid, whereas starved flies show a robust appetitive response. These opposing responses are mediated by two different classes of taste neurons, the sugar- and bitter-sensing neurons.
		Sex = mated all females	Taste stimuli	
		Groups = Gr64f-Gal4; Gr66a-Gal4; ppk28-Gal4; Gr98d-Gal4, Gr22f-Gal4, Gr59c-Gal4, and Gr47a-Gal4; UAS-Kir2.1; UAS-GCaMP6f, UAS-norpA^RNAi,^, poxnΔ^M22-B5^ and poxn Δ^M22-B5^+ SuperA rescue; Δ8Grs (R1, ΔGr5a; ΔGr61a, ΔGr64a-f) and Δ8Grs with transgenes for GCaMP imaging (R1, ΔGr5a; Gr61a-Gal4, UAS-GCaMP6m; ΔGr61a, ΔGr64a-f); IR25a^1^ and IR25a^2^; IR76b^1^ and IR76b^2^	Surgery was performed prior to starvation, and after surgery flies were given∼30 min to recover in food vials before starvation	
			Calcium imaging	
Djeziri et al. (96), 2018, France	*N* = 6 (per group) C57B1/6J mice	Age = 6–10 weeks	Two-bottle preference test	HFD mice showed decreased CD36 expression. OLA-treated obese mice showed increased CD36 mRNA in TBC. They exhibited higher preference for fat and more sensitive orosensory detection of OLA. Oleic acid triggered an increase in intracellular calcium in mTBCs. Oleic acid-induced increases in Ca^2+^ were abolished completely in the presence of SSO, a selective CD36 inhibitor.
		Sex = all female	Two-bottle preference test	
		Groups: WT (*n* = 6), HFD (*n* = 6), HFD + OLA (*n* = 6)	Blood glucose tolerance test	
			Plasma LPS, insulin, liver lipids analyses	
			Fatty acid analysis	
			Measurement of Ca^2+^ signaling	
			Isolation of mTBCs	
Espitia-Bautista and Escobar (97), 2019, Mexico	*N* = 80 (10 per group) Wistar rats	Age = not specified	Assessment of binge-type eating, food anticipatory activity, and effort behavior to obtain the diet.	After an acute exposition, rats ate more SRD than FRD, but FDR stimulated higher c-Fos. After chronic administration, the FDR group exhibited higher levels of BTE and FAA; this was associated with higher c-Fos and accumulation of ΔFosB in the corticolimbic system.
		Sex = all male	Immunohistochemistry	
		Groups = (*n* = 10)		
		Experiment 1: sugar-rich diet (SRD) and fat-rich diet (FRD) 8 groups: SRD = 10%, 25%, 50%, 75% FRD = 10% 25%, 50%, 75%		
		Experiment 2: 3 groups: chow; 50% SRD, 50% FRD rats		
		Experiment 3: groups: chow, Daily 1 h restricted access to 50% SRD, or to 50% FRD		
		Experiment 4: 3 groups: chow; 50% SRD; 50% FRD		
Fardone et al. (98), 2019, USA	*N* = 72 M72-IRES-tauGFP mice with mixed agouti/C57BL6/J	Age = not specified	Odor (Olfr160 ligand) exposure	Neuronal excitability of juxtaglomerular (JG) cells is significantly reduced in moderate HFD (MHF) and HFD mice when stimulated by a preferred odorant, whereas control animals showed normal activation. This is mainly seen in interneurons surrounding the lateral but not medial glomerulus. Diet-induced obesity (DIO) causes deleterious effects on OSN survival that extends to a reduced neuronal activity of JG cells surrounding the genetically identified glomerulus for that class of ORs.
		Sex = all male	c-Fos immediate-early gene expression for neuronal activity mapping	
		Groups = control food (*n* = 24), moderately high-fat diet (*n* = 19), high-fat diet (*n* = 29)	IPGTT	
Gaudet et al. (99), 2019, USA	*N* = 39 Sprague-Dawley rats	Age = 8–10 weeks	Comparing expression levels of taste-related genes between standard chow diet vs. continuous, daily, and intermittent HFD access	Expression levels of the fat taste-sensing markers, CD36, SERT, and TPH2 mRNA in the circumvallate papillae were higher in the continuous HFD group.
		Sex = all male		
		Groups = chow diet (*n* = 14), continuous HFD (*n* = 9), daily (*n* = 8), intermittent (*n* = 8)		
Olvera Hernández et al. (100), 2021, France	*N* = 48 Wistar rats	Age = 3 months	Evaluation of preferences for fatty and sugary foods	Adult males and females born to undernourished dams exhibited increased expression of *Cd36*, *Trpm5*, *Plc-b2* in the hypothalamus. The severity was greater in females. Only males from undernourished dams consumed more standard and sweetened food and had higher AgRP NPY in hypothalamus and increased dopamine transporter and dopamine receptor d2 in VTA.
		Sex = both: female (*n* = 24) and male (*n* = 24)	Tissue collection from the tongue, nucleus accumbens, and ventral tegmental area (VTA) in the brain	
Iskhakov et al. (101), 2019, USA	*N* = 30 inbred BALB/c, C57BL/6 and SWR mice	Age = 6 weeks	Scopolamine injections and comparing intralipid intake	Scopolamine (muscarinic receptor antagonist) reduced fat intake in all 3 strains and eliminated the ability to learn fat-CFP in the 3 strains. Therefore, muscarinic receptor signaling mediates learning and to a lesser degree maintenance of fat-CFP while maximally inhibiting fat intake in the 3 strains.
		Sex = all male	Fat-conditioned flavor preference (CFP) tests	
		Groups = (*n* = 10 per group) BALB/c, C57BL/6, and SWR		
Jung et al. (102), 2018, Korea	*N* = 20 Canton-S wild-type *Drosophila melanogaster*	Age = adult flies	Life span assays	A HFD reduced *DmOrco* gene expression by 70% in olfactory neurons and decreased olfactory sensitivity to short-chain fatty-acids. This suggests HFD leads to olfactory dysfunction in homeostatic processing in *Drosophila*.
		Sex = all male	Climbing assays	
		Groups = (*n* = 20 per vial on HFD and SD)	Odor stimulation	
			Behavioral assay	
			Electrophysiological readings	
Khan et al. (103), 2017, France	*N* = 7 per group C57BL/6J WT and Erk1-/- mice	Age = <9 weeks	Comparing standard diet vs. HFD	Erk1-/- exhibited a low preference for dietary fatty acids and developed obesity. They also showed higher phosphorylation of MEK, an upstream regulator of ERK1/2 and exhibited high ERK2 phosphorylation, high lipogenesis, and low fatty acid oxidation. Overall, ERK1 and ERK2 have different but key roles in obesity.
		Sex = all male	OGTT	
		Groups = (*n* = 7 per group) WT-normal diet (ND), Erk1-/- ND, WT-HFD, Erk1-/- HFD	Lipid analysis	
			Western blots	
			mRNA RT-qPCR	
Kim et al. (104), 2018, Korea	*N* = 3–25 *Drosophila melanogaster* per trial group	Age = 3–5 days old	CRISPR/Cas9 *Gr64* cluster deletion	*Gr64e*, a gustatory receptor, is required for the behavioral and electrophysiological responses to fatty acid detection. It functions as an ion-gated ligand channel for glycerol detection and acts downstream of phospholipase C signaling.
		Sex = both: mated males and females	Proboscis extension reflex assay	
		Groups = multiple Gr64e mutant groups		
Kraft et al. (105), 2017, USA	*N* = 35 BALB/c and SWR mice	Age = 6 weeks old	Two-bottle conditioned stimuli (CS) choice test	Preference response for flavor associated with higher intralipid content was eliminated in mice treated with 100ug/kg MK-801. Therefore, NMDA receptor signaling must be needed in the formation of major triggers toward fat preference learning.
		Sex = all male	Systemic NMDA antagonist (MK-801) injections	
		Groups = vehicle control (BALB/c, *n* = 8; SWR, *n* = 9) and MK-801 (BALB/c, *n* = 9; SWR, *n* = 9)		
Lacroix et al. (106), 2015, France	*N* = 19 OR and OP Sprague-Dawley rats	Age = 4 weeks old	Weight recorded	In OP rats, *1*) decreased odor threshold, but *2*) poor olfactory performances, associated with learning/memory deficits, *3*) decreased influence of fasting, and *4*) impaired insulin control on food-seeking behavior were reported. Modulation of metabolism-related factors implicated in *1*) electrical olfactory signal regulation (insulin receptor), *2*) cellular dynamics (glucocorticoids receptors, pro- and antiapoptotic factors), and *3*) homeostasis of the olfactory mucosa and bulb (monocarboxylate and glucose transporters).
			Food intakes were recorded during the diurnal and nocturnal phases of the day	
			Insulin tolerance test	
		Sex = all male	Concentrations of glucose, triglycerides, insulin, and leptin were measured	
			Tea-ball (odor) test	
			Conditioned odor aversion test	
		Groups = OR (*n* = 9) and OP (*n* = 10)	Hidden cookie test	
			Western blot analysis	
			Quantitative real-time RT-PCR	
Lee et al. (107), 2015, Japan	*N* = (not specified) C57BL6/J WT and CD36-knockout mice	Age = 8–12 weeks old	Two-bottle choice test	WT mice avoided solutions with KOdiA-PC (CD36 ligand), an irritant phospholipid species CD36. Knockout effects are only seen at low levels of KOdiA-PC, suggesting that CD36 contributes to lipid recognition, but may not be the sole receptor. Mice that had olfactory nerve transected could not perceive KOdiA-PC. This implies that CD36 may operate in a nasal capacity and contribute to olfactory lipid detection.
		Sex = not specified	Licking test	
		Groups = control, CD36 knockout; surgical control, olfactory nerve transected	Olfactory nerve transection	
Lee et al. (108), 2017, Japan	*N* = 18 C57BL/6J and CD36 knockout mice	Age = 8–12 weeks	Two-bottle choice test	WT mice discriminated a sucrose solution with oleic aldehyde from sucrose solution alone in the two-bottle choice test. CD36 knockout mice did not discriminate the differences in solutions and fed on both bottles equally. WT mice also exhibited increased exploratory behavior (including sniffing) for an oleic aldehyde vehicle compared to the control, while CD36 knockout mice did not. These behavioral tests display the role of CD36 in fat taste and olfaction.
		Sex = all female	Exploration test to assess sniffing behavior	
		Groups = WT (*n* = 8) and CD36 knockout (*n* = 10)		
Liu et al. (109), 2021, USA	*N* = 5–10 mice for nerve analysis, 16 mice for CTA assays	Age = 2–6 months	Calcium imaging	GPR84 mRNA expression was found in mouse fungiform and CV papillae. MCFAs were found to activate mouse TBCs via increases in intracellular calcium concentration. *Gpr84^−/−^* mice also exhibited significantly reduced taste nerve response to MCFAs and reduced taste responsiveness in the controlled taste aversion assay compared to WT. GPR84 is therefore implicated in the detection of MCFAs.
		Sex = all male	CT nerve recording	
		Groups = WT and *Gpr84^−/−^*	Conditioned taste aversion assay	
Makarova et al. (110), 2021, Russia	*N* = 37 C57Bl/6J diet-induced obese mice 24 mice in experimental groups	Age = 12–26 weeks	Administration of HFD to induce obesity	In females, they found that FGF21 administration reduced the preference for fatty food. However, food intake was not significantly different.
		Sex = both: females (*n* = 10) and males (*n* = 14)	Injection of PBS control or FGF21	
		Groups = control PBS (*n* = 12) and FGF21 (*n* = 12)	Real-time PCR	
Mathes et al. (111), 2015, USA	*N* = 40 (2 groups of 20) Sprague-Dawley rats	Age = 2 weeks apart. First set: 2 months and second set: 1.5 months of age	Progressive ratio (PR) behavioral task	When tested before surgery while nondeprived, HFD rats had lower PR breakpoints (number of operant responses in the last reinforced ratio) for sucrose, but not for Ensure, than CHOW rats. After surgery, at no time did rats given RYGB show lower breakpoints than SHAM rats for Ensure, sucrose, or when 5% Intralipid served postoperatively as the reinforcer.
			Surgery and recovery procedure	
		Sex = all male	Two-bottle preference test	
			Food-deprived testing	
		Groups = chow group (average body weight = 262 g) and HFD group (average initial body weight = 225 g)	Kruskal-Wallis tests	
			Friedman tests	
Murtaza et al. (112), 2017, France	*N* = 20 C57B1/6J mice	Age = 12 weeks	Zizyphin extraction and purification	Preference for LA solution was significantly increased when zizyphin was added to a LA solution (compared to LA alone), suggesting it is involved in modulating fatty acid perception. Zizyphin trigger opening of Ca^2+^ channels in hTBC. Zizyphin does not act on fatty acid receptors.
		Sex = all male	Measurement of calcium signaling in human taste bud cells (hTBCs)	
		Groups: WT (*n* = 10), Gpbar1 -/- (*n* = 10)	Two-bottle preference test	
Murtaza et al. (113), 2020, France	*N* = not specified wild-type C57BL/6J mice	Age = 2 months old	Isolation and culture of mTBCs	In cultured mouse and human TBCs, TUG891 induced a rapid increase in Ca^2+^ by acting on GPR120. LA, also recruited Ca^2+^ via GPR120 in human and mouse TBCs. Both TUG891 and LA induced ERK1/2 phosphorylation and enhanced in vitro release of glucagon-like peptide-1 from cultured human and mouse TBCs. Mice exhibited a spontaneous preference for solutions containing either TUG891 or LA instead of a control. However, addition of TUG891 to a solution containing LA significantly curtailed fatty acid preference.
		Sex = all male	Ca^2+^ signaling measurement	
		Groups = one group received the test solution and the other a control solution	Western blot analysis	
			GLP-1 measurement	
			ELISA	
			Licking test	
			Two-bottle test	
Murtaza et al. (114), 2021, France	*N* = 3–5 mice/condition	Age = 2 months	Measuring of Ca^2+^ signaling in TBC	TRPC3 was found to play a role in the orosensory detection of dietary lipids. Inactivation of TRPC3 in mTBCs caused a decreased in fatty acid induced Ca^2+^ signaling. Preference for a dietary LCFA was also abolished in TRPC3 KO mice. The same effect was seen in mice where TRPC3 was blocked via lingual application of an siRNA.
		Sex = all male	Two-bottle preference test	
		Groups = WT C57BL/6J and TRPC3^−/−^	TRPC3 knockdown by siRNA	
Ozdener et al. (115), 2014, USA	*N* = 20–40 cells per experiment/run human and C57BL/6J mice taste bud cells (TBCs)	Age = not applicable (isolated cells)	siRNA transfection	High concentrations of LA induced Ca^2+^ signaling via CD36 and GPR120 in human and mice TBC; low concentrations induced Ca^2+^ signaling via only CD36. Incubation of human and mice fungiform TBC with linoleic down-regulated CD36 and up-regulated GPR120 in membrane lipid rafts. Fungiform TBC from obese mice had reduced levels of CD36 and increased levels of GPR120 in lipid rafts. Therefore, CD36 is necessary for fat detection, while GPR120 only amplifies response of high concentrations of LA, acting downstream of long-chain fatty acid receptors.
		Sex = not specified	Isolation of CD36^−/−^	
		Groups = CD36 and GPR120 siRNA-transfected human TBC, LA-human TBC, Grifolic acid-human TBC, CD36-/-, WT lean and WT obese TBC	Measurement of Ca^2+^ signaling in TBC	
			Serotonin and GLP1 secretion measurement	
Peterschmitt et al. (116), 2018, France	*N* = 6 mice per group	Age = 6–10 weeks old	Addition of LA to circumvallate papillae	LA induced a significant increase in c-Fos expression in the nucleus of the solitary tract (NTS), parabrachial nucleus (PBN), and ventroposterior medialis parvocellularis (VPMPC) of the thalamus, which are the regions known to be activated by gustatory signals. LA also triggered c-Fos expression in the central amygdala and VTA, involved in food reward, in conjunction with emotional traits.
		Sex = all male	Immunocytochemical localization of c-Fos	
		Groups = lingual application of LA group vs. no application	mRNA expression of BDNF, Sif-268, and Glut-1	
Ricci et al. (117), 2018, Italy	*N* = 7–10 (per group) C57BL/6J *Prep1i/+* mice	Age = 6 months	Macromorphological analysis of brain structural alterations	Prep1 deficiency alters olfactory morpho-functional integrity and olfaction-mediated eating behavior by affecting BDNF-TrkB signaling. Prep1 could play an important role in behavioral dysfunction associated with responsiveness to BNDF.
		Sex = all male	Hemalum and COX Staining	
		Groups = macromorphological analysis and COX staining- C57BL/6J (*n* = 7) Prep1^i/+^ (*n* = 7) for behavioral-C57BL/6J (*n* = 9) and Prep1^i/+^ (*n* = 9)	Immunofluorescence	
			Behavioral (open field, olfactory preference, food preference)	
			Western blotting	
			Real-time (RT-PCR)	
			Cell viability assay	
Sakamoto et al. (118), 2015, Japan	*N* = 8–12 (per group) BALB/c mice	Age = 8 weeks old	Two-bottle choice test	The opioid system seems to have a greater role in determining the palatability of high-fat foods unlike the contribution of olfactory and glossopharyngeal nerves.
		Sex = all male	Olfactory nerve transection (ONX)	
		Groups = water vs. intralipid (*n* = 12), water vs. intralipid +/- naltrexone (0.5 or 2 mg/kg, *n* = 8 Sham vs. ONX (*n* = 8) Sham vs. GLX (*n* = 8) ONX and GLX +/- naltrexone (*n* = 12*)*	Glossopharyngeal nerve transection (GLX)	
			Operant lever-press paradigm: progressive (PR) schedule	
Sakamoto et al. (119), 2015, Japan	*N* = 7–10 (per group) BALB/c mice	Age = 8 week old	Two-bottle choice test	In mice, preference of fat relies strongly on the opioid system, while that of sucrose is regulated by other mechanisms in addition to the opioid system.
			Preference between sucrose and intralipids in naive mice + opioid receptor antagonists	
		Sex = all male	Preference between sucrose and intralipids following naltrexone	
			Preference between sucrose and intralipids following food deprivation in naive mice	
		Groups = saline, naloxanazine, naltrindole, Nor-BNI (*n* = 8) saline vs. naltrexone (*n* = 8) food deprivation (*n* = 7) Saline vs. naltrexone compared to water in naïve (*n* = 8) Saline vs. naltrexone in licking behavior (*n* = 10)	Preference between sucrose and intralipids compared to water in naive mice	
			Licking behavior for sucrose and intralipids in naive mice	
Sasaki et al. (120), 2017, Japan	*N* = 3–10 (per group) C57BL/6J	Age = 8–10 weeks old; except for in CTA experiment mice were 12–14 weeks	d-serine IP injection effect on HFD consumption	IP-injected d-serine inhibited HFD intake and acquisition of an HFD preference. Individual mice with the same genetic background showed different sensitivities to d-serine; thus d-serine sensitivity may be associated with unidentified traits.
		Sex = all male	d-serine IP injection effect on CTA	
		Groups = HFD vs. NC + saline or IP d-serine day 0 (*n* = 6), day 1 (*n* = 7), day 2 (*n* = 8), and day 4 (*n* = 7) IP d-serine (*n* = 9) vs. LiCl (*n* = 8) brain d-serine (*n* = 3), and L-serine (*n* = 3) post-IP D-serine IP D-serine vs. VEH (*n* = 6/group) IP D-serine + water or lipid emulsion (*n* = 6/group)	d-serine and l-serine levels pre- and post-IP d-serine	
			Single IP d-serine injection effect on HFD preference effect of intraperitoneally injected d-serine under the single-food access paradigm using liquid meals (water or lipid emulsion)	
Schreiber et al. (121), 2020, USA	*N* = 4–10 (per group) OP and OR S5B/PI rats	Age = 8–9 weeks old	Transection of glossopharyngeal nerves	OR rats had a higher fungiform papillae density than OP rats. Transection of glossopharyngeal nerves decreased HFD intake in OR rats and had no change in OP rats.
		Sex = all male	Comparing the effect of GLX/CTX on quinine intake	
		Groups = fungiform papillae assessment: OP (*n* = 6), OR (*n* = 5)	Comparing the effect of GLX/CTX on HFD and LFD intake	
		Effect of GLX/CTX on quinine intake: OR-Sham (*n* = 5), OR-GLX/CTX (*n* = 5), OP-Sham (*n* = 4), OP-GLX/CTX (*n* = 4)		
		Effect of GLX/CTX on HFD and LFD intake: OR-SHAM-LFD (*n* = 10), OR-GLX/CTX-LFD (*n* = 8), OR-SHAM-HFD (*n* = 11), OR-GLX/CTX-HFD (*n* = 10), OP-SHAM-LFD (*n* = 11), OP-GLX/CTX-LFD (*n* = 8), OP-SHAM-HFD (N = 10), OP-GLX/CTX-HFD (*n* = 9)		
Sclafani and Ackroff (122), 2014, USA	*N* = 11 (per group) P2X2/P2X3 double knockout mice (P2X DoKO)	Age = 11–14 weeks old	Utilize P2X DoKO to examine maltodextrin preference for polycose solutions (vs. water)	Preference of mice for maltodextrin and fat are dependent on adenosine triphosphate taste cell signaling. With experience, however, P2X DoKO mice develop strong preferences for the nontaste flavor qualities of maltodextrin and fat conditioned by postoral actions of these nutrients.
		Sex = all male	Utilizes P2X DoKO to examine fat preference to intralipid (vs. water)	
		Group = P2X DoKO (*n* = 11), WT (*n* = 11)		
Sclafani et al. (123), 2015, USA	*N* = 12 per group GPR40/120 double knockout and C57BL/6J wild-type mice	Age = 15 weeks old	Utilizes DoKO GPR40/120 to examine if GPR40 and GPR120 play a role in intralipid and glucose preference	Postoral GPR40/120 signaling is not required to process IG fat infusions in food-baited spout training sessions but contributes to postoral fat reinforcement in empty spout tests and flavor conditioning tests.
		Sex = all male		
		Group = GPR40/120 double knock out (*n* = 12), C57BL/6J WT (*n* = 12)		
Sclafani et al. (123), 2015, USA	*N* = WT C57BL/6J and GHSR null mice	Age = adult-age not specified	Utilizes GHSR-null mice	Ghrelin receptor signaling is not required for flavor preferences conditioned by the oral or postoral action of sugar and fat.
		Sex = both:		
		Exp1: 9 females, 10 males	Flavor conditioning	
		Exp2: male		
		Exp3: 9 female, 13 males	Intragastric feeding	
		Exp4–9: female, 13 males		
		Exp5–11: female, 11 males		
		Exp6–12: female, 12 males		
		Groups:		
		Exp1: GHRS-null vs. WT+/− CS+/CS-		
		Exp2: CS+/glucose, CS+ S + S, CS-/S + S		
		Exp3: GHRS-null mice vs. WT+ CS+/CS- +GHSR antagonist		
		Exp4: GHRS-null mice vs. WT+ CS+/CS- +IG intralipid/IG water		
		Exp5: food-deprived GHRS-null mice vs. WT+ CS+/CS- +IG intralipid/IG water Exp6- GHRS-null mice vs. WT		
Sclafani and Ackroff (124), 2018, USA	*N* = CAST/EiJ (*n* = 10) and C57BL/6J mice (*n* = 10)	Age = 9 weeks old	Compare the preferences of CAST and B6 mice for fat vs. sugar and maltodextrin vs. water in 2-day choice tests	CAST/EiJ mice strongly prefer fat to isocaloric carbohydrate (sucrose, maltodextrin). C57BL/6 J mice show the opposite preference profile. CAST/EiJ mice show weaker fat preferences in fat vs. water tests compared to C57BL/6 J. CAST/EiJ mice, like C57BL/6 J mice strongly prefer sweetened fat to maltodextrin. Taste rather than postoral factors is implicated in the low-fat preferences of CAST/EiJ mice.
		Sex = all male		
		Groups = CAST & B6		
Sclafani and Ackroff (125), 2018, USA	*N* = not specified CD36 KO mice	Age = 11 weeks	Use of CALHM1 knockout (KO) to evaluate the primary role of CD36 as a taste receptor mediating fat preference	Naïve CD36 KO mice displayed reduced preferences for soybean oil emulsions (intralipid) at low concentrations (0.1–1%). CALHM1 KO mice displayed even greater Intralipid preference deficits compared with WT and CD36 KO mice. This suggests there may be other taste receptors other than CD36 (but also through CALHM1). After experience with concentrated fat (2.5–5%), CD36 KO and CALHM1 KO mice displayed normal preferences for 0.1–5% fat, the experience-induced rescue of fat preferences in KO mice can be attributed to postoral fat conditioning.
		Sex = all male		
		Groups = CAST & B6		
Subramaniam et al. (126), 2016, France	*N* = 164 C57B1/6J mice, X Erk-1-/- C57B1/6J mice, X calhm1-/- C57B1/6J mice	Age = 22.2 ± 1.8	Measurement of calcium signaling	Fat preference is decreased by downregulation of ERK1/2. LA induces MAPK activation in hTBCs. Src-kinases and raft integrity are involved in LA-induced ERK1/2 activation in hTBCs. LA induces ERK1/2 phosphorylation via CD36 in hTBCs. CALHM1 channels are upstream regulators of LA-induced ERK1/2 phosphorylation. LA-induced Ca^2+^ signaling and ERK1/2 phosphorylation are impaired in Calhm1^−/−^ TBCs. Preference for fat is abolished in Calhm1^−/−^ mice.
		Sex = both: male (*n* = 2), female (*n* = 17)	Lipid raft isolation	
		Groups = orosensory detection of LA	Licking and 2-bottle preference tests	
Tauber et al. (127), 2017, USA	*N* = 9–49 *Drosophila* flies per experiment	Age = 7–9 days old	Measured proboscis extension reflex	Neurons expressing IR56d are necessary and sufficient for reflexive feeding response to FAs in *Drosophila*. IR56d/Gr64f neurons are activated by medium-chain FAs and are sufficient for reflexive feeding response to FAs. Flies can discriminate between sugar and FAs in an aversive taste memory assay, indicating that FA taste is a unique modality in Drosophila.
		Sex = all female	Uses Ca^2+^ sensor GCAMPS under control of, e.g., Gr64g-GAL4, IR56d-GAL4, etc.	
		Groups = not specified	Selective silencing and expression	
			Examination of neuronal activity	
Tsuzuki et al. (128), 2016, Japan	*N* = not specified	Sex = not specified	oxLDL-CD36 binding/inhibition assays	*Z,Z*-TTD is an odor-active fatty aldehyde that is recognized by CD36 in the (nose). Aldehyde functional groups on fatty acid chains are especially important for CD36 lipid recognition. This suggests that CD36 in the mucus layer of olfactory tissue binds to specific lipids and relays them to olfactory receptors.
		Groups = control and CD36 peptide residues	Fluorescence measurement	
Weiss et al. (129), 2019, USA	*N* = 78 male Sprague-Dawley rats	Age = 8 weeks	Body composition monitoring	A high-energy diet produces blunted, but more prevalent, responses in the nucleus of the solitary tract (NTS), and weaker association of taste responses with ingestive behavior.
		Sex = all male	Taste stimuli and odor stimuli testing	
		Groups = diet-induced obese (*n* = 39 ); lean (*n* = 39)	Electrophysiology testing in operant chamber	
Wu et al. (130), 2017, Korea	*N* = xC57BL/6J WT, ob/ob, and Olf544^−/−^ mice	Age = 8 weeks old	Microarray + qPCR of olfactory receptors in liver and adipose tissue	Azelaic acid (a FA) is a ligand of Olfactory receptor 544 (Olfr544). Olfr544 orchestrates the metabolic interplay between the liver and adipose tissue, mobilizing stored fats from adipose tissue and shifting the fuel preference to fats in the liver and BAT.
			CRISPR/Cas9 to generate Olfr544-/-	
		Sex = all male	Administration of 60% HFD + 50 mg/kg of Olfr544 agonist AzA	
			OGTT + ITT	
		Groups = HFD + azelaic acid (AzA), Olf544^−/−^, and ob/ob	Micro-CT for adipocyte tissue measures	
			Histological analysis of BAT	
			Quantification of hepatic triglycerides	
			Indirect calorimetry	
Xavier et al. (131), 2016, Brazil	*N* = 6 Cd36*^obl^*/Mmucd mice, *n* = 6 Olfr*^17tm7Mom^* MomJ mice, and *n* = 4 C57BL/6JB WT mice	Age = 4 weeks old	RT-PCR for *Cd36* transcripts in olfactory epithelium	The Cd36 receptor is highly expressed in a small subset of mature olfactory sensory neurons in the main olfactory epithelium. In addition, the main olfactory epithelium expresses distinct populations of sensory neurons, typically dependent on the receptor they express. Cd36-deficient mice show normal general olfactory behaviors but do not show a preference for a lipid-odor mixture compared to WT mice.
		Sex = all male	In situ hybridization and immunofluorescence staining to determine which cells Cd36 is present in	
		Groups = Cd36^obl^/Mmucd and wild-type C57BL/6J and Olfr^17tm7Mom^/MomJ (P2-IRES-tauGFP) mice	Behavioral assays to study olfactory behavior upon lipid exposure	
Yasumatsu et al. (132), 2018, Japan	*N* = 7–9 C57BL/6JB WT and GPR120-KO mice per group	Age = 8–20 weeks	Single-fiber nerve response recordings from CT nerve	Pharmaceutical blockade of GPR120 caused suppression of CT nerve responses to fatty acids in WT mice. GPR120-KO mice also exhibited a higher threshold for LA detection than WT mice.
		Sex = all male	CTA experiments on WT and GPR120-KO mice	
		Groups = WT and GPR120-KO	Chemical stimulation of the tongue with GPR120 antagonist	

WT, wild type; HFD, high-fat diet; CS, conditioned stimuli; OLA, oleic acid; TBC/LPS, taste bud cells/mouse tas; lipopolysaccharide; CV, circumvallate; LA, linoleic acid; IP, intraperitoneal; CFP, conditioned flavor preference; RD, regular diet; NMDA, *N*-Methyl-d-aspartate; RT-qPCR, quantitative polymerase chain reaction/reverse transcription polymerase chain reaction; PER, proboscis extension reflex; IPGTT, intraperitoneal glucose tolerance test; OGTT, oral glucose tolerance test; CRISPR, clustered regularly interspaced short palindromic repeats; CS, conditioned stimulus; GLP-1, glucagon-like peptide 1; ELISA, enzyme-linked immunosorbent assay; FGF21, fibroblast growth factor 21; BDNF, brain-derived neurotrophic factor; OP, obesity prone; OR, obesity resistant; COX, cytochrome *c* oxidase; OSNs, olfactory sensory neurons; ONX, olfactory nerve transection; GLX, glossopharyngeal nerve transection; GHSR, growth hormone secretagogue receptor; OGTT, oral glucose tolerance test; ITT, insulin tolerance test; CT, computed tomography; BAT, Bayesian analysis toolkit; TRPC, transient receptor potential canonical; CD36, cluster of differentiation 36.

### 2.5. Risk of Bias Assessment

To assess the risk of bias in the clinical studies, we used three JBI (formerly known as Joanna Briggs Institute) Critical Appraisal Tools: Checklist for Analytical Cross-Sectional Studies (8 questions) (133); the Checklist for Randomized Controlled Trials (13 questions) (134); and the Checklist for Cohort Studies (11 questions) (135). All JBI checklists included questions and guided instructions to systematically assess each study based on multiple criteria (e.g., reliability and validity). To assess preclinical animal intervention studies, we utilized the SYstematic Review Center for Laboratory Animal Experimentation (SYRCLE) risk of bias tool (RoB) (10 questions) (136). The checklists and scoring we used are included as supplementary materials (Supplemental File S2–3).

To quantitatively score each JBI and SYRCLE RoB checklist, each of the questions received a score between zero and two; zero (cannot tell), one (no), and two (yes). Total possible scores for the Checklist for Analytical Cross-Sectional Studies could range from 0 to 16; 0 to 26 for the Checklist for Randomized Controlled Trials; 0 to 22 for the Checklist for Cohort Studies; and 0 to 20 for the SYRCLE RoB. Higher scores for each checklist indicated a lower risk for bias.

A sample of four articles was used by all reviewers (R.B.J.-L., C.V., B.E.B., R.S.E.O-F., M.C., N.N., and P.V.J.) participating to pilot test the selected checklist tools and discuss problems and questions with the tools and risk of bias assessment step.

Two reviewers independently assessed the risk of bias for each included study, and a third reviewer checked the completed checklists for discrepancies. Any errors or discrepancies were resolved by discussion between the two reviewers who completed the checklist and the third reviewer until a consensus was reached for the specific question.

Studies were not excluded from the analyses based on their risk of bias score. Any uncertainty about a study’s risk of bias was discussed as a group.

### 2.6. Effect Measures and Synthesis Methods

The effect measures collected are shown in TABLE 1. The age, sex, and body mass index (BMI) were collected as reported in the article. If it was possible to calculate the mean for age and BMI only based on the data provided in the article, this was done. Descriptive statistics were calculated, and a narrative synthesis of the findings was completed.

### 2.7. Certainty Assessment

No assessment of the overall certainty or confidence in the included studies was completed as we included both human clinical studies and animal studies, which were not comparable, and the animal studies overall had a very low risk of bias scores.

## 3. RESULTS

### 3.1. Selected Studies

**FIGURE 5.**
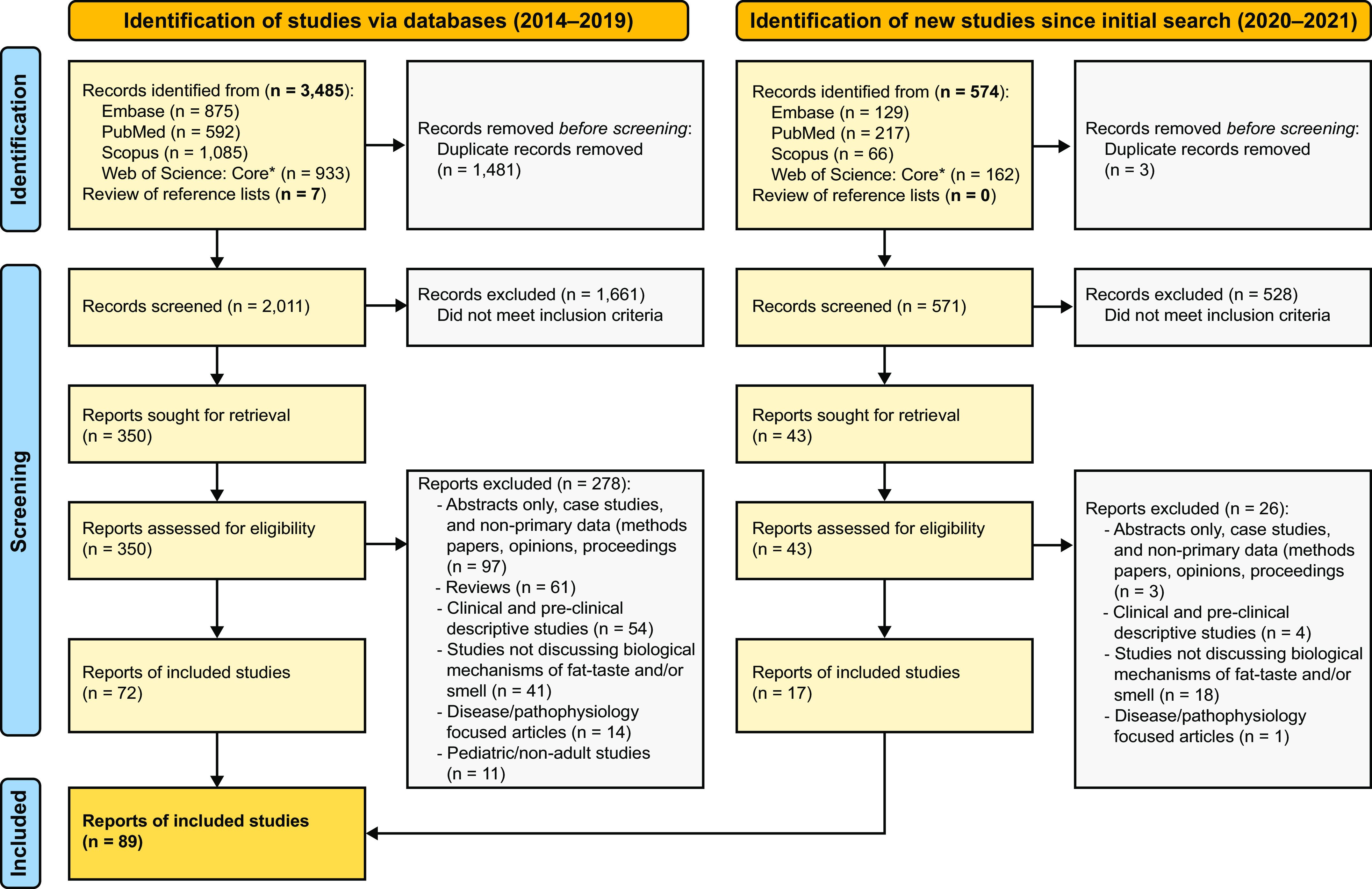
PRISMA flow diagram. The diagram shows the selection of reports included in this systematic review.

The initial search resulted in a total of 4,059 articles retrieved and manual searching of reference lists of included studies resulted in 7 additional articles (FIGURE 5). After duplicates were removed (*n* = 1,484), the remaining 2,582 article titles and abstracts were assessed for relevance, and studies that did not meet inclusion criteria were excluded. Studies that did not discuss biological mediators of fat taste or smell were excluded (*n* = 2,189). Of the 393 remaining articles, 304 were excluded after screening the full text. Studies not included due to the exclusion criteria are summarized in FIGURE 5. After each study was assessed using exclusion criteria, 89 articles were included in this systematic review. The heterogeneity and diverse methods between the studies prevented the conduct of a meta-analysis.

### 3.2. Study Characteristics

This review included 37 clinical (TABLE 1) and 52 preclinical (TABLE 2) studies. Clinical studies had sample sizes ranging from 10 to 736 participants. 83.78% of clinical studies included both male and female participants. However, three studies included only females and three included only males. Study designs encompassed: randomized control, cross-sectional, crossover design, case-control, and cohort (prospective observational) studies. Clinical study methods included: psychometric tests such as taste preference [Alternative-Forced Choice (AFC)], detection threshold [e.g., general Label Magnitude Scale (gLMS)], and nutritional assessments [e.g., Food Frequency Questionnaire (FFQ)]. Articles in this review were set in 22 countries and the United Kingdom. Clinical studies were conducted in Australia (*n* = 4), Canada (*n* = 1), Czech Republic (*n* = 1), Denmark (*n* = 1), Finland (*n* = 1), France (*n* = 3), Germany (*n* = 3), Italy (*n* = 4), Japan (*n* = 1), India (*n* = 1), Malaysia (*n* = 1), Mexico (*n* = 1), Morocco (*n* = 1), Poland (*n* = 1), Spain (*n* = 1), Tunisia (*n* = 2), United Kingdom (*n* = 5), and the United States (*n* = 5). Preclinical studies were conducted in Belgium (*n* = 1), Brazil (*n* = 1), Chile (*n* = 1), France (*n* = 11), Italy (*n* = 1), Japan (*n* = 7), Korea (*n* = 3), Mexico (*n* = 1), Russia (*n* = 1), and the United States (*n* = 25).

Seven preclinical studies included both sexes, 35 were conducted in males, 6 in females, and 4 were not specified. Study characteristics are summarized in TABLE 2. Preclinical studies were conducted on animal and in vitro models, including *Mus musculus* (mice; *n* = 36), *Rattus sp.* (rats; *n* = 9), *Drosophila melanogaster* (fruit flies; *n* = 6), and *Homo sapiens* (human cells; *n* = 1). The preclinical study sample size varied by group and total sample size. TABLE 2 summarizes group and total sample size for each study.

### 3.3. Risk of Bias Assessment

Total score for the JBI Checklist for Analytical Cross-Sectional Studies ranged from 9 to 16 (*n* = 29) (highest score = 16); 11 to 19 for the Checklist for Randomized Controlled Trials (*n* = 5) (highest score = 26); 10 to 20 for the Checklist for Cohort Studies (*n* = 3) (highest score = 20); and 1 to 15 for the SYRCLE RoB (*n* = 52) (highest score = 20). See Supplemental File S3 for a final risk of bias scores for the clinical and preclinical/animal included studies.

## 4. DISCUSSION

Studies included in this systematic review demonstrate advances in understanding the biological mediators of fat taste and smell. Studies focus on five themes: molecules and their biological pathways, neuroanatomical regions, ingestive cues, postingestive cues, and genetic variability associated with fat chemosensation. Finally, we will discuss the role of fat taste and smell in the context of eating behavior and obesity. In this section, we will discuss each theme. First, we will summarize and describe the molecules involved in fat taste and smell.

### 4.1. Molecules, Transduction Pathways, and Ingestive Cues Mediating Fat Taste and Smell

**FIGURE 6.**
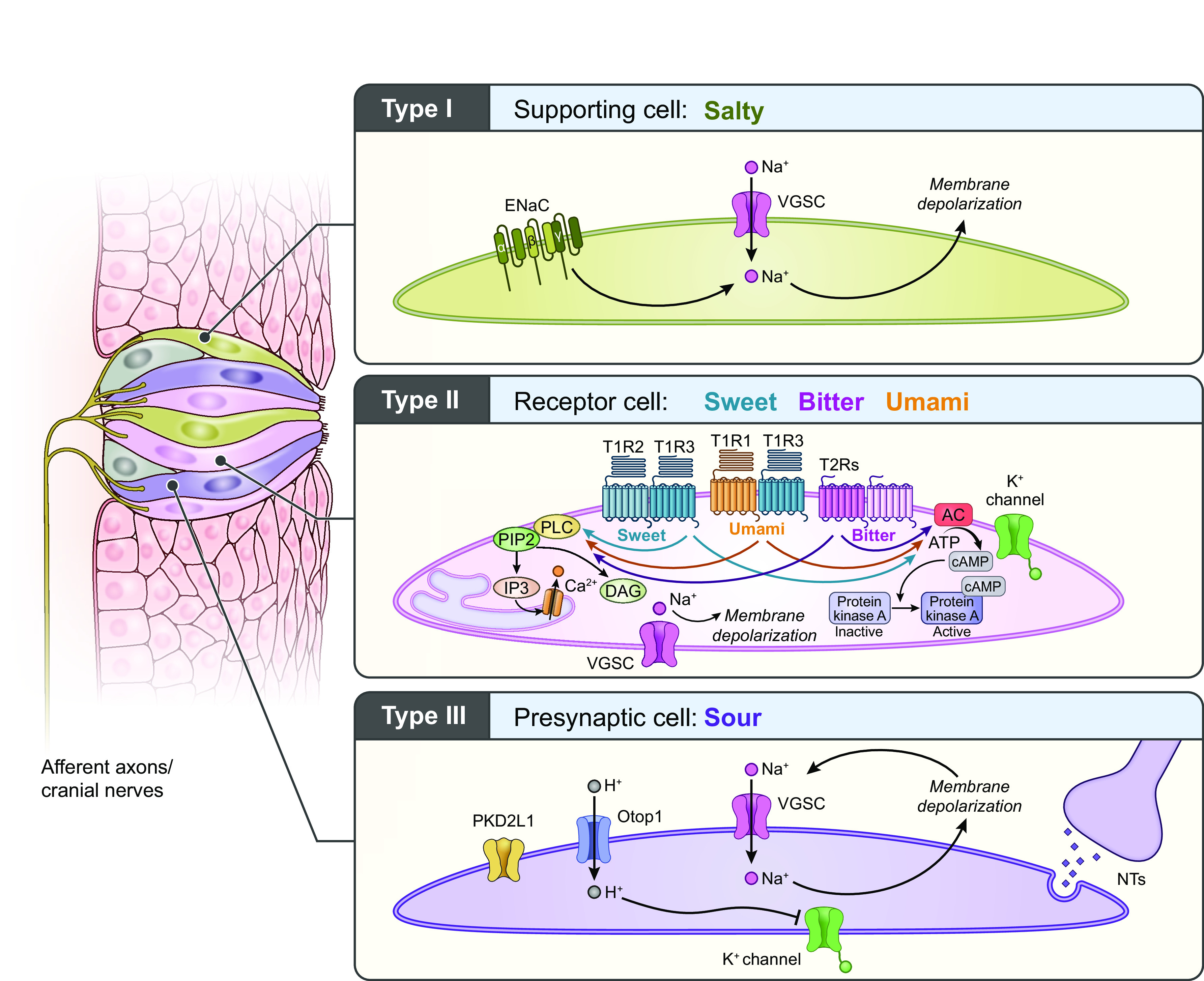
Taste transduction by taste modality. Taste buds contain three cell types specific to taste modalities: type I, type II, and type III cells. Type I cells are glial-like cells and are involved in salt detection. Type II cells are involved in sweet, bitter, and umami. Presynaptic type III cells detect sour stimuli and potentially salty stimuli. Each cell type and each taste modality are characterized by their own combination of receptors and transduction pathways. Taste information is then relayed to the brain via afferent nerves (cranial nerves VII, IX, and X). ENaC, epithelial sodium channel; IP_3_, inositol (1,4,5)-triphosphate; NTs, neurotransmitters; PIP2, phosphatidylinositol 4,5-bisphophate; VGSC, voltage-gated sodium channel. Figure adapted from Ref. 26, with permission from Springer Nature.

**FIGURE 7.**
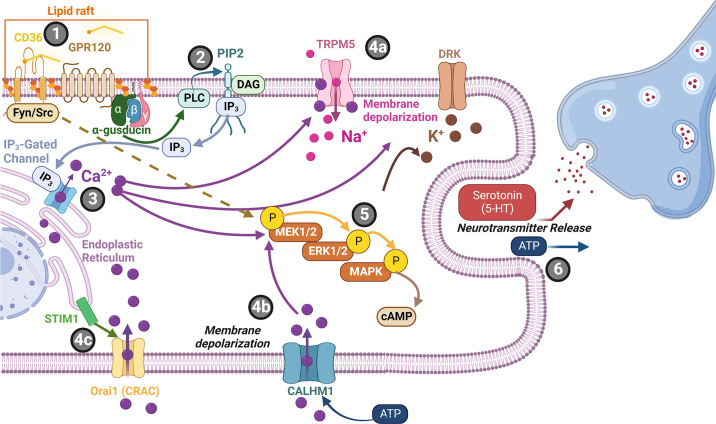
Fat Transduction signaling in taste bud cells. In taste bud cells: (*1*) free fatty acids (FFAs) binds to cluster of differentiation 36 (CD36), and CD36 interacts with GPR120. This triggers a signaling cascade involving α-gustducin which activates Ca^2+^, dependent phospholipase (PLC). (*2*) PLC cleaves phosphatidylinositol 4,5-bisphophate (PIP2; bound to the membrane) into diacylglycerol (DAG) (stays in membrane) and inositol (1,4,5)-triphosphate (IP_3_) (free in cytosol). DAG can phosphorylate protein kinase C (PKC), which helps to activate the extracellular signal-regulated kinases (ERK) pathway. (*3*) IP_3_ leaves the membrane and binds to IP_3_-gated calcium ion channels on the endoplasmic reticulum (ER) membrane. This opens the IP3 channels and releases Ca^2+^ stores from the ER into the cytosol, increasing intracellular calcium levels ([Ca^2+^]_i_). (*4*) The increased [Ca^2+^]_i_ triggers several events that lead to membrane depolarization of the cell. (*4a*) TRPM5 channels (a Ca^2+^-gated sodium channel) open, allowing Na^+^ to come into the cell. Additionally, Delayed rectifying K^+^ (DRK) channels close, preventing potassium from leaving the cell. (*4b*) The calcium release from the ER requires that stromal interaction molecule 1 (STIM1) replenish the ER’s calcium stores. (4c) STIM1 activates Orai1 (calcium release-activated calcium channel) to enable the influx of calcium into the cytosol. Subsequent opening of calcium homeostasis modulator 1 (CALHM1) channels allows for additional calcium influx into the cell. (*5*) Increased [Ca^2+^]_i_ and CD36-induced Fyn/Src kinase activation contribute to phosphorylation of the ERK1/2 pathway. This can activate cAMP signaling, which promotes transcription of cellular regulation factors. (*6*) Depolarization throughout the cell leads to the release of serotonin (5-HT) and ATP, which act as neurotransmitters. Serotonin binds to its receptors, and ATP binds to purinergic P2X2 and P2X3 receptors that cause neuronal excitability, further relaying fat chemosensory signals to the brain. CD36, cluster of differentiation 36; GPR120, G protein-coupled receptor 120. Image created with BioRender.com, with permission.

Taste and olfactory receptors play a key role in the detection of chemical stimuli in the environment, including fat and other taste and smell stimuli. Different taste modalities and olfactory receptors are important in initiating transduction pathways that ultimately relay information about the tastes and smells we perceive. There are three different types of taste cells, with each taste modality being characterized by a unique combination of taste receptors and transduction pathways (summarized in FIGURE 6). Multiple molecules have been implicated in fatty acid detection, including cluster of differentiation 36 (CD36) and G protein-coupled receptor (GPCR) 120 (GPR120). CD36 is one of the most studied proteins as it displays a high affinity for long-chain fatty acids. For reviews of CD36’s role in fatty acid detection, please see Degrace-Passily et al. (232) and Pepino et al. (141). GPCRs, including GPR120, were also proposed as potential fatty acid detector proteins. For a review that examines GPR120’s role in fatty acid detection, please see Besnard et al. (18). In this systematic review, we summarize emerging literature exploring CD36 and GPR120. We will also discuss other proteins that have been studied in the context of fat chemosensation, including Toll-like receptor 4 (TLR4), calcium homeostasis modulator 1 (CALHM1), Takeda-G-protein-receptor-5 (TGR5), gustatory receptor (GR), ionotropic receptor (IR) proteins, and beta-adrenergic receptor (ADRB3). Many of these proteins act in the TBCs after initial activation by CD36 and GPR120. The role of each protein in fat chemosensation is summarized in TABLE 3 and FIGURE 7.

**Table 3.** Proteins involved in fat chemosensation

Protein	Study (Reference), Year	Findings
α-Gustducin	Avau et al. (86), 2015	α-Gustducin is involved in fat intake and obesity.
	Watanabe et al. (80), 2019	ADRB3 is an essential mediator of fat perception and metabolism in the body. The Trp64Arg variant of this gene is associated with high-fat preference, indicating the structure of the adrenergic receptor protein may play a role in oral fat perception.
ADRB3	Sclafani et al. (124), 2018	CALHM1 KO mice displayed even greater intralipid preference deficits compared with WT and CD36 KO mice. Suggesting that non-CD36 taste receptors also contribute to fat detection and preference. CALHM1 KOs can still develop normal preferences after multiple exposures (not in naïve) attributed to post-oral fat conditionin
CALHM1	Subramaniam et al. (126), 2016	CALHM1 channels are upstream regulators of LA-induced ERK1/2 phosphorylation. LA-induced Ca2+ signaling and ERK1/2 phosphorylation are impaired in Calhm1-/- TBCs. Preference for fat is abolished in Calhm1-/- mice.
	Braymer et al. (89), 2017	CD36 mRNA levels were increased in lean rats.
		Lingual application of CD36 siRNA decreased fat preference in lean, obesity-resistant rats.
CB_1_R	Avalos et al. (85), 2020	CB_1_R knockout (KO) mice displayed an attenuated preference for HFD for the first 6 hours of a preference test compared to WT mice.
CD36	Bricio-Barrios et al. (52), 2019	BMI was associated with low serum CD36 and lower fat sensitivity.
	Djeziri et al. (96), 2018	CD36 inhibition prevented lipid-induced intracellular calcium increases. CD36 expression was decreased with HFD alone but increased with oleic acid.
	Gaudet et al. (99), 2019	Continuous access to HFD increased lingual CD36 expression in rats.
	Lee et al. (107), 2015	CD36 contributes to lipid recognition in mice.
	Lee et al. (108), 2017	Wild-type, but not CD36-knockout mice, were able to detect oleic aldehyde, providing evidence for the involvement in CD36 in in the perception of dour-active volatile compounds in the nasal cavity.
	Ozdener et al. (115), 2014	High concentrations of linoleic acid induced Ca^2+^ signaling via CD36 and GPR120 in human and mice TBC, as well as in STC-1 cells, and low concentrations induced Ca^2+^ signaling via only CD36. CD36 and GPR120 have nonoverlapping roles in TBC signaling during orogustatory perception of dietary lipids; these are differentially regulated by obesity.
	Sclafani et al. (124), 2018	CD36 KO reduced preference for lipids (in naïve CD36 KO).
	Subramaniam et al. (126), 2016	CD36 is involved in FA induction of ERK1/2 phosphorylation. LA induced phosphorylation of MEK1/2-ERK1/2ETS-like transcription factor-1 cascade via CD36 in human TBCs.
	Tsuzuki et al. (128), 2016	CD36 may be expressed in the nasal cavity and binds to fatty aldehyde. Nasal CD36 can signal to olfactory neurons.
	Xavier et al. (131), 2016	CD36-deficient mice did not demonstrate changes in the organization of the olfactory epithelium but showed impaired preference for a lipid mixture odor. CD36-expressing neurons represent a distinct population of OSNs, which may have specific functions in olfaction.
FFAR4 (GPR120)	Costanzo et al. (144), 2019	FFAR4 in fungiform papillae may play a role in fat perception. FFAR4 expression was positively associated with FAT sensitivity. Increases in FFAR4 may also increase intestinal satiety signals, leading to reduced further fat intake.
GPR120	Ancel et al. (84), 2015	GPR120 is not necessary for fat-taste detection.
	Murtaza et al. (113), 2020	Select GPR120 agonist can trigger intracellular Ca^2+^ increases, induce MAPK phosphorylation, and modulate fatty acid preference. Therefore, GPR120 is involved in fat-taste pathway.
	Ozdener et al. (115), 2014	GPR120 is involved in amplifying transduction response. CD36 and GPR120 have nonoverlapping roles in TBC signaling during orogustatory perception of dietary lipids
	Sclafani et al. (123), 2015	Post-oral GPR40/120 signaling is not required to process IG fat infusions in food-baited spout training.
	Yasumatsu et al. (132), 2018	GPR120 antagonist caused suppressed CT nerve signaling and the reduction of maximal nerve responses in WT mice.
GPR84	Liu et al. (109), 2021	GPR84 is involved in taste detection of MCFAs in TBCs by triggering intracellular Ca^2+^ increases and membrane depolarization.
Gr64e, Gr64f, IR56d	Kim et al. (104), 2018	Gr64e is involved in fat chemosensation, but not direct receptor; another Gustatory receptor required for the behavioral and electrophysiological responses to FA detection.
	Tauber et al. (127), 2017	IR56d/Gr64f neurons are activated by medium-chain FAs and are necessary and sufficient for reflexive feeding response to FAs.
Olfr544	Wu et al. (130), 2017	Olfr544 orchestrates the metabolic interplay between liver and adipose tissue, mobilizing stored fats from adipose tissue and shifting fat preference.
OR4D2, OR51A7, OR2T34, OR2Y1	Ramos-Lopez et al. (75), 2019	OR4D2, OR51A7, OR2T34, and OR2Y1, along with several downstream signaling molecules (SLC8A1, ANO2, PDE2A, CALML3, GNG7, CALML6, PRKG1, and CAMK2D) regulate odor detection and signal transduction processes within the complete olfactory cascade.
Prep1	Ricci et al. (117), 2018	Prep1 deficiency alters olfactory morphofunctional integrity and olfaction-mediated eating behavior.
P2X2/P3X3	Bensalem et al. (87), 2020	TGR5 KOs show changes in fat preference and calcium signaling
TGR5	Camandola and Mattson (93), 2017	TLR4 promotes fat ingestion (FA endocytosis) and fat taste preference
TRPC3	Murtaza et al. (114), 2021	TRPC3 KO mice TBCs showed significantly curtailed Ca^2+^ signaling in response to LA.

BMI, body mass indes; HFD, high-fat diet; WT, wild type; CD36, cluster of differentiation 36; GPR120, G protein-coupled receptor 120; TLR4, Toll-loke receptor 6; FAs, fatty acids; LA, linoleic acid; TBCs, taste bud cells; MCFAs, medium-chain fatty acids.

#### 4.1.1. Proteins associated with fat detection: CD36, GPR120, and fat taste and smell.

##### 4.1.1.1. cd36.

CD36 is a transmembrane protein with a high affinity for FFAs, such as linoleic acid (LA) and oleic acid (OLA) (43, 69, 70, 126, 137), and a hydrophobic region that acts as a binding for these ligands (138, 139). Extra-orally, CD36 is expressed in multiple tissues (e.g., the nasal epithelium, brain, and cardiovascular tissue) (131), and it plays a role in immune response, inflammation, and angiogenesis (140). In the mouth, CD36 is located on the apical surface of TBCs (43, 137) where it binds to FFAs. For example, a 2016 study by Subramaniam et al. (126) demonstrated that in human TBCs, LA binds to CD36, which activates downstream pathways. This triggers GPCR-dependent secondary signaling cascades that ultimately depolarize cells and/or contribute to neurotransmitter release (43). These signaling cascades are implicated in sweet and bitter taste, including cyclic adenosine monophosphate (cAMP), inositol triphosphate (IP_3_), and phospholipase C (transduction signaling cascades are discussed in sect. 3) (141). The role of CD36 in fat taste and olfaction is examined in 20 studies, summarized in TABLES 3 and 5. Ten studies discussed the effect of CD36 polymorphisms (described in sect. 4) on fat perception. This section will discuss 10 studies that examined the function and expression of CD36 in the context of fat perception (89, 96, 99, 107, 108, 115, 124, 126, 128, 131). Together, these studies highlight the role of CD36 as a fat-detection protein that initiates fat taste transduction.

Among the included studies, four examined the necessity of CD36 in fat taste. These studies consistently found that CD36 played a role in fat preference and/or sensitivity (89, 107, 115, 124). These studies also suggested that CD36 works in conjunction with other proteins to mediate fat chemosensation. Lee et al. (107) determined that CD36 contributes to lipid recognition of irritant1-(palmitoyl)-2-(5-keto-6-octanedioyl)-phosphatidylcholine (KOdiA-PC) (a CD36 ligand) triggering a taste aversion response. *Cd36* knockout mice (KO) mice had a diminished ability to detect this phospholipid. However, *Cd36* KO mice were still able to detect KOdiA-PC at specific concentrations (i.e., 7.5 μM), suggesting CD36 is not the sole KOdiA-PC receptor (107). This is supported by Sclafani and Ackroff (124), who found that *Cd36* KO mice demonstrated lower preference for lipids than wild-type mice. Additionally, naïve *Cd36* KO mice showed reduced taste preference for fat emulsions solutions compared to wild-type (WT) mice. However, *Cd36* KOs developed normal preference following exposure to fat. This is consistent with the hypothesis that CD36 is not the sole fat detection receptor and that plasticity occurs with fat exposure (107, 124).

The role of CD36 in fat detection was also examined in rats. Braymer et al. (89) found that interfering with lingual *Cd36* expression using small interfering RNA (siRNA) in obesity-resistant rats reduced the preference for LA. This suggests that interfering with *Cd36* expression reduces orosensory detection of FFAs. The role of CD36 and its interaction with other proteins in fat detection was also examined in human cells. Ozdener et al. (115) determined that knocking down lingual *CD36* (using siRNA) suppressed calcium signaling in human TBCs. Moreover, concomitant siRNA knock down of GPR120 and *CD36* further suppressed calcium signaling in human TBCs, suggesting these are parallel mechanisms (115). The interaction of CD36 and GPR120 is further discussed below.

Additionally, three studies examined the relationship between *Cd36* expression and fat preference. Djeziri et al. (96) reported that chronic (16 weeks) ad libitum access to a high-fat diet (HFD) altered fat preference and *Cd36* expression in female mice TBCs. They found that HFD-fed mice had significantly lower lingual *Cd36* mRNA expression and decreased preference for fat. However, Gaudet et al. (99) reported that short-term (2 weeks) continuous access to HFD increased lingual *Cd36* expression in male rat’s TBCs and limited intermittent access to a HFD did not change *Cd36* expression. This discrepancy may be due to differences in sex, length of the HFD exposure, and/or species-related differences. Recently, Olvera Hernández et al. (100) also examined fat preference and *Cd36* expression in lingual tissue (specifically in circumvallate papillae) and the hypothalamus of female and male mice. Specifically, this study examined intergenerational and sex-related effects of maternal undernutrition (UN) on *Cd36* expression and fat preference in adult offspring of UN dams. They found female and male mice born to UN dams exhibited increased expression of Cd36 in taste buds. Further, female mice displayed a higher preference for fat than their male counterparts. Additionally, while males of undernourished dams showed decreased fat preference compared to control males, no differences in preference were observed between UN and control females (100). Thus this study brings to light the importance of sex and transgenerational nutrition on *Cd36* expression and fat preference. Further studies are needed to clarify the relationship between *Cd36* expression and fat preference and whether these effects differ by sex.

While the study of CD36 in fat detection has focused on lingual tissue, recent studies have examined the role of CD36 in the olfactory perception of lipids. Lee et al. (107) observed that transection of the olfactory nerve led to the inability to perceive KOdiA-PC (a CD36 ligand). Although it is unclear if this reduction is CD36-dependent, the authors postulate that CD36 may play a role in the olfactory detection of KOdiA-PC on the surface of the olfactory epithelium (107). Tsuzuki et al. (128) observed that odor-active fatty acids with aldehyde domains bind to CD36 on tissue within the nasal cavity, supporting CD36’s role in nasal fat perception. Aldehyde-containing FFA binding to CD36 sends signals to olfactory neurons and can affect further fat ingestion and eating behavior. Xavier et al. (131) found that CD36-KO mice exhibited an impaired preference for a lipid mixture odor compared to wild-type mice, yet there was no impairment in general odorant detection of predator pheromones or food in CD36-KO mice. A study by Lee et al. (108) compared the detection of oleic aldehyde, an odor-active and volatile fatty aldehyde that binds to CD36, in WT and CD36 KO. They found WT mice, but not CD36 KO, displayed increased exploratory behavior (including sniffing oleic aldehyde). This suggests that CD36 KO mice could have reduced sensitivity to oleic aldehyde. The authors suggest that this supports the involvement of CD36 in olfactory detection of this fatty aldehyde (108). These studies are consistent with previous literature suggesting that CD36 plays an essential role in fat chemosensation.

##### 4.1.1.2. gpr120.

GPR120, also known as free fatty acid receptor 4 (FFAR4), is an important transmembrane GPCR in fat taste transduction. After binding to FFAs, CD36 and GPR120 work together to initiate signaling cascades that ultimately lead to depolarization of TBCs. To begin understanding the role of GPR120 in fat taste perception, studies utilized *Gpr120* KOs. However, although some studies have reported that GPR120 knockout mice demonstrated decreased fatty acid preference (141, 142), others have found that while Gpr120 plays a role in fat chemosensation, it is not necessary for fat taste perception (84, 143). Six studies included in this review discussed the role of GPR120 in fat perception (84, 113, 115, 123, 132, 144). *Gpr120* KO mice demonstrated decreased, but not eliminated, preference for fat (84). This suggests that GPR120 modulates but is not completely necessary in mediating fat taste sensitivity (84). Furthermore, GPR120 activation in TB100 with TUG891, a GPR120 agonist, leads to increased intracellular calcium and MAPK phosphorylation in mice (discussed in sect. 3) (113). Costanzo et al. (144) investigated the role of fat receptor genes, including *GPR120*, and showed that *GPR120* expression in human fungiform papillae was associated with fat taste sensitivity. Furthermore, Ozdener et al. (115) found that GPR120 interacts with downstream proteins to amplify the cellular response to high dietary fat concentrations in human and mouse TBCs. However, the role of GPR120 in oral fat detection was challenged in later studies that suggest that GPR120 may mediate postingestive cues that reinforce fat consumption but is not essential for oral fat detection (84, 123). GPR120 has also been implicated in fat taste sensation via nerve responses. After examining chorda tympani nerve responses to fatty acids in WT mice, Yasumatsu et al. (132) found that 17.9% of nerve fibers showed maximal responses to fatty acids, subsequently naming them F-type fibers. GPR120 antagonists in WT mice suppressed the response to LA in F-type fibers, and the percentage of F-type fibers greatly decreased to 4.0%. In addition, the generalization threshold for linoleic acid in GPR120-KO mice was higher than that in WT mice (132). The results from all six studies indicate that GPR120 plays a facilitator role in TB100 signal transduction, alongside CD36, and reinforces fat preferences via postoral mechanisms. GPR120 and CD36 may operate to influence calcium signaling, which is discussed in sect. 2. GPR120 in the gastrointestinal (GI) tract and other postoral actions are discussed further in sect. 3.

#### 4.1.2. Other molecules involved in fat taste and smell.

##### 4.1.2.1. adrb3.

β-3-Adrenergic receptor (ADRB3) is a protein in the GPCR family that mediates fat breakdown and metabolism (80). Previous literature primarily linked ADRB3 to its function in regulating overall energy expenditure, as its mutations are associated with reduced lipolysis and visceral obesity (145–149). Watanabe et al. (80) found that *ADRB3* Trp64Arg substitution is highly correlated with high-fat food preference in young Japanese women. This polymorphism is discussed in sect. 5. The association between ADRB3 polymorphisms and fat preference indicates that this receptor may play a role in oral fat perception.

##### 4.1.2.2. cb_1_r.

Cannabinoid subtype-1 receptors, belonging to the endocannabinoid system, have been found to play a role in fat taste perception and preference. Avalos et al. (85) investigated the impact of CB_1_Rs in the upper small-intestinal epithelium on preference for a Western-style HFD in mice. The study elucidated that acute preferences for the HFD were inhibited by the global pharmacological blockade of CB_1_Rs by an antagonist, AM251. They also found that CB_1_R^+^ control mice displayed robust preferences for the HFD compared to the standard diet and ate significantly more kilocalories from the HFD throughout the 24-h preference test. CB_1_R^–^ mice did not display a preference for HFD for the first 6 hours of the test and ate fewer kilocalories from the HFD. Similarly, Brissard et al. (150) found that CB_1_R KOs and WT mice treated with rimonabant (a CB_1_R blocker) displayed a significant decrease in their preference for fatty solutions (rapeseed and LA) compared to untreated WT mice. These results provide evidence of a crucial role for CB_1_Rs in the rodent upper intestinal epithelium in acute preferences for food containing high levels of fat (85).

##### 4.1.2.3. fgf21.

Fibroblast growth factor 21 (FGF21) is a protein secreted by the liver in response to metabolic stress (151). It plays a role in coordinating metabolic responses from adipose tissue. In mice, FGF21 reduces body weight and sugar intake but not fat (152). However, human studies have found an association between single-nucleotide polymorphisms in *FGF21* and decreased fat intake (153). The FGF21 single-nucleotide polymorphisms are discussed in sect. 4. Thus FGF21’s ability to aid in healthier macronutrient intake is unclear. Therefore, Makarova et al. (110) examined the effect of FGF21 administration on taste preference for HFD. In FG21-treated males, there was a significant reduction in preference for fatty food (compared to control-treated males). However, in FGF21-treated females, there was a small, but not significant, preference for HFD. These data suggest that FGF21’s effect on fat preference may be sex dependent.

##### 4.1.2.4. gpr84.

Another protein implicated in fat sensing in TBCs is G protein-coupled receptor 84 (GPR84). Although GPR84 has previously been identified as a receptor of medium-chain fatty acids (MCFAs) in the immune system via hematopoietic cells, it was found to be a receptor of MCFAs in the mouth by Liu et al. (109). They identified extensive *Gpr84* mRNA in the fungiform papillae and CVP cells of mice. Cells induced to express *Gpr84* exhibited robust intracellular increases in Ca^2+^ in response to five different MCFAs, including capric and lauric acid. In addition, *Gpr84*-deficient mouse TBCs had a significantly reduced ability to respond to MCFAs via intracellular Ca^2+^ rise and membrane depolarization. *Gpr84*-deficient mice also showed loss of chorda tympani (CT) nerve activity during capric acid stimulation, while nerve responses to all other taste modalities remained intact in the absence or presence of GPR84. These results suggest that GPR84 has a new role in the oral cavity as a gustatory receptor of MCFAs (109).

##### 4.1.2.5. olfr544.

Olfactory receptor 544 (Olfr544) has been studied in the context of fatty acid detection and metabolism. Olfactory receptors in the nasal epithelium, such as Olfr544, initiate depolarizing transduction mechanisms representing the detection and recognition of odors (154). Additionally, *Olfr544* is highly expressed in extra-olfactory tissues such as adipose and hepatic tissue of mice (130). Wu et al. (130) found that azelaic acid (FA) is a ligand of Olfr544. Results showed that Olfr544 mediates the metabolic interplay between the liver and the adipose tissue. Specifically, Olfr544 mobilized stored fats from adipose tissue and shifted the fuel preference to fats in these tissues. Thus this olfactory receptor can detect fatty acids and regulate cellular energy and metabolism.

##### 4.1.2.6. prep1.

Prep1 has also been studied in the context of fat perception. Prep1 is a transcription factor involved in metabolic homeostasis and is highly expressed in the mouse olfactory bulb (155). Ricci et al. (117) found that Prep1 deficiency reduced the preference for high-fat foods via olfaction deficits. *Prep1* hypomorphic heterozygous mice display a scant ability to distinguish odors, which significantly impacts feeding behavior. Furthermore, Prep1 deficiency was also associated with decreased activation of ERK1/2 and decreased brain-derived neurotrophic factor (BDNF) levels (117). ERK1/2 involved in fat transduction signaling via Prep1 is discussed below. Previous studies suggested that BDNF influences olfactory behavior (156, 157). These findings suggest that reduced fat preference observed in Prep1-deficient mice may be mediated by impaired responsiveness to BDNF-induced ERK1/2 phosphorylation.

##### 4.1.2.7. serotonin.

Serotonin (5-hydroxytryptamine) also has an implication in fat taste transduction. Serotonin is a neurotransmitter that is highly expressed in brain tissue and is involved in many mechanisms influencing energy balance, mood, and sleep (70, 158). Increases in serotonin levels can cause abnormal regulation of metabolic processes such as lipolysis and gluconeogenesis (159). Supporting previous research, Ozdener et al. (115) and El-Yassimi (160) also found that FAs triggered serotonin release from TBCs (grifolic acid more so than LA). The authors indicated that TBCs release serotonin downstream of calcium signaling; as a neurotransmitter, it communicates fat taste stimuli to afferent gustatory nerve fibers (115). Confirming serotonin’s involvement in fat taste, Gaudet et al. (99) found increases in serotonin gene (SERT) expression in circumvallate papillae TB100 in response to several days of HFD. These results together support serotonin’s role in fat taste transduction, as a TB100 neurotransmitter.

##### 4.1.2.8. tgr5.

TGR5 is a GPCR, more traditionally known as a bile acid receptor in the GI tract, recently found in TBCs. *Tgr5* KO mice exhibited increased food intake and fat mass. Loss of TGR5 function increased calcium signaling and glucagon-like-peptide-1 (GLP-1) secretion in response to fatty acids in TBCs. *Tgr5*’s role in calcium signaling is discussed below. TGR5’s involvement may underlie a high preference for fat in the development of obesity (87).

##### 4.1.2.9. tlr4.

TLR4 is another protein that was initially believed to be a fat receptor (161). TLR4 is a transmembrane protein that is an important mediator of innate immunity (162). It binds primarily to LPS (163) and several saturated FFAs (164–166). Camandola and Mattson (93) found that *Tlr4* KO mice demonstrated a low preference for fat. The authors postulated that TLR4 promoted fat ingestion and preference by mediating FA endocytosis. However, a subsequent study found that TLR4 is not a fat receptor but rather mediates lipid-induced inflammation. This process is critical in developing chronic low-grade inflammation in the pathogenesis of obesity (167).

##### 4.1.2.10. purinergic receptors.

A receptor family implicated in fat taste is purinergic receptors. While adenosine triphosphate (ATP) is a global energy source, it also acts as a signaling molecule that relays information from taste bud receptor cells to afferent neurons (168–170). Ionotropic purinergic receptors P2X_2_ and P2X_3_ are expressed in gustatory nerve fibers in fungiform papillae, and their binding to ATP is an important step in gustation (171). Several primary tastes have been associated with the release of ATP and its binding to P2X_2_ and P2X_3_ (168). Sclafani and Ackroff (122) also reported that naive P2X_2_/P2X_3_ double knockout (DoKO) mice showed significant preference deficits toward fat solutions. However, following exposure to fat, these mice did develop strong preferences for the nontaste qualities of fat, such as texture and odor. The authors suggest that the experience-induced preferences were likely due to postoral conditioning (122).

#### 4.1.3. Drosophila proteins.

The previously mentioned proteins participate in the primary stages of fat detection in taste and smell organs. FFA binding ultimately causes depolarization of gustatory and olfactory receptor cells, which leads to neurotransmitter release at afferent nerve fibers (43). It is important to understand the proteins involved in afferent neurons’ response to dietary FAs. In *Drosophila melanogaster* models, gustatory receptor neurons (GRN) are housed within the gustatory sensilla, act as the primary nutrient-sensing cells, and shed light on proteins involved in rodent and human neurons (127). In the current search, five papers discussed the role of ionotropic receptor (IR) proteins, Gr64, and *DmOrco* in fatty acid taste in *Drosophila* (83, 90, 102, 104, 127).

##### 4.1.3.1. dmorco.

Odorant receptor coreceptor (Orco), which serves as a chaperoning coreceptor for odors, is an olfactory-related molecule involved in sensory-mediated responses to fat in *Drosophila.* In *D. melanogaster,* these olfactory receptors are referred to as DmOrco, are expressed in most olfactory receptor neurons, and are involved in olfactory and nutrient-related signaling (172). A 2018 study by Jung et al. (102) found that a HFD reduced *DmOrco* gene expression by 70% in olfactory neurons and decreased olfactory sensitivity to short-chain fatty acids. This suggests that DmOrco may play a role in the olfactory detection of fatty acids and may be analogous to key olfactory receptors in other species.

##### 4.1.3.2. gr64.

Previous literature describes phospholipase C (PLC) in the fat taste pathway in TB100 (43). It acts to signal inositol triphosphate (IP_3_)-gated calcium channels after TB100 activation (43). A 2018 study by Kim et al. (104) found that novel gustatory receptors, such as Gr64e and Gr64f, ligand-gated ion channels in glycerol detection, may act downstream of PLC in *Drosophila*. Results from gene deletion tests on FFA palatability show that *Gr64e* is involved in fat chemosensation (104). Similarly, Tauber et al. (127) reported that neurons expressing the gustatory receptor *Gr64f* were activated by FFA stimulation and contributed to reflexive feeding responses to fat. Additionally, Brown et al. (90) found that all FA increased *GR64f* neural responsiveness, but the proboscis extension response to medium-chain FA is only controlled by *GR64f*, which suggests that short-, medium-, and long-chain FA taste discrimination occurs through different neural channels. Although only expressed in *Drosophila*, the involvement of these two gustatory receptors highlights the role of PLC in fat chemosensation.

##### 4.1.3.3 ir proteins.

In *Drosophila*, taste neurons express ionotropic receptors (IRs), such as IR56d, which are involved in Ca^2+^ signaling in and activation of gustatory neurons. GRNs expressing the *IR56d* gene are necessary for responding to short- and medium-chain FAs in *Drosophila* (83, 127). Silencing *IR56d* transcripts (83) and IR56d-expressing neurons (127) inhibits *Drosophila’s* ability to detect and respond to FAs. This effect was seen at the cellular and behavioral level (127). Similarly, Brown et al. (90) found that flies can discriminate between short-, medium-, and long-chain fatty acids, but they are unable to discriminate between different compounds of the same fatty acid chain. They found that *IR76b* and *IR25a* are required for taste response to all three fatty acid classes. Additionally, they observed a significant reduction in the proboscis extension response to medium-chain fatty acids when silencing *IR56d*-expressing neurons and *GR64f*-expression neurons indicating that *IR56d*-expressing neurons are required for medium-chain fatty acid taste perception. However, they did not see any significant proboscis extension response to short- and long-chain fatty acids between the *GR64f*-silenced group and the control group (90). Although IR proteins are not found in humans and rodents, these findings reinforce the importance of calcium signaling in fatty acid detection (discussed below).

#### 4.1.4. Signal transduction.

FFAs released following the mechanical and chemical breakdown of foods initiate signaling cascades in gustatory and olfactory tissues. These signaling cascades impact intracellular signal transduction pathways, including calcium and glutamatergic signaling. Intracellular signal transduction pathways are summarized in FIGURE 2.

##### 4.1.4.1. calcium signaling.

Calcium signaling plays a vital role in cellular activities that relay chemical information received by chemosensory cells to the brain (42). In TB100 and OSNs, calcium is released from the endoplasmic reticulum (ER) via IP_3_-gated channels. This triggers the opening and closing of ion channels/exchangers leading to cell depolarization and neurotransmitter release to afferent neurons (43, 173). Five studies included in this review highlight the role of intracellular calcium signaling in fat perception (96, 103, 112, 126, 127).

Subramaniam et al. (126) found that fatty acids (i.e., LA) impact calcium signaling via CD36. They found that CD36 contributed to the opening of calcium channels, such as CALHM1, and that genetic ablation of CALHM1 impaired preference for dietary fat. Furthermore, the ERK1/2-MAPK cascade is regulated by the opening of CALHM1 in TBCs modulating orogustatory detection of lipids in humans and mice (126). Calcium signaling can lead to activation of the ERK1/2 pathway. The impact of ERK signaling on fat taste was examined in a 2017 study by Khan et al. (103). *Erk1* knockout mice (*ERK1*-/-) maintained on HFD exhibited a low preference for dietary fatty acids and developed obesity. Phosphorylation of ERK1 and ERK2 is an important step in the fat taste transduction pathway in TB100 and is needed for dietary fat preference (103). Similarly, a 2018 study by Djeziri et al. (96) reported an association between intracellular calcium and fat preference. They found that oleanolic acid increased intracellular Ca^2+^ concentrations; this change was correlated with increased CD36 mRNA (96). These studies support the role of calcium signaling (via CD36, CALHM1, and EKR1/2 phosphorylation) in fat perception.

Furthermore, a translational 2017 study by Murtaza et al. (112) examined the use of calcium to modify taste and eating behavior in humans. They tested the effects of zizyphin, a triterpenoid and steroid precursor that influences lipid signaling, by recruiting Ca^2+^ from the ER and the extracellular environment via the opening of store-operated calcium channels. When given in conjunction with LA, the zizyphin produced an additive effect on intracellular Ca^2+^ signaling. This increase in intracellular calcium was accompanied by an increase in fat preference (112). This indicates that the intermediate action of calcium signaling is needed for oral fat detection.

Calcium signaling is also involved in the brain’s transduction pathways. Sweet gustatory receptor neurons (GRN) that typically respond to sweet taste are involved in the recognition of fatty acids. Ionotropic receptors IR25a and IR76b signal fat taste recognition in *Drosophila* by triggering intracellular Ca^2+^ increases in tarsal sweet GRNs (127). These mechanisms similarly confirm that calcium signaling is the facilitator of fat taste transduction in neurons.

##### 4.1.4.2. calhmi.

CALHM1 is a downstream calcium ion channel that facilitates the calcium uptake in TBCs and neurotransmitter release (124). Sclafani and Ackroff (124) reported that naïve CALHM1 KO mice displayed deficits in fat preference (even stronger than CD36 KOs) that were rescued following postoral conditioning. CALHM1 channels also influence LA-induced calcium signaling and extracellular signal-regulated kinase 1/2 (ERK1/2) phosphorylation, which ultimately leads to ATP upregulation and neurotransmitter release, including serotonin (115) release (124, 126). Together these findings suggest that CALHM1 is a key downstream calcium signaling mediator and contributes to fat detection and preference.

##### 4.1.4.3. α-gustducin.

α-Gustducin is the α subunit of the gustatory G protein that is associated with GPR120 (116). In GPR-mediated transduction, GPR120 exchanges the inactive guanosine diphosphate (GDP) for a guanosine triphosphate (GTP) molecule on the G protein, allowing it to phosphorylate downstream proteins, including phospholipase C (PLC) (43). Avau et al. (86) investigated the involvement of α -gustducin in nutrient sensing during nutrient excess. They found that α-gustducin KO mice receiving a HFD had decreased weight gain relative to WT mice. This suggests that interfering with α -gustducin gustatory signaling may be implicated in the development of diet-induced obesity (86)

##### 4.1.4.4. trpc3.

Transient receptor potential canonical (TRPC) channels are nonselective cation channels activated by phospholipase C and endogenous diacylglycerol, and they play a role in orosensory detection. Murtaza et al. (114) found that spontaneous liking, preference, and wanting of fatty acid solutions were significantly decreased in *Trpc3*, a TRPC channel subtype. LA-induced Ca^2+^ signaling was also significantly curtailed in *Trpc3* knockout mice compared to WT. The same effect occurred for WT mice treated with a TRPC3 channel blocker. These results point to the involvement of TRPC channels in secondary signaling cascades in response to fat stimuli (114).

#### 4.1.5. Other ingestive cues associated with fat taste and smell.

So far, we have discussed the role of proteins and the anatomy of fat taste and olfaction (70). This section will describe the role of oral chemosensory cues, such as food texture, saliva, and other chemical transduction signals in fat perception. Eating is a dynamic behavior in which food is processed and perceived via mechanical and physiological mechanisms (66). When food is brought into the mouth, it is broken down through mechanical (e.g., mastication and tongue manipulation) and chemical (e.g., salivary enzymes) processes. Altogether, the mechanical and chemical processes that lead to the release and binding of FFA contribute to fat perception (e.g., detection and liking/preference).

##### 4.1.5.1. texture.

The recognition of food texture results from the interaction between oral surfaces and food. Early studies of fat perception proposed that texture rather than fat taste was responsible for fat perception (174–176). Later studies concluded that fat perception was independent of texture, but texture could contribute to fat perception (40) and fat texture is represented by neurons independent of viscosity (177). Three studies in the current review discussed texture in the context of fat perception (49, 59, 76). Grabenhorst and Rolls (59) found that human participants were able to distinguish HF concentrations and rated them as more pleasant in texture than LF stimuli. Similarly, Appelqvist et al. (49) found that HF content contributed to fatty mouthfeel and consumer preference. This is due to increased oral lipid-droplet coating, which can be perceived long after swallowing (49). Additionally, Running et al. (76) demonstrated that degree of saturation influences rejection of chocolate products. The taste and aroma of chocolate with higher concentrations of polyunsaturated FAs were preferred over monounsaturated acid (OLA) (76). Together these studies support that texture contributes to fat perception.

##### 4.1.5.2. saliva.

Saliva initiates the chemical breakdown of food, contributing to oral fat taste perception (70). Saliva is a body fluid consisting of water, inorganic salts, enzymes, and other proteins synthesized and secreted from the salivary glands (178). Five studies in this systematic review examined the role of saliva in fat perception. Voigt et al. (41) found lipases (LIPs), different from LIPF (as observed in rodents), are present in human salivary glands. They also observed that oral perception of triglycerides is associated with differential LIP activities on individual threshold concentrations. Thus this study provided evidence that lingual lipolysis is important for oral perception of fat in humans (41). Méjean et al. (66) found a positive association between the salivary properties (flow, proteolysis, and composition) and liking for fat in humans. Similarly, Mounayar et al. (70) observed that fatty acid sensitivity was associated with changes in saliva composition induced by OLA. Furthermore, Melis et al. (69) found that a small surplus of l-arginine (l-arg) was sufficient to increase OLA perception, suggesting l-arg might contribute to fatty acid perception. Additionally, Besnard et al. (51) reported an association between fat taste sensitivity and the oral microbiome. This suggests that specific microbial communities (e.g., TM7 bacterial family) and salivary signatures (e.g., salivary flow, lysozyme activity, total antioxidant capacity) influence orosensory detection of dietary lipids (51). Together these studies highlight that salivary flow and composition (e.g., proteolytic enzymes, l-arg, and salivary microbiome) may contribute to fat perception.

##### 4.1.5.3. tongue characteristics.

The tongue possesses the papillae and TBCs necessary for chemosensation of food in the oral cavity. Therefore, it is plausible to suggest that individual tongue characteristics can impact the way that one tastes flavors and the intensity of those flavors. Zhou et al. (81) investigated this by correlating fungiform papillae density on the tongues of human subjects to fat taste sensitivity. They found that participants classified as hypersensitive to OA had a higher fungiform papillae density than those classified as hyposensitive to OA. The authors hypothesized that higher fungiform papillae density results in more fat taste receptors in the mouth and increased taste sensitivity to OA (81). Additionally, Liu et al. (65) found gene expression of CD36, FFAR4, FFAR2, GPR84, and KCNA2 in their collected fungiform papillae samples. Following Western blot analysis, they also observed positive gene expression of candidate fat taste receptors and DRK channel proteins in the human fungiform papillae. While the expression level of CD36 presented a negative correlation with the preference to high-fat foods, they found that the expression of FFAR2 in fungiform papillae was related to fat intake.

### 4.2. Neuroanatomy and Physiology of Fat Taste and Smell

**FIGURE 8.**
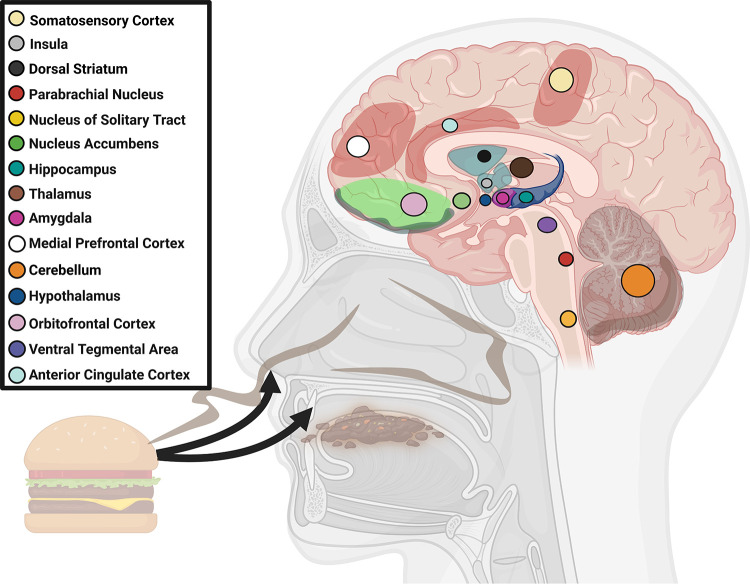
Brain regions involved in fat taste and smell. The perception of fat taste and smell activates of multiple brain regions involved in fat chemosensation. Image created with BioRender.com, with permission.

Although, as previously discussed, taste and olfaction are deeply intertwined, they are two separate senses with their own receptor organs and separate neural substrates. Information from TBCs is relayed to the brain where they are processed and give rise to our perception of taste (179). Olfactory sensory neurons (OSN) are found in the olfactory epithelium that lines the nasal cavity. Olfactory receptors in these sensory cells initiate transduction processes that result in odor perception (180). We provide a brief overview, and a summary of basic gustatory anatomy is provided in FIGURE 8. In general, the perception of taste and smell involves the activation of multiple brain regions. In this section, we discuss advancements in our understanding of the anatomy and physiology specific to fat taste and olfaction (TABLE 4) (57, 59, 79, 87, 94, 97–99, 116).

**Table 4.** Brain regions involved in fat chemosensation

Region	Study (Reference), Year	Finding
Arcuate nucleus	Peterschmitt et al. (116), 2018	When adding LA linoleic acid (LA) on circumvallate papillae on the mouse brain, mRNA expression of Zif-268, brain-derived neuroptrophic factor (BDNF), and Glut-1 occurs on the arcuate nucleus.
Amygdala	De la Cruz et al. (94), 2015	Corn oil induced c-Fos activation on different subareas (basolateral, central-cortico-medial) of the amygdala.
	Eldeghaidy et al. (57), 2016	A high-fat meal caused a reduction on the activation of the amygdala in response to the fat stimulus compared to the no-fat control stimulus (fat-related satiety).
Cerebellum	Sun et al. (79), 2016	Change in odor intensity perception and ghrelin reactivity associated with a negative association in the cerebellum with less cerebellar response.
Cortical (corticolimbic area)	Espitia-Bautista et al. and Escobar (97), 2019	A rat brain on a diet rich on fat promoted high levels of free fatty acids, leading c-Fos to be found higher in ΔFosB in all the corticolimbic areas on comparison with rat brains on a standard diet.
Dorsal striatum	De la Cruz et al. (94), 2015	c-Fos activation was increased on the exposure of corn oil on the dorsal striatum.
Hippocampus	Peterschmitt et al. (116), 2018	On the hippocampus of the mouse brain, mRNA expression increases of Zif-268, BDNF, and Glut-1 when LA is applied on circumvallate papillae.
Hypothalamus	Gaudet et al. (99), 2019	A high-fat diet increased the expression of CD36, serotonin, and variations of tryptophan hydroxylase isoenzymes (TPH1 and TPH2). It also downregulated the expression of the orexigenic hypothalamic neuropeptideY (NPY), which is involved on feeding behavior in the mediobasal hypothalamus (MBH).
	Eldeghaidy et al. (57), 2016	After a high-fat diet, cerebral blood flow (CBF) on baseline resulted on a reduction on the hypothalamus taste.
Insular cortex	Espitia-Bautista and Escobar (97), 2019	A diet rich in fat leads an increase in the number of c-Fos on the insular cortex.
mPFC	De la Cruz et al. (94), 2015	An increased c-Fos expression in the areas of infralimbic and prelimbic mPFC by corn oil intake.
Nucleus accumbens (NAc)	De la Cruz et al. (94), 2015	Corn oil intake increased c-Fos on NAc core but not on the NAc shell.
	Espitia-Bautista and Escobar (97), 2019	A rat brain with a diet rich on fat promoted high levels of free fatty acids, leading c-Fos activation to be found higher in the core and shell NAc regions, than the brains on a standard-rich diet.
	Peterschmitt et al. (116), 2018	There is no significant difference in c-Fos expression after the application of LA seen on a mouse brain.
Nucleus of the solitary tract (NTS)	Peterschmitt et al. (116), 2018	On the mouse brain, c-Fos, Zif-268, and Glut-1 mRNA increases its expression on the NTS with the addition of LA on CV.
Nucleus of the solitary tract (NTS)	Weiss et al. (129), 2019	A high-energy diet produces blunted, but more prevalent, responses in the NTS, and weaker association of taste responses with ingestive behavior.
Orbitofrontal cortex (OFC)	Grabenhorst et al. (59),	Human somatosensory cortex (SSC) activity was strongly correlated with the OFC during consumption of high-fat food.
Parabrachial nucleus (PBN)	Peterschmitt et al. (116), 2018	Increase in c-Fos expression in the mouse brain after LA application.
Ventral tegmental Area (VTA)	De la Cruz et al. (94), 2015	c-Fos activation was observed in the VTA following consumption of corn oil in rats.

mPFC, medial prefrontal cortex.

#### 4.2.1. Neuroanatomy of taste perception of fat.

Taste afferent pathways converge at the nucleus of the solitary tract (NTS) and innervate multiple brain regions. Fat taste is associated with the activation of the gustatory cortex, thalamus, hypothalamus, amygdala, striatum, ventral tegmental area (VTA), and hippocampus (181). The involvement of these regions emphasizes the role of energy balance, reward, emotion, and memory in fat chemosensation. Studies included in this systematic review support the involvement of these anatomical regions in fat perception.

A functional magnetic resonance imaging (fMRI) study found that oral fat texture was represented in the somatosensory insula, the anterior cingulate cortex, and the orbitofrontal cortex (OFC) (174). Building on these studies’ findings, Grabenhorst and Rolls (59) determined that human somatosensory cortex (SSC) is involved in oral fat processing via functional coupling to the OFC, a brain region associated with fat texture. SSC activity was strongly correlated with the OFC during the consumption of HF food. This effect was concentration dependent, as a higher correlation between the SSC and the OFC was observed during consumption of high-fat foods compared to low-fat (LF) foods. SSC activity was also correlated with subjective ratings of fattiness but not of texture pleasantness or flavor. This suggests that the SSC is not involved in hedonic processing but may relay information to the OFC to contribute to valuation of fat taste. Andersen et al. (48) found that significant discrimination in the subject’s electroencephalography (EEG) responses when their anterior tongue was stimulated with skim milk, whole milk, and the intermediate between the two while controlling for confounding somatosensory variables. In these EEG recordings, they observed consistent negative polarizations to the right and left frontotemporal regions 0.1 s after stimulus was applied. Additionally, 0.4 s poststimulus, they observed negative deflections merging at the frontal lobe, and 0.7 s after, it separated back to the frontotemporal regions. This indicated that taste alone can be responsible for changes in the neural circuitry. More recently, Han et al. (61) found that there is stronger insular activation in response to high-fat versus slow-fat odors in individuals with high sensitivity (HS) to sweetness. They also found that individual sweetness sensitivity was positively correlated with insula activation. This suggests that the sweet taste sensitivity may interact with fat chemosensation and that the insula may play a role in fat-odor perception in individuals with HS to sweetness (61). Collectively, these studies highlight the involvement of brain regions involved in higher cognitive functions during fat chemosensation.

Other clinical studies have examined the role of limbic and homeostatic regions in fat chemosensation. Frank-Podlech et al. (58) observed a positive association between oral fat sensitivity and functional connectivity (FC) between homeostatic (hypothalamus) and limbic areas (hippocampus and amygdala) following a HFD. Conversely, they found a negative correlation between the FC between dorsal striatum and somatosensory regions following low-fat stimuli. Finally, they reported that fat ingestion disrupts the connection between the reward and gustatory networks. These data provide evidence for the involvement of brain networks involved in hunger and satiety regulation in oral fat perception.

Preclinical studies have also examined brain regions involved in fat taste, including the insula amygdala, striatum, and VTA. De la Cruz et al. (94) found activation of c-Fos, an indirect marker of neuronal activity, in the striatum, VTA, and amygdala following the consumption of corn oil in rats. Similarly, Peterschmitt et al. (116) also reported increased c-Fos expression in the central amygdala and VTA in response to lingual fatty acid application. Additionally, Espitia-Bautista and Escobar (97) reported that a fat-rich diet stimulated c-Fos activation in rat corticolimbic areas, including the striatum (NAc core and shell) and the insular cortex. Notably, acute HFD exposure induced higher c-Fos activation than sugar-rich diets. Chronic HFD increased binge-type eating behavior, and anticipatory activity associated with increased c-Fos in the corticolimbic system (97). This suggests that fat perception may engage brain regions involved in reward and emotion. Gaudet et al. (99) found that a HFD affected the expression of the fat taste receptor CD36 in TBCs, the hypothalamus, and the ventral striatum (NAc). Peterschmitt et al. (116) also found c-Fos expression in response to lingual fatty acids within the NTS, parabrachial nucleus (PBN), and ventroposterior medialis parvocellularis (VPMPC) of the thalamus, and other regions known to be activated by gustatory signals. Weiss et al. (129) also examined NTS activity. They collected electrophysiological recordings from NTS cells and found that taste response in NTS cells of HFD-induced obese rats was smaller in magnitude, shorter in duration, and occurred at longer latencies compared with that in lean rats. However, a larger proportion of taste-responsive cells in NTS was observed in the HFD-induced rats than in the lean rats, which most likely relates to compensation for the weakened taste response effect of the HFD (129). Together, preclinical studies suggest that the amygdala, VTA, PBN, VPMPC, NTS, striatum, and anterior insula are important in fat chemosensation.

#### 4.2.2 Neuroanatomy of the olfactory perception of fat.

In olfaction, sensory neurons project to the olfactory bulb, the cortex (e.g., OFC), energy balance centers (e.g., the thalamus and the hypothalamus), the limbic system (e.g., the amygdala) and memory-associated regions (e.g., the hippocampus) (180). This is supported by studies included in this systematic review.

One fMRI study examined brain activity in response to fat stimuli in humans. Sun et al. (79) found that in humans, fMRI blood-oxygen-level-dependent responses in the cerebellum were negatively correlated with fat and sugar (e.g., strawberry and cream) odor intensity perception. The results from this study indicated the cerebellum may be associated with fat olfactory perception (79). A 2019 study by Fardone et al. (98) found that mice receiving a moderate HFD (MHF) or HFD showed decreased neuron excitation in the olfactory bulb and lateral glomerulus in response to fatty acid odors. The findings from both studies suggest that neuronal activity in the cerebellum, olfactory bulb, and lateral glomerulus contribute to olfactory-related fat detection. Further studies are needed to elucidate the neurophysiology of fat-related olfaction.

#### 4.2.3. Neuromodulators of fat taste and smell.

##### 4.2.3.1. glutamatergic signaling.

As a palatable macronutrient, fat stimulates reward pathways via glutamatergic circuits. Glutamate is a major excitatory neurotransmitter (182), and in the hypothalamus, glutamatergic signaling can modulate feeding responses (183). *N*-methyl-d-aspartate (NMDA) receptors are glutamate receptors, and previous studies have found that NMDA antagonism reduced food intake and sucrose preference (184). NMDA signaling may be involved in maintaining learned preferences for specific nutrients (e.g., sucrose, fat, and other flavors) (91). However, the role of NMDA receptors in fat perception and their mechanisms are not fully understood. In this review, three studies examined the role of glutamatergic signaling in fat perception (91, 105, 120).

Buttigieg et al. (91) reported that the acquisition of fat preference in young Swiss CD-1 mice is mediated by NMDA signaling. Mice that received systemic MK-801, an NMDA receptor antagonist, demonstrated blunted fat preference. Kraft et al. (105) confirmed these findings in Balb/c and SWR mice. Kraft et al. also found that MK-801 eliminated preference for an intralipid-flavored solution. This suggests that NMDA receptor signaling is needed for the development of fat-conditioned flavor preference. Furthermore, Sasaki et al. (120) determined that intraperitoneal administration of d-serine, an NMDA coagonist in C57BL6/J, inhibited HFD intake and acquisition of HFD preference. Overall, these studies confirm the key role of glutamatergic circuits in fat preference.

##### 4.2.3.2. acetylcholine signaling.

Acetylcholine is a neurotransmitter and an important neuromodulator that regulates synaptic plasticity, synaptic transmission, and neuronal excitability (185). Like glutamate, cholinergic signaling in the hypothalamus can regulate food intake and eating behavior (185, 186). Hence, acetylcholine neurotransmitter function has been studied in the context of fat taste. Iskhakov et al. (101) examined the effect of cholinergic inhibition on intralipid intake in mice. Scopolamine, a rodent-specific cholinergic receptor antagonist, reduced intralipid consumption and prevented the development of fat-condition flavor preferences in three strains of mice (inbred C57BL/6 and BALB/c, and SWR). These results suggest that cholinergic receptor signaling is essential for regulating fat intake and the development of fat taste preference (101).

##### 4.2.3.3. glucocorticoid signaling.

Glucocorticoids are part of the corticosteroid family involved in inflammatory function (187). Glucocorticoid receptors are expressed in mature olfactory neurons (188). Glucocorticoid signaling may be influenced by fat consumption and impact olfactory function. Lacroix et al. (106) examined glucocorticoid receptor (GR) expression in olfactory mucosa and the olfactory bulb. Results showed that obesity-prone rats fed a HFD demonstrated significantly decreased GR expression in the olfactory mucosa and slightly decreased expression in the olfactory bulb. The authors interpret that these decreases in GR expression may induce inflammation (189), apoptotic changes, and reduced olfactory mucosa renewal (190). In turn, these changes may impair olfaction sensitivity and reduce the olfactory perception of fat stimuli (106). Obesity’s effect on fat taste and smell is discussed in sect. 5.

#### 4.2.4. Reward pathways.

Fat is a particularly palatable nutrient and commonly contributes to foods traditionally perceived as enjoyable. Many energy-dense HF foods (e.g., ice cream, fried foods, baked goods) are widely accepted as palatable, activating brain reward circuitry (191). Reward circuits are responsible for conveying pleasure in response to gustatory stimuli. As discussed in sect. 2, brain regions such as the VTA, NAc, and the amygdala are involved in food response and reward processing (192, 193). The following three studies discuss the role of neural reward circuits in fat perception (94, 99, 116).

De la Cruz et al. (94) used corn oil and sugars to measure immunoreactivity in various brain areas that may help mediate fat intake. Corn oil-induced activation in the VTA, PFC, dorsal striatum, NAc, and amygdala; these areas are strongly implicated in reward processing (94). Similarly, a 2018 study by Peterschmitt et al. (116) showed that the application of LA on the circumvallate papillae increased activation in the VTA and central amygdala compared to control. Both areas are associated with emotional processing and food reward (116). Hence, fat can reinforce reward by triggering neural pleasure centers.

Further, a 2019 study by Gaudet et al. (99) showed that chronic access to HFD alters eating behavior and affects the expression of genes related to fat perception (CD36, dopamine transporters, serotonin transporters) in CV, HYP, and NAc. This suggests that HF feeding patterns significantly affect markers of hedonic eating. Therefore, these studies highlight the strong relationship between fat taste and reward. This relationship may propagate via a positive-feedback mechanism, where activated reward systems promote further fat consumption (99).

##### 4.2.4.1. opioid signaling.

The endogenous opioid system within the brain has implications in the reinforcement of palatable food taste. Opioid agonists and antagonists are involved in stimulatory and inhibitory effects of food intake and affect food reward and food preferences (194–196); for reviews, see Refs. 197, 198. In the current search, two papers discussed the involvement of the opioid system in fat preference (118, 119). Sakamoto et al. (119) looked at the effects of nonselective opioid antagonist naltrexone and mu-selective antagonist naloxonazine on both intralipid and sucrose intake. While more significant results were seen in sucrose intake, both naltrexone and naloxonazine antagonists reduced intralipid intake. The nonselective naltrexone also reduced intralipid preference (119). This indicates that the opioid system, and particularly mu-opioid receptors, play a supportive role in fat intake and the development of fat preference. Building on these findings, in 2015 the same group compared the effect of naltrexone and olfactory (ONX) and glossopharyngeal (GSX) nerve transections. Sakamoto et al. (118) found that naltrexone’s inhibitory effect on fat taste preference was primarily seen at higher concentrations of fat solutions, whereas transecting the ONX and GLX nerves reduced fat intake and fat taste preference at lower fat concentrations. This suggests that the opioid receptor system has a significant impact on fat perception at high concentrations (e.g., HF solutions).

### 4.3. Gut-Brain Axis: Postingestive Cues and Fat Taste and Smell

**FIGURE 9.**
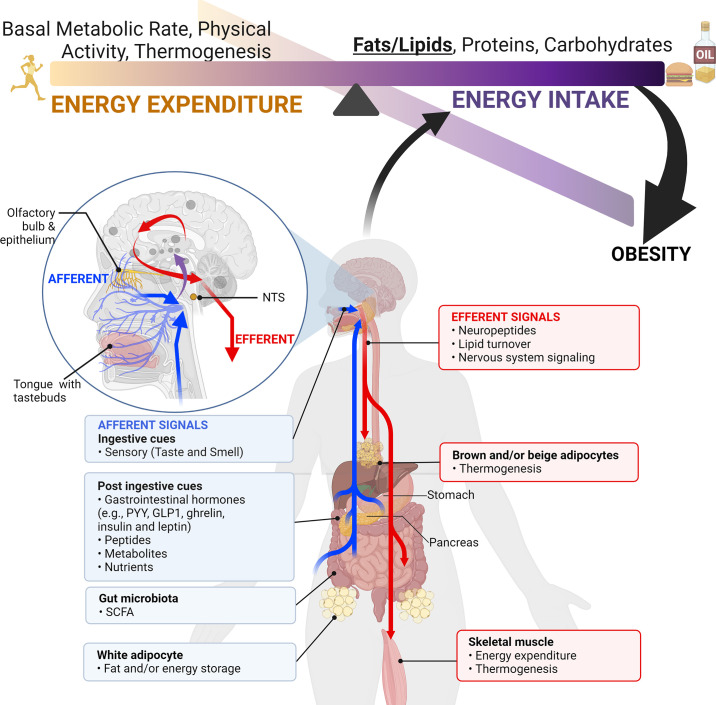
Postingestive and ingestive cues mediate energy intake and energy balance. The brain receives afferent signals including ingestive cues (e.g., taste and smell), and postingestive cues (e.g., hunger and satiety hormones, peptides, metabolites, and nutrient sensing). Together these afferent signals are processed by the brain to inform homeostatic status/energy balance. In response to these cues, efferent signals can stimulate physiological events, including lipid turnover and energy expenditure. Importantly, postingestive cues, can also trigger efferent signals that impact future ingestive cues. For example, an animal or human who may not initially prefer a fatty substance may develop a preference following rewarding postingestive cues. Altogether, these afferent and efferent signals can impact energy intake and excessive energy intake can lead to obesity. NTS, nucleus of the solitary tract; GLP-1, glucagon-like-peptide-1; SCFA, short-chain fatty acid. Image created with BioRender.com, with permission.

Fat perception is not only mediated by the orosensory cues discussed above (e.g., mechanical, and chemical processes) but also by postingestive cues. Postingestive cues are comprised of postoral feedback loops that drive or inhibit food intake (199) (FIGURE 9). These signals are essential for maintaining energy balance. Both the absence and presence of nutrients in the gastrointestinal tract induce the transmission of signals to the brain and other organs (200). This section includes six studies (82, 85, 95, 111, 124, 125) that discuss several postingestive signals, such as insulin, GLP-1, PYY, ghrelin, GPR120, GPR40, and CB_1_R. Here we examine how these postingestive cues (e.g., nutrient-sensing and reward signals) affect fat taste and olfaction.

The importance of postingestive cues in fat perception is captured by studies examining the role of gastrointestinal signals following fat consumption. Mathes et al. (111) found that gastric signals can impact fat preference. They observed that chow-fed rats that underwent Roux-en-Y gastric bypass (RYGB) surgery showed less preference for palatable solutions (e.g., Ensure and intralipid solution) than rats that underwent control surgery. This indicates that the stomach plays a role in fat detection (111). Additionally, gastrointestinal signals can reinforce the drive to consume fat. Ackroff and Sclafani (82) used intragastric (IG) infusions to bypass the oral cavity and isolate the postoral gastrointestinal responses to nutrients in mice. Results showed that IG infusions of lipid solutions generated concentration-dependent enhancement of fat intake and preference (82). While the exact mechanism of these postoral signals was uncertain, the authors proposed that localized suppression of cholecystokinin (activates digestion) by GPR40, GPR120, and other endocannabinoid receptors may promote fat intake (82, 143, 201). Sclafani et al. (82, 143, 201) also observed that postingestive cues played a role in fat preference. They found that prior exposure to fat rescued fat preference in CD36 and CALHM1 knockout mice. In addition, Avalos et al. (85) found that the knockout of CB_1_R endocannabinoid receptors in the upper intestinal epithelium of mice caused an extinction in preference for a HFD compared to control mice. This indicates that postingestive mechanisms were sufficient to develop fat preference (124).

Some studies focused on the importance of specific digestive actions in fat. Dietary fat content is commonly found in the form of triglycerides, composed of a glycerol molecule and three fatty acid tails. In both the mouth and intestine, lipases break down triglycerides into their components so that the FFAs can be easily recognized by receptors (202). Sclafani and Ackroff (125) examined the role of lipolysis in oral and postoral fat perception. They used orlistat, a drug that prevents fat breakdown with (oral and intestinal) lipases. Mice learned a preference for intragastric oil solutions over those paired with orlistat (125). Additionally, these effects were more substantial in intragastric experiments than in oral administration. This indicates that postingestive lipolysis is a necessary pathway to develop fat preference. In a clinical study on 15 adults, Kulkarni and Mattes (64) investigated the role of lingual lipase in oral fat detection for almond butter with and without orlistat. The authors did not find that lingual lipase contributed to oral fat detection. They hypothesized that oral fat detection of fatty foods requires stronger oral processing effort.

Additionally, other studies have shown that postingestive signals’ effect on fat intake and preference is also mediated by satiation. For example, in *Drosophila*, Devineni et al. (95) observed that starving flies showed a robust appetitive response to free fatty acids (i.e., acetic acid), likely reflecting a compensatory mechanism following a state of negative energy balance. Meanwhile, fed flies showed aversion (95). Although satiation signals can acutely inhibit fat intake and preference, prolonged exposure can promote increased fat intake and preferences (203). Ultimately, this suggests that acute postingestive cues are sufficient to impact fat perception based on a fed/fasted state.

#### 4.3.1. Insulin.

As mentioned above, hormones play an integral role in energy balance by eliciting satiation and mediating hunger signals. Insulin regulates the metabolism of fats and proteins by initiating glucose absorption from the blood (204). After ingestion, insulin mediates the conversion of glucose into glycogen or fats (205). Studies included in this review examined the association between insulin, insulin-related proteins, and fat perception. Lacroix et al. (106) found that insulin did not affect obese rats’ olfactory perception of fat. Obesity-resistant rats displayed insulin-dependent changes in odor-sniffing activity; however, intraperitoneal insulin injections (mimicking a meal-induced insulin surge) in obese rats did not alter odor sniffing activity of high-fat diet. This suggests that insulin signaling is involved in the olfactory detection of fats. Additionally, obesity-induced decreased insulin sensitivity may lead to an impaired olfactory perception of fats, leading to disinhibited eating (106).

#### 4.3.2. GLP-1 and PYY.

Additionally, studies examined the role of glucagon-like-peptide-1 (GLP-1), a peptide that reduces blood glucose by enhancing insulin secretion. GLP-1 is an incretin hormone that influences energy intake by reducing glucagon production and slowing gastric emptying after a meal (206). While it primarily acts in the gastrointestinal tract, GLP-1 and its receptor are also expressed in TB100 (207). Studies by Shin et al. (207) and Martin et al. (208) reported that GLP-1 is involved in the general taste pathways and specifically modulates sensitivity. These studies examined traditional modalities such as sweet, sour, and umami; however, GLP-1’s role in fat taste still required investigation. Following this, GLP-1 knockout mouse studies showed that GLP-1 activity could influence fat taste (209). The current search included two studies that provided more specific details on GLP-1’s role in fat taste perception (62, 115).

Ozdener et al. (115) reported that GLP-1 plays an important role in fat taste transduction. Results showed that LA and grifolic acid triggered GLP-1 release from mouse TB100. The authors indicated that GPR120 might initiate this release from lipid rafts (115). These data also suggest that GLP-1 signaling may perpetuate transduction amplification needed specifically for fat taste sensitivity (209). Kadouh et al. (62) examined the influence of liraglutide, a GLP-1 analog, on fat perception. They found that liraglutide (3 mg subcutaneous) modulates taste preference. There was a significant difference between placebo and liraglutide groups in fat taste perception score (determined using the visual analog scale). This suggests that GLP-1 signaling and insulin regulation can mediate fat perception. GLP-1 also was associated with lower levels of plasma peptide YY (210). A previous study by La Sala et al. (211) found that augmenting salivary PYY rescued taste responsiveness to lipids. However, the role of peripheral PYY on fat perception is not fully understood.

#### 4.3.3. Ghrelin.

Ghrelin is a hormone that has been examined in the context of fat taste. Ghrelin is an orexigenic hormone mainly secreted by the stomach during fasting and increases appetite. It promotes meal initiation, influences nutrient sensing, and usually declines after feeding (212, 213). Research in animals has shown that olfactory sensitivity is enhanced in a fasted state (214). However, human research has generated inconsistent results. The following two studies investigated the influence of ghrelin on fat chemosensation (79, 215).

Sclafani et al. (215) reported null findings on ghrelin signaling and postoral effects of fat intake. Results showed that ghrelin receptor signaling was not involved in fat-conditioned flavor preferences (215). A recent paper by Calder et al. (92) also found that male ghrelin receptor Ghsr knockouts (*Ghsr*-/-) mice did not display a reduced aversion to LA following conditioned taste aversion. However, *Ghsr*-/- females showed a reduction in LA taste responsiveness. These data suggest that ghrelin-GHS-R effects on fatty acid taste may be sex specific.

Furthermore, ghrelin has also been studied in the olfactory system. However, Sun et al. (79) focused on the effect of fat perception on metabolic changes. Obese subjects experienced stronger suprathreshold odor intensity of fatty foods (e.g., “chocolate cookie” and “strawberry and cream”) when hungry than when full (79). This was associated with more robust postprandial ghrelin level suppressions and differential cerebellar olfactory responses. (216). These results indicate that ghrelin signaling may impact fatty acid taste and olfactory pathways.

### 4.4. Genetic Polymorphisms

This section will discuss the 10 studies that specifically examined genetic variability associated with fat taste and olfaction (24, 53, 67, 68, 71–73, 77, 78, 80).

#### 4.4.1. CD36.

Single nucleotide polymorphisms (SNPs), variations to a single nucleotide (basepair) within a gene, are the most common type of genetic variation (217). The most frequently studied genetic polymorphisms associated with fat chemosensation are CD36 polymorphisms. The rs1761667, rs1527483, rs2312018, and rs3840546 SNPs of CD36 are reportedly associated with fat perception (TABLE 5). Of these SNPs, the rs1761667 CD36 polymorphism is the most studied in the context of fat taste and has been examined across multiple populations. It has been suggested that the A allele in this SNP is associated with lower oral fat sensitivity, and the G allele is associated with higher oral fat sensitivity (218, 219). However, this association is not fully understood, and a few studies examining the association between the rs1761667 SNP and oral fat perception have conflicting findings (24, 53, 67, 68, 71, 72, 77). The effect of this polymorphism on CD36 function is not fully understood.

**Table 5.** CD36 polymorphisms associated with oral fat perception

SNIP	Study (Reference), Year	Population	Findings
rs1527483	Plesník et al. (73)., 2018	Czech young adults	The participants with the CC genotype of the rs1527483 polymorphism had lower BMI (*P* = 0·011), waist circumference (*P* = 0.005), waist-to-height ratio (*P* = 0.010), and higher sensitivity for linoleic acid (LA) (*P* = 0·037) than the participants with the CT and TT genotypes.
		*N* = 116; 73 females, 43 males	
		Mean age: 21.84 ± 0.22 years	
	Ong et al. (72), 2016	Malaysian adults (293, ethnic Chinese, 20 ethnic Indians)	The overall minor allele frequency for rs1527483 was 0.26. Females and individuals with rs1527483 TT genotype significantly perceived greater creaminess of 10% fat-by-weight custard. Also, individuals with rs1527483 TT genotype and T allele significantly perceived greater fat content of cream crackers, independent of fat concentration. Variants were not associated with obesity.
		*N* = 313; 195 females, 118 males	
		Mean age: 20.73 ± 1.55 (males) and 20.74 ± 1.49 (females) years	
rs2312018	Plesník et al. (73), 2018	Czech young adults	No association was found between the rs3212018 polymorphism and LA detection threshold or anthropometric parameters (i.e., BMI, waist circumference, waist-to-height ratio).
		*N* = 116; 73 females, 43 males	
		Mean age: 21.84 ± 0.22 years	
rs1761667	Bajit et al. (50), 2020	Moroccan adults	There was a higher AA genotype frequency of the SNP in obese subjects, and obese subjects had a significantly higher OA threshold. However, OA thresholds in obese subjects were very dispersed, skewing the results.
		*N* = 100; 72 females and 28 males, mean age 32.37 ± 9.52 years (both)	
	Burgess et al. (53), 2018	Caucasian	Supplementation of oleic acid did not enhance fattiness and creaminess perception for the cohort. However, East Asians carrying the GG genotype perceived more overall fattiness and creaminess than their AA genotype counterparts (*P* < 0.001). No differences were observed for the Caucasians. Thus, variation at rs1761667 may have ethnic‐specific effects on fat-taste perception.
		*N* = 36; 25 females, 11 males)	
		Mean age: 25.3 years	
		East Asian	
		*N* = 32; 24 females, 8 males	
		Mean age: 25 years	
	Karmous et al. (24), 2017	Tunisian adults	A higher AA genotype frequency of rs1761667 was observed in obese subjects compared to normal weight ones (*P* = 0.012). Also, AA genotype (which encodes alanine) of rs1726866 was more abundant in the obese group (*P* = 0.017).
		*N* = 104; 71 females, 33 males	
		Mean age: 35.3 years	
	Karthi et al. (63), 2021	Indian adults	The AA/AG genotype was associated with a higher LA detection threshold.
		*N* = 444; 234 males and 210 females	
	Ong et al. (72), 2016	Malaysian adults (293, ethnic Chinese, 20 ethnic Indians)	The rs1761667 SNP did not significantly affect oral fat perception, except for cream crackers. Variants were not associated with obesity.
		*N* = 313; 195 females, 118 males	
		Mean age: 20.73 ± 1.55 (males) and 20.74 ± 1.49 (females) years	
	Melis et al. (67), 2017	Caucasian Italian adults	The A/G allele of the rs1761667 polymorphism of CD36 was found associated to a distinct metabolic pattern in NW and obese subjects.
		*N* = 126	The G allele of the CD36 gene rs1761667 was associated with increased endocannabinoid plasma levels and a trend for increased waist-to-hip ratio in obese subjects, even though exhibited decreased BMI with respect to those with AA genotype.
		Age not specified	
	Mrizak et al. (71), 2015	Tunisian women	The A allele of cluster of differentiation 36 (CD36) SNP 1761667 is associated with decreased lipid taste perception in obese Tunisian women. Women with the CD36 GG genotype exhibited oral detection thresholds for oleic acid that were more than three times lower than those with the CD36 AA genotype.
		*N* = 203	
		Mean age: 38.4 years	
	Shen et al. (77), 2017	UK adults	No associations were found between CD36 rs1761667 and liking of ice-cream.
		*N* = 136; 95 females, 41 males	
		Age = 50 years (50–year-old cohort of TAMARISK study)	
	Solakivi et al. (78), 2015	Finish adults	CD36 SNP rs1761667 variant AA was significantly associated with lower BMI, compared to variants AG and GG at ages of 40, 45, and 50 years. There was no association CD36 variation with hypertension.
		*N* = 736; 289 females, 447 males	
		Age = 1 cohort at 40, 45, and 50 years of age	

BMI, body mass index; SNP, single-nucleotide polymorphism; NW, normal weight.

Studies included in this review were somewhat consistent with the hypothesis that the G allele of rs1761667 is associated with higher oral fat taste sensitivity than the A allele in specific populations. Melis et al. (67) found that Caucasian adults with the G allele of rs1761667 displayed higher sensitivity to OLA than participants with the AA genotype. Similarly, Burgess et al. (53) found that East Asians homozygous for the G allele of the rs1761667 polymorphism perceived more overall fattiness and creaminess compared to participants with the AA genotype. These GG subjects showed a higher sensitivity to OLA than homozygous AA subjects. However, no differences in fat perception were observed for Caucasians. Thus the authors suggested that the variation at rs1761667 may have ancestry-specific effects on fat perception (53). Similar results were observed by Karthi et al. (63) in a population of Indian adults. Participants with the AA/AG genotype exhibited a significantly higher LA detection threshold and higher BMI than those with the GG genotype (63). Therefore, there is growing evidence that the A allele of the rs1761667 SNP is associated with lower fat taste sensitivity.

However, other studies have reported conflicting findings. A study by Ong et al. (72), which included individuals of East Asian ancestry, also examined the association between rs1761667 and oral fat perception. Malaysian subjects of Chinese and Indian ethnicity were presented with custards of varying fat contents as well as commercially available high-fat foods (i.e., milk mayonnaise, and cream crackers). Ong et al. (72) found a significant association between the rs1761667 SNP and fat taste for cream crackers in the Malaysian subjects. Individuals with the AA genotype of rs1761667 SNP had a lower sensitivity to fat and oiliness of cream crackers. However, they did not find a significant association between the rs1761667 SNP and any other HF tastants (72). A study by Bajit et al. (50) on Moroccan subjects did find an association between the AA and AG genotypes of the rs1761667 SNP and a higher fat sensitivity threshold in subjects with obesity. However, there were large variations in fat sensitivity threshold concentrations for this group, and thus no significant associations were observed. The authors noted that this result was likely due to a small sample size, variations in habitual food consumption, or unfamiliarity with the OA threshold test (50).

Similarly, Shen et al. (77) found no association between CD36 rs1761667 and liking of ice cream in European adults from the United Kingdom. They utilized ice cream as a tastant, which includes a high concentration of fat but also includes a high concentration of sugar and other ingredients (77). Further research comparing individuals of different ancestry is needed to clarify the role of rs1761667 in oral fat perception. Additionally, tastants used have widely varied, so it would be important to utilize more standard tastants across studies.

There is also no consensus regarding the role of CD36 polymorphisms and fat taste in the context of obesity. Mrizak et al. (71) reported an association between the GG genotype of CD36 rs1761667 SNP and decreased lipid taste perception in Tunisian women with obesity. These women had lower (more than 3 times lower) oral detection threshold (higher sensitivity) for OLA compared to women with obesity with the AA genotype. This indicates that those with the AA genotype had lower fat taste sensitivity than the GG genotype and highlights the importance of the rs1761667 SNP in women with obesity. However, as both groups were comprised of women with obesity, it was difficult to determine whether sex or body weight affected oral fat perception (71). A follow-up study by Karmous et al. (24) found that the frequency of the AA genotype of rs1761667 was higher in obese Tunisian men and women compared to their normal-weight counterparts. This further supports the association between rs1761667 and obesity across Tunisian adults (24). This is supported by a study by Melis et al. (68), which found that the G allele of the rs1761667 SNP, compared to the A allele, was associated with a trend for increased waist-to-hip ratio in obese subjects, even though they exhibited decreased BMI (68). This is consistent with a study by Solakivi et al. (78), which found that the AA genotype of rs1761667 was significantly associated with lower BMI, compared to AG and GG variants in middle-aged adults. Additionally, Chmurzynska et al. (55) found that participants with the GG CD36 genotype were more likely to be fat discriminators than participants who were carriers of the A allele, indicating that fat discrimination is associated with CD36 polymorphism. However, they found that fat discrimination was not associated with fat intake and polymorphisms of CD36, FFAR1 (rs1573611), FFAR4 (rs17108973), or CA6 (rs2274333) were not associated with the frequency of high-fat-food consumption (55). However, the Ong et al. (72) study discussed above did not find an association between the rs1761667 SNP, adiposity, or oral fat perception in Malaysian participants (72). Thus the relationship between the CD36 rs1761667 SNP and fat taste remains to be elucidated (68). The role of oral fat perception and obesity is further discussed in sect. 5.

In addition to rs1761667, other CD36 SNPs, such as rs1527483 and rs2312018, have been examined in the context of fat perception. Plesník et al. (73) examined the association between LA detection and rs2312018. No association was found between rs2312018 polymorphisms and LA detection or other anthropometric variables (BMI, waist circumference, and waist-to-height ratio). This suggests that rs2312018 may not be associated with oral fat perception of LA in this population (73). However, further studies should be conducted to better understand the role of rs231018 in oral fat perception.

The CD36 SNP rs1527483 may play a role in oral fat perception and has been linked to high nonesterified fatty acids plasma levels. A previous study was the first to suggest that rs1527483 may play a role in oral fat perception in African Americans (220). Additionally, Chamoun et al. (54) investigated the relationship between single nucleotide polymorphisms in taste receptor genes and psychophysical measures including detection threshold, suprathreshold sensitivity, and fat taste preference. They found that the CD36 SNPs rs1527483 and rs3211908 have significant associations with fat taste sensitivity and preference (54). A later study by Melis et al. (67) found associations between bitter and fat taste sensitivity. They reported that Caucasian participants with a CC genotype of rs1527483, who were also homozygous for the PAV taster variant [associated with super taste perception of 6-n-propylthiouracil (PROP)] of the bitter taste receptor *TAS2R38*, displayed a 5-fold lower threshold (higher sensitivity) for fat. PROP super tasters with the CC genotype of rs1527483 also showed a 4-fold lower OLA threshold (higher sensitivity) than PROP nontasters with the same genotype. However, there was no association (independent of PAV or PROP status) between rs1527483 and OLA detection (67). Ong et al. (72) found that rs1527483 played a more dominant role in fat perception than rs1761667. Ong et al. reported that females with the TT genotype of rs1527483 significantly perceived greater creaminess of a fat-containing custard (10% fat-by-weight). Also, individuals with the TT genotype of rs1527483 significantly perceived greater fat content of cream crackers, independent of fat concentration. This suggests that the TT genotype of rs1527483 is associated with increased oral fat taste sensitivity (72). However, a study by Plesník et al. (73) found that participants with the CC genotype of rs1527483 had higher sensitivity for LA than participants with either a TT or CT genotype. Additionally, unlike Ong et al. (72), who did not find an association between the CC genotype and obesity, Plesník et al. (73) found that rs1527483 was also associated with lower BMI (*P* = 0.011), waist circumference (*P* = 0.005), and waist-to-height ratio (*P* = 0.010). However, Ong et al. (72) and Plesník et al. (73) examined fat sensitivity in different populations (Czech young adults and Malaysian adults, respectively) and utilized different fat-containing tastants (fat-containing foods versus a LA solution). Fat taste sensitivity concerning rs1527483 may vary across populations of different genetic ancestry. Additionally, the combination of fat and other ingredients (e.g., custards and cream) may be perceived differently than fat alone. Thus the association between rs1527483, fat sensitivity, different fat tastants, and ancestry remains to be clarified.

#### 4.4.2. ADRB3.

A study by Watanabe et al. (80) in Japanese young adults found that the TC genotype of the *ADRB3* Trp64Arg polymorphism is associated with a higher preference for high-fat sweet food. These individuals also demonstrated an increased preference for higher degrees of greasiness. This variant is associated with a reduction in noradrenalin-dependent lipolysis activity. The 64th amino acid (Trp) is located on the first intracellular loop of ADRB3 and is essential for the effector and movement function of this protein (80). Therefore, the Trp64Arg variant can lead to protein conformational changes impairing the function of ADRB3. However, the mechanism through which *ADRB3* impacts fat perception is not fully understood.

#### 4.4.3. BDNF.

A study by Graham et al. (60) in a UK female cohort looked at the brain-derived neurotrophic factor (*BDNF*) gene, known to be a regulator of appetite. The aim of their study was to determine whether known genetic polymorphisms related to fat taste and dietary intake would lead to differences in fat taste sensitivity. The study found that participants with the CT/TT genotype of SNP rs6265 *BDNF* had a lower fat taste threshold than the CC genotype, highlighting an association between BDNF and fat taste sensitivity.

#### 4.4.4. CA6.

Polymorphisms of other genes, including the *CA6* gene that codes for the salivary trophin factor Gustin/CA6, have also been examined in the context of fat taste. Shen et al. (77) found a significant association between the GG genotype of *CA6* SNP rs2274333 and liking of ice cream. Subjects with the GG genotype of rs2274333 had a significantly lower liking of the taste of ice cream (a high-fat food) than subjects with the AA or AG genotype. This decreased preference for high-fat food suggests that the GG genotype of rs2274333 may reduce oral fat preference. However, since ice cream also contains high levels of sugar and other ingredients, this preference change may be associated with another ingredient (77). The association between *CA6* polymorphisms and other taste modalities has been examined, and studies have reported an association between CA6 and salt taste but not bitter taste (221, 222).

#### 4.4.5. TNNI3K.

A study by Graham et al. (60) in a UK female cohort examined the association between troponin I-interacting protein kinase (TNNI3K) gene and obesity. Participants with the AA/AG genotype of SNP rs1514175 *TNN13K* exhibited a higher fat taste threshold compared to the GG genotype. Participants with the AA/AG genotype also exhibited higher energy intake, total fat intake, and saturated fatty acid intake (60). This suggests TNN13K may play a role in obesity via mediation of fat taste sensitivity.

### 4.5. Implications of Obesity on Fat Taste and Smell

As discussed above, the sensory characteristics of food, i.e., smell and taste detection, intensity, quality, and hedonic valence, help determine its palatability (223). Additionally, there are documented taste and olfactory changes in individuals with obesity and following weight changes. In turn, taste and smell dysfunction can impact eating behavior. Chronic hyperphagia, alongside altered hedonics and genetic expression, can disrupt energy homeostasis and promote obesity. Individuals with obesity display a lack of postingestion adaptations, unlike their normal weight counterparts. Similarly, a predisposition for fat liking/preference has been observed in overweight individuals and animals. While normal weight individuals demonstrate a decrease in fat preference following food consumption and decreased hunger, individuals with obesity do not display decreased preference even in the absence of hunger (89). However, the relationship between fat taste threshold and intensity is unclear. A recent systematic review and meta-analysis found no significant differences in fatty acid taste threshold or intensity between lean and obese adults (224). Olfactory detection of fat in individuals with obesity compared to lean controls has also been studied, with varying results. While some studies have found an association between obesity and decreased olfactory sensitivity (225), other studies have reported that individuals with obesity had a stronger hedonic response to odors. Thus the association between olfactory sensitivity and obesity is unclear. Nine studies included in this section discuss the influence of obesity on fat chemosensation (52, 56, 74, 75, 89, 96, 98, 106, 226).

#### 4.5.1. Obesity and taste.

Multiple studies have examined the association between fat taste threshold/sensitivity and obesity. For a review of fat taste sensitivity and obesity, see Tucker et. al. (224). In this review, we examine only studies that examined potential biological mechanisms underlying fat taste threshold in lean and obese individuals. Two studies examined potential biological mediators of fat taste threshold. Proserpio et al. (74) found that obese subjects exhibited higher threshold values for fat tastants than normal weight controls and significantly higher liking ratings for high-fat foods. Obese subjects also had a reduced number of fungiform papillae on the tongue. Thus the authors postulated that lower sensitivity may play a causal role in alterations to food preference in obesity (74). Costanzo et al. (56) conducted a randomized controlled trial with twin dyads to examine the effect of dietary fat intake and genetics on fat taste sensitivity. In two groups, 8 wk of LFD consumption increased fat taste sensitivity, while HFD attenuated fat taste sensitivity, regardless of body weight. Overall, the authors suggested that environmental factors and dietary composition, and not genetics, primarily drive dietary fat intake and sensitivity (56). These studies suggest that fungiform papillae density and nongenetic environmental factors may play a role in fat taste threshold in humans.

This review also identified studies that have examined the relationship between fat taste preference and intake and obesity. Djeziri et al. (96) looked at oleanolic acid’s (OLN) effects on obesity and fat preference in mice. They found that HFD-fed mice showed a significantly decreased preference for fat. They further reported that adding OLN to the HFD given to obese mice restored proper orosensory detection threshold for OLA. The authors suggested that improved fat sensitivity was likely due to increased CD36 mRNA expression and intracellular calcium concentration in TBCs (96). This suggests that OLN can help regulate fat taste sensitivity by mediating fatty acid detection. Schreiber et al. (121) examined the role of lingual input on obesity-resistant (OR) and obesity-prone (OP) rats. They found that while in OR rats, fungiform papillae density was higher, and a transection of glossopharyngeal nerves decreased HFD intake. Meanwhile, OP rats had lower papillae density and did not alter their HFD intake following nerve transection. Thus OP rats demonstrated dysregulated orosensory perception of high fat foods. These studies both report and highlight fat taste dysregulation in mouse models of obesity.

Two studies explored the relationship between CD36 expression with fat taste in obesity. Braymer et al. (89) examined the effect of lingual application of CD36 siRNA on fat preference. They measured LA preference in obesity-prone (OP) and lean obesity-resistant (OR) rats. While in OR rats, lingual application of CD36 siRNA decreased fat preference, OP rats did not decrease their preference for LA. This suggests that OP rats do not alter their fat preference based on nutritional status or changes in lingual CD36 levels. The relationship between CD36 and fat preference was also investigated following fasting. Fasting increased lingual CD36 mRNA in OR rats, but not in OP rats, and postfasting application of CD36 siRNA decreased LA preference in OR but not OP rats. These results support that CD36 is essential in regulating fat preference and demonstrate that obesity is associated with altered fat taste, likely mediated by CD36 dysregulation (89). The relationship between fat taste, CD36, and obesity has also been studied in humans. A 2019 cross-sectional study by Bricio-Barrios et al. (52) measured the correlation between CD36 and fat taste sensitivity, adiposity, and BMI. They showed that serum levels of the soluble form of CD36 (sCD36) are positively correlated with FFA sensitivity and negatively correlated with adiposity and BMI. Specifically, overweight subjects exhibited a higher threshold (lower sensitivity) for FAs and lower serum sCD36 levels than the normal-weight group (52). The results of both studies demonstrate that obesity can modulate the effect of CD36 on fat taste sensitivity and preference.

#### 4.5.2. Obesity and olfaction.

Multiple studies have investigated how obesity can affect fat olfactory function. Lacroix et al. (106) compared olfactory performance and tissue homeostasis between DIO OP and lean normal chow-fed OR Sprague-Dawley rats. Results showed that OP rats experienced decreased odor threshold (higher sensitivity) but lowered olfactory performance and related memory/learning deficiency. Moreover, the authors found differences in olfactory mucosa and bulb homeostasis, electrical olfactory signaling, and cellular dynamics between the OP rats and control. The authors suggest that obese pathophysiology may significantly influence perturbed olfactory satiety signals and resulting food intake (106).

Similarly, Fardone et al. (98) looked at the impact of obesity on OSNs in mice. DIO mice fed MHF and HFD showed reduced neural activity in juxtaglomerular cells. They also identified an asymmetry in the responsiveness of the ‘mirror image’ glomerular map for the M72 receptor that shows greater sensitivity of the lateral versus medial glomerulus toward fatty diets. DIO also led to decreased OSNs that expressed olfactory marker protein (OMP) (98). OMP is important in OSN development, signaling transduction, and responsiveness to and discrimination of odors (227, 228). While the sensitivity results from LaCroix et al. (106) differ from these findings, their olfactory performance results support the mechanistic changes found in this study (98). These results indicate that DIO and high-fat intake significantly impairs OSN function and survival, ultimately dampening olfactory sensitivity to fats and other odors (98). Thus Lacroix et al. (106) and Fardone et al. (98) observed differences in the olfactory bulb and neurons projecting to the olfactory bulb in DIO mice, highlighting the importance of the olfactory bulb to olfactory perception of fat.

Meanwhile, Boone et al. (88) found that anosmic mice (following complete removal of the olfactory bulb) and sham mice (with intact olfactory bulbs) both displayed comparable HFD intake. The authors also observed that a HFD smell (in the absence of consumption) did not alter feeding or devaluation of standard food. Thus, while the olfactory bulb may play a role in fat olfaction, it may not be necessary for the development of a HFD preferential consumption (88). It is important to note that while Boone et al., postulated that olfaction is not necessary for fat preference or devaluation of standard food, this does not entail that the olfactory bulb and olfaction do not play a role in fat preference or detection. Rather, these findings highlight how much emphasis the body places on fat chemosensation; multiple systems have developed in parallel to ensure the consumption of this important macronutrient. As mentioned in the preceding sections, a predilection for fat can develop in response to other nonolfactory cues, such as oral cues and postingestive cues.

Besides transduction pathways, genetic and epigenetic changes can regulate olfactory function, impacting eating behavior and dietary intake. Ramos-Lopez et al. (75) used roughly 500 human subjects from the Methyl Epigenome Network Association to understand the relationships between olfactory genes and clinical variables indicative of obesity. Their results identified several associations between olfactory pathway gene methylation patterns, dietary intake, and anthropometric measures (BMI and waist circumference), independently of age and sex. These genes included olfactory receptors (e.g., OR4D2, OR51A7, OR2T34, OR2Y1) and downstream signaling molecules (e.g., SLC8A1, ANO2, PDE2A, CALML3, GNG7, CALML6, PRKG1, CAMK2D). Pathway enrichment analyses revealed that these genes were significantly involved in odor perception and olfactory cascade signal transduction. Specifically, methylation patterns of OR4D2 and OR2Y1 were strongly correlated with daily energy and macronutrient intakes. These data signify that dietary habits and weight status may significantly impact olfactory gene methylation and olfactory function (75). Olfactory and taste-related genes are also associated with transgenerational nutrient sensing. Ng et al. (226) found that paternal HFD caused downregulation of 187 olfactory transduction *Olr* genes in retroperitoneal white adipose tissue and pancreatic islets of female rats. This suggests that olfaction pathways may be significantly impacted by paternal obesity. The genes susceptible to epigenetic modification may lead to odor perception dysfunction, ultimately leading to changes in eating behavior and body weight regulation (75). In turn, these genetic modifications could increase propensity for obesity and its comorbidities. Further studies in clinical populations are needed to examine the heritability of olfactory genes and their association with eating behavior and obesity.

#### 4.5.3. Limitations.

This systematic review’s search strategy was formulated by experienced chemosensory researchers and a library specialist with expertise in systematic reviews. Our results are limited by our inclusion and exclusion criteria. This includes the sole inclusion of studies published in English, which would fail to capture non-English literature and would be subject to bias against studies contributed from specific nations or regions. Furthermore, our search strategy yielded a wide variation of clinical and preclinical studies, with heterogeneous variables of interest, methodologies, and populations. Thus, although we collected rich data through this comprehensive systematic review, we were unable to conduct a meta-analysis.

Importantly, we observed a lack of standardized reporting of methodology and results across many of the animal studies. For example, many did not report randomization and/or blinding and/or provide detailed statistical methods. The risk of bias is reflected in the SYRCLE scores, with 78% of the studies scoring below 10 (out of a total possible score of 20). However, we chose to include all the animal studies, despite their low SYRCLE scores, because it may reflect reporting inconsistencies across animal studies rather than the quality of the science. Thus this should be considered when interpreting the preclinical results reported in the present systematic review. The observed limitations in animal studies have been identified, highlighted, and reported in multiple systematic reviews (229–231). This has led to journals requiring the use of reporting guidelines, such as the ARRIVE (Animal Research: Reporting of In Vivo Experiments) guidelines. However, it is important that authors, journals, and reviewers adhere and ensure adherence, respectively, to ARRIVE or similar reporting guidelines. This is important to improve transparency in experimental design, reporting, and statistical analyses of animal studies to increase reproducibility and facilitate systematic reviews and meta-analyses in the future. This would strengthen not only the validity and rigor of the studies but would also be an important step toward better interpreting and translating preclinical studies into clinical efforts.

## 5. CONCLUSIONS

The present systematic review highlights a growing interest in fat chemosensation in the context of eating behavior and obesity. Multiple studies examined the role of several proteins in fat chemosensation, including CD36 and GPR120, which act together to detect and/or mediate fat detection by TBCs and OSNs. The involvement of CD36 in fat chemosensation is supported by studies that have found that genetic variations of CD36 (e.g., rs1761667, rs1527483, and rs2312018) can significantly impact individuals’ fat sensitivity and preference. On a cellular level, studies have begun to identify and map signal transduction pathways involved in fat chemosensation. These pathways are initiated by the binding of FFAs to fat chemoreceptors and activate downstream signaling cascades, involving calcium signaling, and leading to cellular depolarization of TBCs and olfactory-related cells. Furthermore, multiple brain regions (e.g., mPFC, OFC, striatum, amygdala, VTA, and somatosensory cortex), neural signaling pathways, and neuromodulators regulate the hedonic properties of fat. Subsequently, hormones, ingestive, and postingestive signals (e.g., insulin, GLP-1, PYY, ghrelin, and serotonin) can reinforce or inhibit the hedonic properties of fatty acids and contribute to fat perception and consumption. As more studies examining fat taste and smell emerge, we hope to learn more about the biological mechanisms underlying fat chemosensation. Understanding these complex interactions is important as we continue to better understand the multifactorial nature of eating behavior and obesity. Identifying and investigating these mechanisms can be important steps in developing management strategies and/or interventions to treat obesity and associated comorbid conditions.

## SUPPLEMENTAL INFORMATION

10.6084/m9.figshare.20415975.v1 Supplementary material available at DOI: https://doi.org/10.6084/m9.figshare.20415975.v1.

## GRANTS

P.V.J. is supported by National Institute of Alcohol Abuse and Alcoholism Grant Z01AA000135, the National Institute of Nursing Research, and the Rockefeller University Heilbrunn Nurse Scholar Award. P.V.J. and H.A.T. are supported by the Office of Workforce Diversity, National Institutes of Health Distinguished Scholar Program. H.A.T. is supported by the National Institute on Mental Health and the Brain and Behavior Foundation Young Investigator Award. R.B.J., C.V., and K.A. received postdoctoral Intramural Research Training Awards, Office of Intramural Training & Education. R.S.E.O.-F., B.E.B., M.C., and N.N. received Postbaccalaureate Intramural Research Training Awards, Office of Intramural Training & Education, and N.I. received a summer fellowship award, Office of Intramural Training & Education, National Institutes of Health, Department of Health and Human Services.

## DISCLOSURES

No conflicts of interest, financial or otherwise, are declared by the authors.

## AUTHOR CONTRIBUTIONS

R.B.J.-T., C.V., A.A.L., H.A.T., and P.V.J. and conceived and designed research; R.B.J.-T. and A.A.L. analyzed data; R.B.J., B.E.B., C.V., M.C., N.N., R.S.E.O.-F., A.A.L., K.A., C.C.-P., N.I., A.H., H.A.T., and P.V.J., interpreted results of experiments; R.B.J.-T., B.E.B., C.V., K.A., C.C.-P., and N.I. prepared figures; R.B.J.-T., B.E.B., C.V., M.C., N.N., R.S.E.O.-F., A.A.L., K.A., C.C.-P., N.I., H.A.T., and P.V.J. drafted manuscript; R.B.J.-T., B.E.B., C.V., M.C., N.N., A.A.L., K.A., C.C.-P., A.H., H.A.T., and P.V.J., edited and revised manuscript; R.B.J.-T., C.V., M.C., N.N., R.S.E.O.-F., A.A.L., K.A., C.C.-P., N.I., A.H., H.A.T., and P.V.J. approved final version of manuscript.
